# ROS networks: designs, aging, Parkinson’s disease and precision therapies

**DOI:** 10.1038/s41540-020-00150-w

**Published:** 2020-10-26

**Authors:** Alexey N. Kolodkin, Raju Prasad Sharma, Anna Maria Colangelo, Andrew Ignatenko, Francesca Martorana, Danyel Jennen, Jacco J. Briedé, Nathan Brady, Matteo Barberis, Thierry D. G. A. Mondeel, Michele Papa, Vikas Kumar, Bernhard Peters, Alexander Skupin, Lilia Alberghina, Rudi Balling, Hans V. Westerhoff

**Affiliations:** 1 Infrastructure for Systems Biology Europe (ISBE.NL), Amsterdam, The Netherlands; 2 Luxembourg Centre for Systems Biomedicine, University of Luxembourg, Esch-sur-Alzette, Luxembourg; 3 Molecular Cell Physiology, VU University Amsterdam, Amsterdam, The Netherlands; 4 Synthetic Systems Biology and Nuclear Organization, Swammerdam Institute for Life Sciences, University of Amsterdam, Amsterdam, The Netherlands; 5 Environmental Engineering Laboratory, Departament d’Enginyeria Quimica, Universitat Rovira i Virgili, Tarragona, Spain; 6 Infrastructure for Systems Biology Europe (ISBE.IT), Milan, Italy; 7 SysBio Centre of Systems Biology (ISBE.IT), University of Milano-Bicocca, Milan, Italy; 8 Laboratory of Neuroscience “R Levi-Montalcini” Dept of Biotechnology and Biosciences, University of Milano-Bicocca, Milan, Italy; 9 Luxembourg Institute of Science and Technology (LIST), Esch-sur-Alzette, Luxembourg; 10 Department of Toxicogenomics, GROW School for Oncology and Developmental Biology, Maastricht University, Maastricht, The Netherlands; 11 Department of Molecular Microbiology & Immunology, Johns Hopkins Bloomberg School of Public Health, Baltimore, MD USA; 12 Systems Biology, School of Biosciences and Medicine, Faculty of Health and Medical Sciences, University of Surrey, Surrey, UK; 13 Centre for Mathematical and Computational Biology, CMCB, University of Surrey, Surrey, UK; 14 Infrastructure for Systems Biology Europe (ISBE.IT), Naples, Italy; 15 Department of Mental and Physical Health, University of Campania—L. Vanvitelli, Napoli, Italia; 16 IISPV, Hospital Universitari Sant Joan de Reus, Universitat Rovira I Virgili, Reus, Spain; 17 Faculty of Science, Technology and Communication, University of Luxembourg, Esch-sur-Alzette, Luxembourg; 18 Manchester Centre for Integrative Systems Biology, School for Chemical Engineering and Analytical Science, The University of Manchester, Manchester, UK

**Keywords:** Biochemical networks, Complexity, Differential equations, Emergence, Computer modelling

## Abstract

How the network around ROS protects against oxidative stress and Parkinson’s disease (PD), and how processes at the minutes timescale cause disease and aging after decades, remains enigmatic. Challenging whether the ROS network is as complex as it seems, we built a fairly comprehensive version thereof which we disentangled into a hierarchy of only five simpler subnetworks each delivering one type of robustness. The comprehensive dynamic model described in vitro data sets from two independent laboratories. Notwithstanding its five-fold robustness, it exhibited a relatively sudden breakdown, after some 80 years of virtually steady performance: it predicted aging. PD-related conditions such as lack of DJ-1 protein or increased α-synuclein accelerated the collapse, while antioxidants or caffeine retarded it. Introducing a new concept (aging-time-control coefficient), we found that as many as 25 out of 57 molecular processes controlled aging. We identified new targets for “life-extending interventions”: mitochondrial synthesis, KEAP1 degradation, and p62 metabolism.

## Introduction

Reactive oxygen species (ROS) are produced at various intracellular locations including complexes I and III of the electron transport chain (ETC) in the inner mitochondrial membrane^1,2^. In all, 0.1–2% of ETC electrons escape to form ROS. This percentage increases with increasing damage in Complex I^3^. Excessive ROS is removed enzymatically, e.g., by superoxide dismutase^4^, or scavenged by various antioxidants^5^. Should these processes fail, the removal of damaged mitochondria, called mitophagy^6–10^, may be initiated^11–13^, or damaged mitochondria may be recycled into undamaged mitochondria via mitochondria-derived vesicles. Both these phenomena should then provide a defense mechanism versus a subsequent, perhaps even stronger, ROS challenge. The mitophagy should then prevent apoptosis or necrosis from happening through a mitochondrial suicide and is then called “mitoptosis”^11^. This in turn provides a network mechanism for the preconditioning effect of cardiac ischemia^11,14^.

Transient increases of ROS production play various roles in cell physiology^2^: They enact intracellular signaling that regulates immune responses^3^, cell differentiation, and proliferation downstream of neurotrophic and growth factor signaling^2,6–11,15,16^. Moderate ROS levels modulate metabolism and activate transcription of detoxifying enzymes such as superoxide dismutase^17^, which, like the mitophagy mentioned above, may provide resistance against subsequent stronger threats^18–21^. These mechanisms have been put in the context of the effects of caloric restriction and called “mitochondrial hormesis”, or “mitohormesis”^22–25^, in which mild stresses leading to ROS production can induce adaptive defense responses and stress tolerance. Paradoxically the ROS thereby appears to extend lifespan and to reduce age-related pathologies such as neurodegenerative disorders and cardiovascular diseases in cellular and animal models^18,20,23,24^. Depending on stress intensity and intervals between stimuli, repetitive or chronic stresses may however compromise these protective responses and thereby reduce lifespan, as observed in Drosophila^19^.

This paradoxical effect of ROS comes on top of a perhaps equally confounding circular causality in which ROS cause aging that causes more ROS and hence more aging: In the current mitochondrial free radical theory of aging, accumulating oxidative damage causes the aging process^26^. ROS-mediated modification of proteins, lipids, carbohydrates, and nucleic acids alters their stability and function^27,28^. Endogenous ROS accumulation and oxidative stress should thereby contribute to genomic instability, accumulation of misfolded proteins, and alteration of several processes (such as proteostasis, autophagy, mitochondrial function) associated with age-related pathologies^28,29^. This phenomenon may be particularly relevant in the brain: because of the prevalently oxidative metabolism required to fulfill their massive Gibbs energy requirements throughout life, neurons have a high rate of ROS production.

Meanwhile the cell has a sophisticated defense network against ROS: ROS-activated signaling networks evoke complex cross-talk^17,30,31^, address p62 and its role in mitophagy, the Keap1/Nrf2 axis, NFκB in modulating antioxidant responses, as well as the stress sensor DJ1^32–37^. ROS damage also activates scavenging systems^34,35,38,39^.

The ROS damage that escapes the defense system may enhance ROS generation however: Older rats show unaltered respiration and mitochondrial activity, but increased 3-nitrotyrosine (3-NT) and protein carbonyls (PC) in synaptic mitochondria^27^. And worse, ROS damage may compromise the regulatory networks controlling the cellular ROS_defence^34,35,40^: Levels of ROS scavenging systems decreased with age in animal models and in humans^41–44^, as did the expression/activity of molecules such as Nrf2^34,35^. Thus, although over-activation of Nrf2 might have deleterious consequences in terms of embryonic lethality and cancer prognosis^45,46^, the reduced Nrf2 activity caused by aging-associated ROS damage may well allow for more ROS to accumulate because ROS scavenging is activated less^34,35,39,47^. This then closes the positive feedback loop from ROS through aging to ROS. These complex networks appear to be sex-, species-, and cell-type dependent^34,35,40,48^.

The dynamic response of this network to perturbations is determined by nonlinear interactions producing the functionality that is absent from its components. This means that the functionality emerges from interactions. Disease may thereby correspond to failure of the network to produce the functionality, e.g., both the robustness to the external perturbations and the failure thereof can be caused by various combinations of component failures^49,50^. Mistuning of ROS management and decreased ATP production has been implicated in aging^51^ and in diseases such as diabetes^52,53^, cancer^54^, and in neurodegeneration^30,31,55^.

The ROS management network contributes a *disease module*^56,57^ to disease maps such as that of Parkinson’s disease (PD)^58^, which show how most features of disease are known to be connected. Affecting 1–3% of the population over 65 years old, PD is characterized by symptomatic motor dysfunction and alteration of the mood/reward system due to lack of dopamine secretion by dopaminergic neurons^59^. PD is a multifactorial disease; it has been associated with diverse genetic and environmental factors. Among recessive PD-related mutations^60^, those in α-synuclein^61^ and Park7 (DJ-1)^33^ are prominent. With respect to environment and nutrition, PD risk correlates positively with pesticide exposure^29^, and negatively with coffee consumption^62–65^. On the anatomical level, the disease may be attributed to processes inside the dopaminergic neurons in the *substantia nigra*, to disruptions of communication between neuronal and glial cells (connected to neuroinflammation), or even to the pathogenic spread of unfolded α-synuclein or tau proteins between cells^29,66–69^.

Network functionality may be reconstructed by translating network information into mathematical equations^70–72^ and may then be replayed in silico to identify “designs”, i.e., network patterns responsible for functionalities. The robustness of these designs to perturbations associated with disease may then be calculated^73,74^, identifying the various possible network causes of the disease. Robustness of the difference between disease and health to perturbations may also be calculated, thereby highlighting network-based targets for drug, nutrition, or lifestyle therapies. Both calculations should be able to deal with differences between patients, and ultimately facilitate individualized medicine. Continuing on modeling of aging by others^75,76^, the present paper implements the first parts of this approach, with the aim of understanding more of the complexity of ROS management in the context of PD.

The “Results” section is divided into three subsections. In the first subsection we discuss the risks, risk-management principles, and a remaining liability in core models of increasing complexity, meanwhile identifying five types of dynamic robustness, five network designs, and five corresponding regulation modes that establish those five types of robustness in the ROS network. In the second section we detail the dynamic model to make a more comprehensive model, validate it partially, and re-examine how the five network designs function in the complex integral network. The final subsection of the “Results” section uses the comprehensive model to assess the “time warp” of aging, to identify targets for nutrition and pharmaceutical therapies of PD, as well as to examine whether such therapies could be personalized.

We conclude that we now have a partly validated dynamic model that is able to (i) address aging and PD as results of networked molecular processes going awry, (ii) elucidate the corresponding time warp, and (iii) enable analyses towards individualized medicinal and nutritional therapies.

## Results

### Risk, management, and a remaining liability

We set out by re-examining the scientific literature looking at the networks around ROS and mitochondria, perusing the Parkinson’s Disease map by Fujita et al.^58^. This map is complex. It has a large number of components that are strongly networked. One might wonder about the biological function of each component. It was the challenge taken up by the present work to try to understand the function of more than 100 components and parameters in terms of a much lower number (say 5) of subnetworks, each securing an identifiable function. We shall call these subnetworks “designs” because they each correspond to a function.

### The risk: the positive feedback loop formed by impaired mitochondria and ROS constitutes a liability inherent in aerobic metabolism

**Fig. 1:**
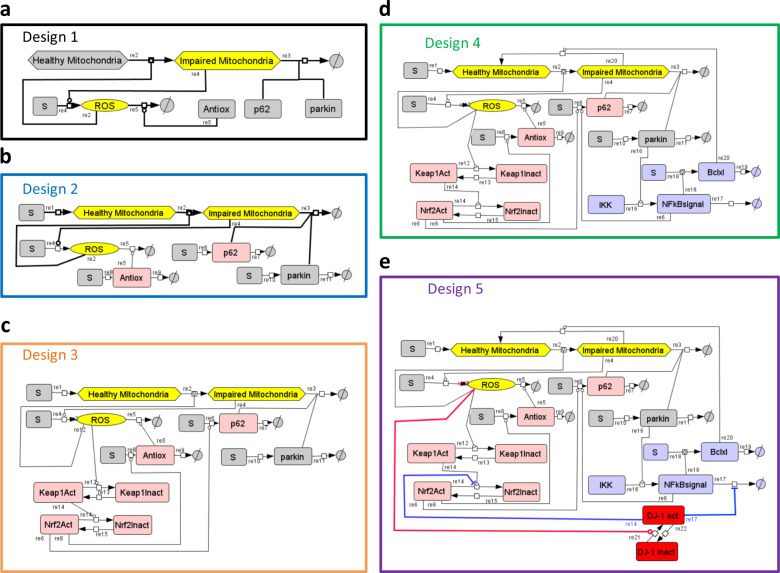
Network diagrams describing five ROS-management designs. Design 1 is comprised in Design 2 and in Design 3, Design 3 in Design 4, and Design 4 in Design 5, thereby forming a hierarchy of networks and corresponding models. **a** Design 1: Steady but not yet robust. Healthy mitochondria present at a fixed concentration are damaged by ROS and thereby converted into impaired mitochondria by reaction 2 (re2). Impaired mitochondria produce ROS (re4). Impaired mitochondria and ROS hereby constitute a positive feedback loop. The node called Antiox comprises the total pool of all antioxidant response elements, i.e., both metabolites (e.g. glutathione) and enzymes (e.g. superoxide dismutase) catalyzing ROS removal (re5). p62 and parkin are required for mitophagy (removal of impaired mitochondria, re3) and are removed together with impaired mitochondria in the process (re3). **b** Design 2: Robust but not yet homeostatic. Here, healthy mitochondria occur at a variable rather than fixed concentration (substances at variable concentrations are shown in non-gray boxes; also Antiox and p62 were made variable here, but without effect in this design) and a reaction where they are synthesized at a constant rate (re1) has been added to Design 1. **c** Design 3: Homeostatic yet potentially oscillatory. Three negative feedback loops have been added to Design 2 (ROS species on the diagram). The new variables Keap1 and Nrf2, in both active an inactive form, involve the Antiox, p62, but not (yet) parkin, which was made variable, i.e., subject to synthesis and degradation reactions, but is not colored in this figure because this had no effect as these reactions were not affected by the rest of the network. ROS oxidize and thereby inactivate Keap1 (re12), which is slowly re-reduced (re13). Active Keap1 shifts the Nrf2 balance towards inactive Nrf2 (re14). When active, Nrf2 activates the synthesis of both p62 (re6) and Antiox (re8), which causes breakdown of ROS (re5). Thus, ROS activates Nrf2, Antiox, and p62, and inactivates Keap1 and itself: this forms a dual ROS-regulating negative feedback loop. Removal of damaged mitochondria causes a reduction in parkin levels, which reduces the removal rate of impaired mitochondria, which constitutes another negative feedback loop. **d** Design 4: Dynamically robust yet fragile vis-à-vis repeated challenges. Mitochondrial repair via the NFκB signaling system (violet species on the diagram) has been added to Design 3: Parkin activates NFκB signaling via IKK (re16). NFκB signal activates the synthesis of Bclxl (re18) and p62 (re6). Bclxl activates the protection of mitochondria by salvaging impaired mitochondria (re20). **e** Design 5: Robust against repeated dynamic challenges. The DJ-1 module (red species on the diagram) has been added to Design 4 as a ROS sensor. DJ-1 is a protein that may be present in two conformations: active (oxidized) and non-active (reduced). DJ-1 activation is catalyzed by ROS (re21). When active, DJ1 inhibits removal of NFκB signal (re17) in sub-design 5.1, inhibits inactivation of Nrf2 (re14) in sub-design 5.2, or regulates both NFκB and Nrf2 signaling pathways in sub-design 5.3. Sub-design 5.3 is the complete version of Design 5, where, upon an increase of ROS concentration, DJ-1 simultaneously activates both the antioxidant response and mitophagy (via Nrf2 and p62) and mitochondrial repair (via NFκB and BclXl).

**Fig. 2:**
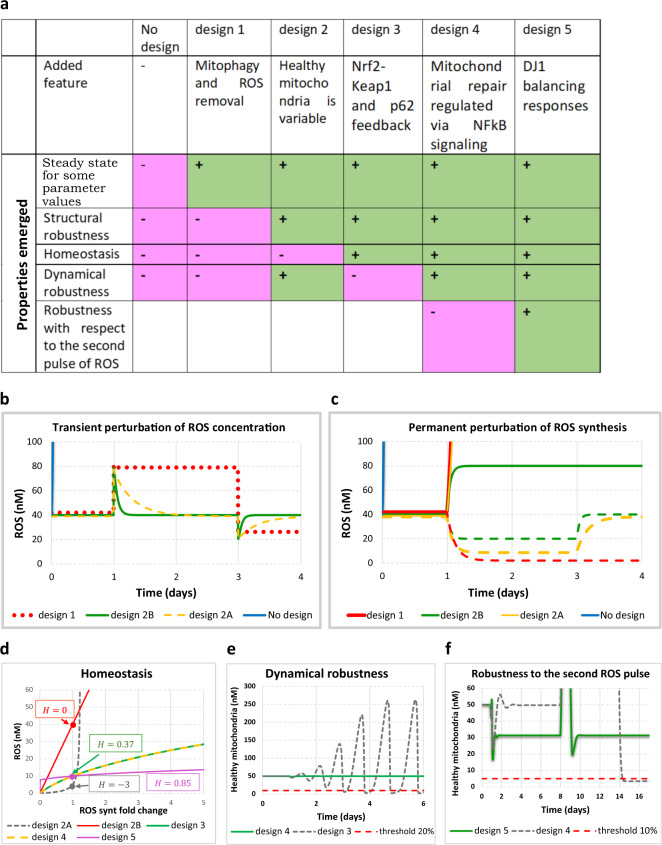
Robustness properties emerging from the five network designs. **a** The table summarizing added features and new emergent properties gained at every design. **b** The response to perturbations of ROS concentration in Design 1 (model B1), and Design 2 (model B2B). ROS concentration (in nM) is shown for the case of “no design” (solid blue line), which increases rapidly from 0 at time zero to over 100nM within 1h. For numerical reasons, then the simulations was stopped, Design 1 (dotted red line) and Design 2 (solid green line). Both ROS injection (doubling of the initial ROS concentration at day −1, or a decrease in ROS level (the ROS concentration was decreased to 20nM at day −3 was applied. All models are described in detail in Supplementary Information (Sections B1 and B2B). **c** The response to perturbations of ROS generation in Design 1 (model B1) and Design 2 (model B2B). ROS concentration (in nM) is shown for the case of “no design” (solid blue line), Design 1 (red lines), and Design 2 (green lines). The ROS generation rate constant was either increased twofold on day 1 (responses are shown in solid lines: solid red line for design 1; solid green line for design 2) or first decreased twofold on day 1 and then returned back to the initial value on day 3 (responses are shown in dashed lines: dashed red line for design 1; dashed green line for design 2). All models are described in detail in Supplementary Information (Sections B1 and B2B). **d** Emergence of homeostasis in Design 3 (model B3). The steady-state concentration of ROS (in nM) is shown against the fold change of the rate constant of ROS generation for Designs 2A (dashed gray line), Design 2B (solid red line), Design 3 (solid green line), Design 4 (dashed yellow line), and Design 5 (purple line). Homeostasis coefficients (*H*) for each design (shown in boxes) were computed at the point where ROS synthesis fold change was equal to 1. Model is described in Supplementary Information (Section B3). **e** Emergence of dynamic robustness in Design 4 (model B4). The concentration of healthy mitochondria (in nM) is shown for Designs 3 (dashed gray line) and Design 4 (solid green line). The initial ROS concentration was perturbed (transient increase from 10 to 11nM) at day 1. Dashed red line shows a hypothetical viability that corresponds to the threshold line dissecting 20% (i.e. 10nM) of the initial concentration of healthy mitochondria (i.e. 50 nM). Model is described in Supplementary Information (Section B4). **f** Emergence of dynamic robustness vis-à-vis with respect to the second pulse of ROS in Design 5 (model B5). The concentration of healthy mitochondria (in nM) is shown for Designs 4 (dashed gray line) and Design 5 (solid green line). The ROS generation rate constant was increased 15-fold on day 1, but, 3h before the increase of ROS generation, the NFκB signaling was increased 15-fold and the system reached a new steady state. On day 8 the ROS generation rate constant was decreased 15-fold causing the growth of healthy mitochondria. At the time point when the concentration of healthy mitochondria was near its peak value, the ROS generation rate constant was increased 15-fold for the second time. Model is described in Supplementary Information (Section B5).

The core of the ROS network (Fig. 1a and Supplementary Fig. A.1, Model A, so-called “no design”) consists of healthy mitochondria, damaged mitochondria producing ROS, and ROS damaging the healthy mitochondria. The dynamic version 1A of this (the Copasi computer code called model A.cps) produced a burst of both ROS and damaged mitochondria (Supplementary Information, Section A). The positive feedback loop causing this effect, involving mitochondrial damage and ROS production, is unavoidable in cells going through oxidative phosphorylation due to the proximity of FeS centers and molecular oxygen. To protect against ROS burst and five related dynamic instabilities, cells seem to have implemented five network mechanisms, corresponding to five “designs”, controlling five functionalities of the ROS network (Figs 1 and 2a).

### Antioxidant response and mitoptosis (Design 1): steady but not yet stable

ROS activate antioxidant responses as well as mitoptosis (i.e. mitophagy specifically of damaged mitochondria enhancing cell survival). Inclusion of these two processes (Design 1) enabled the ROS network model to attain the functionality of a steady state (Fig. 1a, Design 1, Fig. 2a–c and Supplementary Fig. B1.1, Model B1).

However, this steady state was found only for a precise balance between the rate constant at which ROS was produced and the rate constant at which ROS was removed. Increasing the rate constant of ROS production caused an indefinite increase in ROS concentration (Fig. 2c and Supplementary Fig. B1.1A). When decreasing the ROS generation rate constant, the concentrations of both ROS and damaged mitochondria dropped to 0 (Fig. 2c and Supplementary Fig. B1.1B). We see this precise balance of the corresponding rates as a regulation mode established by design 1: this design keeps the system steady.

If, after the drop of ROS and the concomitant drop in damaged mitochondria, the ROS generation was increased back to the initial level, the concentrations of both ROS and damaged mitochondria remained at 0, the “perfect” state. Moreover, when exposed to a sudden injection of ROS, the system maintained the new ROS concentration rather than returning homeostatically to the pre-existing steady state (Fig. 2b and Supplementary Fig. B1.1C). These results demonstrated that Design 1 and its regulation mode are of limited utility: although the resulting network was steady, it was both structurally and dynamically unstable and not homeostatic.

### Non-autocatalytic ROS and damage generation (Design 2A): stable but not yet robust

We first unfixed Antiox and p62 by adding production and consumption reactions, but this had no effect because they are not influenced by the rest of the network. Then assumption that ROS are only produced by damaged mitochondria is not realistic; there are other ROS-producing processes, such as oxidative folding in the endoplasmic reticulum and peroxisomal processes^2^. Thus, we added a reaction of basal ROS generation (Supplementary Fig. B2A.1, Design 2A, Model B2A1). Then, when ROS generation by damaged mitochondria was low because few mitochondria were left, ROS concentration was also low but not equal to 0, as described in details in the Model B2A.1 (Supplementary Fig. B2A.1).

When we decreased the total ROS generation in the new model, the concentration of ROS and of damaged mitochondria also decreased and reached a new non-zero steady state. When we returned ROS generation back to the initial level, the concentration of all species came back to the initial steady state (Fig. 2c and Supplementary Fig. B2A.1A). The system also became stable to perturbations in the initial concentration of ROS. When we first increased and then decreased the initial concentration of ROS, both the concentration of ROS and the level of healthy mitochondria went back to the initial steady state (Fig. 2b and Supplementary Fig. B2A.1B).

However, the stability of this system was limited. When the total ROS generation flux was increased by twofold, the concentrations of both ROS and of damaged mitochondria abruptly increased (Fig. 2c and Supplementary Fig. B2.A.1C). With Design 2A therefore the system is stable but not yet robustly so; it could well produce bursts of ROS.

An alternative way by which the system became partially stable emerged either when we took into account that mitochondria may get damaged also in the absence of ROS (Fig. 1b and Supplementary Information, Section B2A, Design 2A; Model B2.A2), or when both ROS production in the absence of damaged mitochondria and damage to mitochondria in the absence of ROS were implemented (Supplementary Information, Section B2A, Design 2A; Model B2.A3). The corresponding models B2.A2 (Supplementary Fig. B2A.1D–F) and B2.A3 (Supplementary Fig. B2A.1G–I) showed non-robust stability as did model B2.A1 (Supplementary Information, Section B2A).

One conclusion relevant for our further modeling approach is that zero concentrations of ROS and damaged mitochondria could be observed only for the case where we took into account neither basal ROS generation, nor basal mitochondrial damage (Model B1). Either of these two realistic processes forbids the “perfect state” with zero concentrations of both ROS and damaged mitochondria (Fig. 2c). In fact, we do not need to consider both basal ROS production and basal mitochondrial damage. On the one hand, basal production of ROS guarantees that there will be always a certain degree of mitochondrial damage, and, on the other hand, basal mitochondrial damage guarantees that there will be always a certain degree of ROS production. Thus, all models based on Design 2A would burst in terms of ROS and damaged mitochondria. Design 2A is insufficient for a robust behavior.

### Variable levels of healthy mitochondria (Design 2B): robust but not yet homeostatic

In the above models (Designs 1 and 2A) we considered the pool of healthy mitochondria to be unlimited. In reality, the cell has a limited capacity to synthesize new mitochondria and, thereby, to maintain the level of healthy mitochondria constant, independent of ROS damage. The sustained damage to mitochondria consequent to increased ROS should decrease the pool of healthy mitochondria. Therefore, we next assumed that the pool of healthy mitochondria is variable rather than constant. We also added a reaction of mitochondrial synthesis (Fig. 1b, Design 2B) at a constant flux, in order to maintain the possibility that the level of mitochondria reaches a steady state.

The new model, which included Design 2B, exhibited a similar response to the perturbation of ROS concentration (Fig. 2b and Supplementary Fig. B2B.1C, D, Model B2B), but did no longer explode in terms of ROS levels when we increased ROS generation. When we increased ROS synthesis twice, ROS concentration increased twice as well and reached a new steady state (Fig. 2c and Supplementary Fig. B2B.1E), while the concentration of healthy mitochondria was decreased around twofold (Supplementary Fig. B2B.1F). This effect makes Design 2B (as detailed in Supplementary Information, Section B2B) principally different from all previously discussed designs.

It is remarkable that doubling the rate constant for ROS synthesis caused a doubling of ROS concentration (Fig. 2c and Supplementary Fig. B2B.1E). Perhaps, paradoxically, this did not affect the level of damaged mitochondria, but it did halve the level of healthy mitochondria (Supplementary Fig. B2B.1F): it seems that variation in the level of healthy mitochondria is responsible for the regulation. The variation caused a temporary increase in the rate of mitophagy (Supplementary Fig. B2B.1I), thus causing a strong decrease in the total mitochondrial concentration. The decrease of healthy mitochondria caused a decrease in the source for damaged mitochondria, explaining why the concentration of damaged mitochondria returned to its initial level.

In Design 2B, we did not consider basal ROS synthesis or basal mitochondrial aging. When the ROS level was decreased, the level of healthy mitochondria was increased, which then secondarily again increased the rate of formation of damaged mitochondria. The latter phenomenon prevented the network from reaching the condition with zero concentration of damaged mitochondria and zero concentration of ROS (Fig. 2c and Supplementary Fig. B2B.1A, B, Model B2B).

Summarizing, comparative analysis of Designs 1, 2A, and 2B (Fig. 2c) revealed a function of Design 2: while basal ROS generation or basal mitochondrial damage or both (Design 2A) provides a dynamic, but not a structural robustness, the dynamic reduction of the abundance of mitochondria (Design 2B) ensures that both dynamic and structural robustness are achieved. Paradoxically, the dynamic reduction of the total mitochondrial pool may be essential to protect the cell. This is mitoptosis in action.

In Fig. 2d we plotted the steady-state concentration of ROS versus the fold change of the rate constant of ROS generation for Designs 2A and 2B. While in Design 2A a small increase of ROS generation resulted in a much higher increase of the ROS concentration, in Design 2B the twofold increase of ROS generation resulted in a doubling of the ROS concentration.

We computed the corresponding concentration control coefficient that shows how the increase of ROS generation affected the ROS concentration: $C_{{\mathrm {ROS}}\,{\mathrm{influx}}\,{\mathrm{rate}}}^{[{\mathrm {ROS}}]}$. We then also quantified the homeostatic adaptation, which we propose to quantify as

1 $$H\mathop { = }\limits^{{\mathrm{def}}} 1 - C_{{\mathrm {ROS}}\,{\mathrm {influx}}\,{\mathrm {rate}}}^{\left[ {\mathrm {ROS}} \right]} = 0.$$

That is, in Design 2B the concentration control coefficient was equal to 1 signifying H = 0, i.e. absence of homeostasis (Fig. 2d). In Design 2A the concentration control coefficient was higher than 1 signifying *H* < 0, i.e. negative (“anti-”) homeostasis.

### The Keap1–Nrf2 module enables homeostasis through negative feedback (Design 3): homeostatic yet potentially oscillatory

Designs 1, 2A, and 2B are based on a fixed rate constant of ROS consumption, which is determined by a fixed concentration of “Antioxidant Response” factors and a fixed first-order rate constant of mitophagy (with fixed concentrations of p62 and Parkin). In moving to Design 2, we had already added synthesis and degradation of p62 (as well as synthesis and removal of “Antioxidant response”). Synthesis and degradation of “Antioxidant response”, p62 and Parkin were balanced in such a way that steady-state concentrations of all species were identical to their concentrations in the previous model.

But now we increased the complexity by incorporating the Keap1–Nrf2 system, which modulates both mitophagy (by changing the p62 concentration) and an antioxidant response (by changing the concentration of antioxidant response species). When active, Keap1 binds to Nrf2, immobilizes Nrf2 in the cytoplasm, and marks (through ubiquitination) Nrf2 for degradation. Nrf2 is a transcription factor that actively shuttles between nucleus and cytoplasm^17^ and regulates the expression of p62 and genes responsible for an antioxidant response. Keap1 works as a ROS sensor that regulates the degradation and intracellular localization of Nrf2. ROS oxidizes cysteine residues in the Keap1 molecule; Keap1 changes its conformation and becomes inactive^30^. Thus, the higher is the concentration of ROS, the less active is Keap1 and, consequently, the higher is the concentration of Nrf2 in the nucleus, where it induces the expression of p62 (activation of mitophagy) and the antioxidant response. Antioxidant response removes ROS, and increased mitophagy removes damaged mitochondria, which produce ROS. Thus, a negative feedback is produced: the higher is the ROS concentration, the higher is the removal of ROS (Fig. 1c, Design 3 and Supplementary Information, Section B3).

Addition of the Keap1–Nrf2 module hereby adds a new emergent property to the system: upon an increase of ROS generation, the ROS concentration first increased but then decreased again, presumably due to the negative feedback loop which activated the antioxidant response and mitophagy and enabled homeostatic dynamic adaptation (Supplementary Fig. B3.1). With the combination of the positive (ROS inducing ROS via mitochondrial damage) and the negative (ROS reducing ROS via Nrf2–Keap1 signaling) feedback loops, the system exhibited transient oscillations of ROS and healthy mitochondria (Supplementary Fig. B3.1), as well as oscillations of activated Nrf2 (Supplementary Fig. B3.3) during the transition from one steady state to another in a certain range of the ROS generation rate constant.

The dynamic response of Design 2A showed an exponential increase of ROS concentration upon the increase of the ROS generation rate constant. Design 2B showed a linear increase of ROS concentration. In Design 3, the curve showing the variation of ROS steady-state concentration with the variation of ROS generation rate was less than linear, and progressively less sharp (Fig. 2d). The corresponding control coefficient was $C_{{\mathrm {ROS}}\,{\mathrm{influx}}\,{\mathrm{rate}}}^{[{\mathrm {ROS}}]} \approx 0.64.$ The reduction of this control coefficient to below 1 signifies homeostatic adaptation, which was equal to

2 $$H= 1 - C_{{\mathrm {ROS}}\,{\mathrm {influx}}\,{\mathrm {rate}}}^{\left[ {{\mathrm {ROS}}} \right]} = 0.37.$$

This number corresponds to the tangent to the curve of the ROS concentration versus the change of ROS generation, at the point where ROS generation was not changed yet (fold change of ROS generation was equal to 1). At the zone where ROS generation is substantially decreased, the curve was steeper. Thus, homeostatic adaptation was lower, and in the zone where ROS generation was high, the homeostatic adaptation was higher.

This can be identified as Design 3: The Nrf2–Keap1 feedback provides homeostatic adaptation. As a consequence, xenobiotics (like coffee, which besides caffeine contains a complex mixture of various chemical substances) interfering with the Keap1-Nrf2 system may affect the response to oxidative stress by affecting homeostasis, as shown below.

However, the negative feedback loop of Design 3 introduces a problem. Designs 2A and 2B were robust against the perturbations of ROS concentration. Upon injection of ROS, the ROS, as well as all other species returned to their initial steady-state levels (Fig. 2b). This is not anymore the case for Design 3. When ROS concentration is increased, e.g. 10%, the network started oscillating (Fig. 2e and Supplementary Fig. B3.2). During oscillations, the concentration of healthy mitochondria swept below a viability threshold that we took corresponding to 20% of the initial concentration of healthy mitochondria (Fig. 2d and Supplementary Fig. B3.2B), or for other parameter values the level of mitochondria grew to non-physiological levels. We imagine that a reduction of ATP formation capacity by 80% should be lethal for the cells, and an excessive level of healthy mitochondria may deplete cells of essential resources.

### Mitochondrial repair via NFκB signaling (Design 4): robustness against the ROS injections yet fragility vis-à-vis repeated challenges

As shown above, mitophagy of damaged mitochondria averts excessive ROS generation, thus preventing the cell from ROS-induced damage that might lead to apoptosis^11^. Mitophagy, however, leads to loss of mitochondria, and mitochondria play a dominant role in the free energy transduction. Loss of ATP synthesis flux is expected to lead to a drop in ATP/ADP ratio and thereby compromise ATP requiring processes. Should the maintenance metabolism be compromised, the loss could lead to necrosis. There is a physiological mechanism where, instead of being degraded in mitophagy, damaged mitochondria are repaired^77–79^. We incorporated this mechanism into the next design (Fig. 1d, Design 4 and Supplementary Information, Section B4) where NFκB signaling activates mitochondrial recovery via activation of the expression of Bclxl^80^.

With this Design 4 (Model B4) the system became robust against the injection of ROS (Fig. 2e and Supplementary Fig. B4.1). Immediately upon the increase in ROS concentration, the latter returned to the initial value (Supplementary Fig. B4.1A). Healthy mitochondria followed these perturbations and did not sweep below a hypothetical viability at 20% of the initial concentration of healthy mitochondria (Supplementary Fig. B4.1B). This may well identify the function of Design 4: addition of mitochondrial recovery via NFκB signaling provides dynamic robustness to the homeostatic design.

NFκB signaling also helps to resist high rates of ROS generation. When ROS generation was increased 15-fold and NFκB signaling was not activated, the concentration of healthy mitochondria dropped below a hypothetical viability line (Supplementary Fig. B4.2B). However, healthy mitochondria were saved if NFκB signaling was activated 15-fold by 6 h (Supplementary Fig. B4.2C), or even 3 h (Supplementary Fig. B4.2D) prior to the increase of ROS generation. When NFκB was increased, the damaged mitochondria started to recover into healthy mitochondria. The concentration of damaged mitochondria decreased. Thus, the ROS concentration decreased as well and the concentration of healthy mitochondria started increasing. The increase of ROS synthesis stopped the increase of the concentration of healthy mitochondria. ROS concentration then increased again, and healthy mitochondria dropped again. But, overall, the concentration of healthy mitochondria did not cross a viability line.

At this stage, we could conclude that activation of NFκB signaling is advantageous for mitochondrial functionalities. The plot of steady-state concentration of Healthy Mitochondria versus ROS showed that for higher NFκB signaling, and for the same level of ROS production, a higher level of Healthy Mitochondria could be maintained (Supplementary Fig. B4.2E).

However, there is also a potential danger of the accumulation of healthy mitochondria in Design 4 (Supplementary Fig. B4.3): when healthy mitochondria accumulate due to high NFκB activity and if ROS generation is suddenly increased, accumulated healthy mitochondria could serve as a substrate for the production of damaged mitochondria. Because the concentration of substrate is high, there is also a high rate of mitochondrial damage and ROS generation. This triggers a positive feedback loop: ROS damage mitochondria and damaged mitochondria produce more ROS. When damaged mitochondria accumulate faster than they can be neutralized by mitophagy, sharp peaks of damaged mitochondria and of ROS are observed. A vicious cycle fed by the high initial concentration of healthy mitochondria then leads to a collapse of the whole system. To avoid this catastrophe, the rate of mitochondrial recovery should adapt to ROS concentrations proportionately.

### DJ-1 as a ROS sensor that coordinates Nrf2 and NFκB signaling (Design 5): robustness against dynamic repeated challenges

DJ-1 protein is a ROS sensor that coordinates NFκB signaling with Nrf2-Keap1 signaling. When oxidized by ROS, the conformation of DJ-1 is changed. When DJ-1 becomes active it activates the rate of mitochondrial recovery via NFκB in a ROS-dependent manner. In parallel, DJ-1 amplifies ROS-induced Nrf2–Keap1 signaling, thus activating both mitophagy and antioxidant response. To examine the role of DJ-1, we added the DJ-1 module to Design 4 thereby obtaining Design 5 (Fig. 1e).

We studied 3 versions of Design 5 (Supplementary Information, Section B5): (i) Design 5.1 (Model B5.1), where DJ1 regulated NFκB signaling only, (ii) Design 5.2 (Model B5.2), where DJ1 regulated Nrf2–Keap1 signaling only, and (iii) Design 5.3 (Model B5.3), where DJ1 regulated both NFκB and Nrf2–Keap1 signaling.

When healthy mitochondria accumulated due to high NFκB activity and low ROS generation, and ROS generation was suddenly increased, due to a second pulse of ROS, at the time point when the concentration of healthy mitochondria was near its peak value, in both Designs 5.1 (Supplementary Fig. B5.1) and 5.2 (Supplementary Fig. B5.2), the concentration of healthy mitochondria transiently swept below a hypothetical viability line (at 20% of the initial concentration of healthy mitochondria), but quickly recovered back to its initial level. This demonstrated the limited robustness of the model with respect to the second pulse of ROS when NFκB or Nrf2–Keap1 signaling pathways are already active. When similar perturbations were applied to Design 5.3 (Supplementary Fig. B5.3), the concentration of healthy mitochondria did not sweep below the viability line revealing that Design 5.3 is robust against the second pulse of ROS and that simultaneous regulation of both NFκB and Nrf2 signaling pathways should be advantageous under such dynamic conditions.

To understand whether robustness is due to summation of two regulatory mechanisms, we increased ROS generation in Design 5.3 not only 15, but also 30-fold, i.e. twice higher than the cases when each mechanism worked alone. Still the concentration of healthy mitochondria did not sweep below a viability line (Supplementary Fig. B5.4). In the next computational experiment (Design 5 with regulation of both NFκB and Nrf2 signaling), the sensitivity of DJ1 to ROS was reduced twofold (Model B5.3.2). In spite of the reduced DJ1 activity (Supplementary Fig. B5.5A), Design 5.3.2 exhibited a behavior similar to Design 5.3, and was still the most advantageous when comparing with Design 4, Design 5.1 and design D5.2 (Supplementary Fig. B5.5B).

The final Design 5 with both NFκB and Nrf2 regulated by DJ1 exhibited very strong homeostasis (Fig. 2d and Supplementary Fig. B5.6). The corresponding control coefficient was $C_{{\mathrm {ROS}}\,{\mathrm {influx}}\,{\mathrm {rate}}}^{[{\mathrm {ROS}}]} \approx 0.15$, which signifies strong homeostatic adaptation, i.e.,

3 $$H = 1 - C_{{\mathrm{ROS}}\,{\mathrm{influx}}\,{\mathrm{rate}}}^{\left[ {{\mathrm{ROS}}} \right]} = 0.85.$$

Overall, we conclude that Design 5 provides robustness against a second injection of ROS and strong homeostasis. This should be due to DJ-1 and synergistic coordination of Keap1–Nrf2 and NFκB signaling.

Since DJ-1 coordinates mitochondrial recovery and amplification of Nrf2 signaling, and helps to bring dynamic homeostasis close to perfect adaptation, mutations in DJ-1 might lead to PD in cases where the network is challenged by large perturbations. When DJ-1 is present, the model seems perfect. However, it may still fail when challenged with additional disease-related biological “detail”, such as ROS-dependent polymerization of α-synuclein.

### Remaining liability: limited capacity to deal with α-synuclein polymerization

In the previous designs, ROS activated p62 expression required for mitophagy. This process involved two negative feedback loops: (i) via Keap1–Nrf2 signaling and (ii) via DJ1 signaling. However, ROS might also reduce mitophagy via α-synuclein polymerization. Increased ROS concentration induces α-synuclein polymerization. α-Synuclein aggregates sequester p62, and the lower p62 concentration could then be responsible for a decline in mitophagy.

We added the α-synuclein module to Design 5 (Model B5) thereby obtaining Model C (described in detail in Supplementary Fig. C.1). When α-synuclein was absent, Model C was at a steady state. With the addition of a constant source of α-synuclein at low concentration, oxidation of α-synuclein by ROS caused the formation of α-synuclein aggregates in our model, which sequestered p62 and reduced mitophagy. Due to the reduction of mitophagy, this mechanism caused an increase of the concentration of healthy mitochondria in silico (Supplementary Fig. C.2A). This hypothetical scenario could be attractive evolutionarily because it favors the increase of ATP production at the cost of somewhat elevated concentrations of ROS and damaged mitochondria.

When the concentration of α-synuclein was increased, the system did not reach a feasible steady state (Supplementary Fig. C.2B). The concentration of damaged mitochondria started to grow constantly and the system burst, demonstrating the potential danger of ROS-induced α-synuclein polymerization.

We also checked the robustness of model C to a 10-fold increase of the ROS generation rate constant in the absence or presence of a source of α-synuclein at low concentration. Without α-synuclein, the system exhibited strong homeostasis (Supplementary Fig. C.3). When a constant source of α-synuclein was added at low concentration, and the ROS generation rate constant had not yet been increased, the system attained a new steady state (Supplementary Fig. C.4). However, once the ROS generation rate constant was increased in the new model that was already in a steady state, the steady state was no longer reachable (Supplementary Fig. C.5).

To summarize, our in silico cell may tolerate (and perhaps even profit from) the reduction of mitophagy due to ROS-induced α-synuclein polymerization, in the case of low ROS concentrations and low concentration of α-synuclein. However, such ROS-induced α-synuclein polymerization decreases the robustness against oxidative stress.

It is remarkable that in our simulations (Supplementary Information, Section C) healthy mitochondria were not lost. This was probably because there was no mechanism in the model to stop mitochondrial synthesis even when damaged mitochondria and ROS grew to infinity. We expect that in live cells no more mitochondria could be produced with the accumulation of damage. Then the rate at which mitochondria are damaged should ultimately decrease in parallel to the decrease in the level of healthy mitochondria. This hypothesis will be examined in a more detailed and complete model in the following sections.

### More detailed and complete ROS-management model, and validation by in vitro experiments

**Fig. 3:**
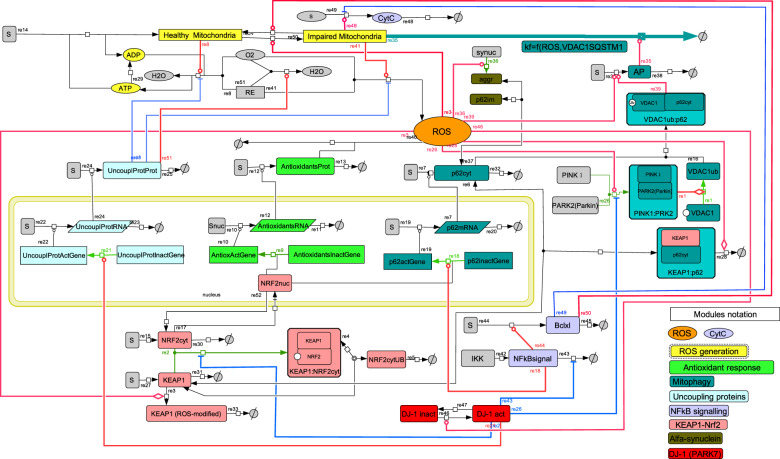
Network diagram of the comprehensive model (“Model D”) of ROS management. The resolution of model C (Supplementary Fig. C.1) was increased, i.e., the nucleus and cytoplasm compartments, an mRNA layer for several proteins, and several additional species and interactions were added as follows. *ATP module*: A constant source of reductive equivalents (RE) in the form of reduced NAD^+^ (i.e. NADH+H^+^), and a constant source of O_2_ drive three reactions: (i) in the reaction catalyzed by healthy mitochondria (re8), reduction of O_2_ to H_2_O is coupled to phosphorylation of ADP into ATP; (ii) in the reaction catalyzed by impaired mitochondria (re41), reductive equivalents are only used as a source of incompletely reduced oxygen species (ROS); (iii) in the reaction activated by uncoupling proteins (re51), O_2_ is reduced to H_2_O without phosphorylation of ADP into ATP. ATP is dephosphorylated back to ADP in reaction re29, which represents the overall net reaction of all ATP consuming reactions. *Uncoupling proteins*: The expression of uncoupling proteins is activated by active DJ-1 in the “bipartite” irreversible reaction (re21). Genes coding uncoupling proteins are present in active (transcribed) and inactive (silent) forms. The higher is the concentration of DJ-1, the higher is the transcription of uncoupling proteins (re22). In turn, the higher is the concentration of uncoupling proteins mRNA, the higher is the rate of its translation into uncoupling proteins (re24). Uncoupling proteins inhibit the production of both ATP (re8) and ROS (re41) and activate the uncoupled respiration (re51). Since the concentrations of all its substrates (O_2_ and reductive equivalents) and products (H_2_O) are fixed, the reaction of respiration was omitted in the COPASI version of the models. A strategy similar to the modeling of “uncoupling proteins” expression was used for the modeling of antioxidant response and p62, for which active (transcribed) and inactive (silent) forms of genes were considered too. Activation of transcription would mean an activation of the “bipartite” irreversible transition of a silent gene into its transcribed gene (re9 and re18). Binding of the active (localized in the nucleus) fraction of Nrf2 transcription factor, which shuttles between the nucleus and cytoplasm (re17 and re52), facilitates the transition of a silent gene to its actively transcribed counterpart. When the gene is active, it catalyzes the transcription (production of mRNA in re10 and re20). When the concentration of mRNA increases, translation is activated (the production of proteins in re7 and re12). *More detailed mitophagy mechanism*: Pink1 binds to parkin in a “bipartite” irreversible reaction (re26) and facilitates ubiquitination of VDAC1 in another “bipartite” irreversible reaction (re1). The latter interacts with p62 (re16) to form a complex that, like ROS, facilitates the formation of apoptotic machinery (re39). This apoptotic machinery (called AP) catalyzes the reaction re35 in which impaired mitochondria are degraded. *Cyt C and NFκB*: Impaired mitochondria release Cyt *C* (re49). Apart from activating mitochondrial recovery (re50), Bclxl inhibits Cyt *C* release. When Cyt *C* exceeds a threshold, it induces cell death (not shown on diagram). *Keap1 module*: Keap1 transiently binds and then ubiquitinates and thereby facilitates degradation of both Nrf2 (reaction chain re2, re4, and re5) and p62 (re6 and re28; p62 is not released back; it is not a catalytic factor but a co-substrate). In these diagrams SBGN notation was used, i.e. –o for stimulation, -| for inhibition and – for co-reaction. ↔ refers to reaction, which can be reversible [black double headed arrows], → irreversible [black arrows], “bipartite” irreversible [green arrows] (this is specified in the Copasi files). “Bipartite” irreversible refers to any case where a process has a forward and a reverse reaction that are not each other’s microscopic reversal, the one being affected by a third agent while the other is not. Reaction numbers are positioned at the origins of reaction arrows. The species with constant concentration (e.g. a constant source of substrate or a constant sink of product) are shown in gray color.

Up to this point, we have made models that should highlight the functionality of a design and were therefore kept simple. Now we will consider a much larger model that should represent reality more comprehensively (Fig. 3). All features of the five designs and the α-synuclein module (Supplementary Information, Section C) were maintained, but nucleus and cytoplasm compartmentalization, an mRNA layer for several proteins, and several additional species and interactions were added (Fig. 3). In particular, in the new ATP module, reductive equivalents present at fixed levels in NADH + H^+^ provided electrons to the electron transport chain for the reduction of molecular oxygen either to water or to incompletely reduced oxygen (ROS). The former process consisted of a part that was coupled to the phosphorylation of ADP, as well as a part that was uncoupled of that phosphorylation. As we did not measure oxygen consumption and the oxygen levels were modeled as fixed, the latter process is not reported on.

Our detailed mitophagy mechanism includes Pink1^81,82^ which activates Parkin E3^83,84^ and facilitates the ubiquitination of VDAC1. The latter interacts with p62 and facilitates the formation of the apoptotic machinery. Besides activating Nrf2 and NFκB, DJ1 activates the expression of uncoupling proteins, which reduce mitochondrial transmembrane potential and inhibit the production of both ATP and ROS. This process was here modeled by the uncoupling proteins inhibiting ATP production, stimulating uncoupled respiration and inhibiting ROS production. More details of NFκB signaling have been incorporated^85,86^. Moreover, we have taken into account that impaired mitochondria release cytochrome *c* (Cyt *C*). In addition to activating mitochondrial recovery, Bclxl inhibits Cyt *C* release. When Cyt *C* release exceeds a certain threshold, it induces cell death. In the new model, Keap1 ubiquitinates and thus facilitates the degradation of both Nrf2 and p62. For p62, antioxidant response, and uncoupling proteins, active (transcribed) and inactive (silent) forms of the corresponding genes were considered, giving rise to the corresponding mRNAs and proteins (Fig. 3). The resulting comprehensive model was called Model D.

### Validation of the comprehensive model by in vitro experiments

**Fig. 4:**
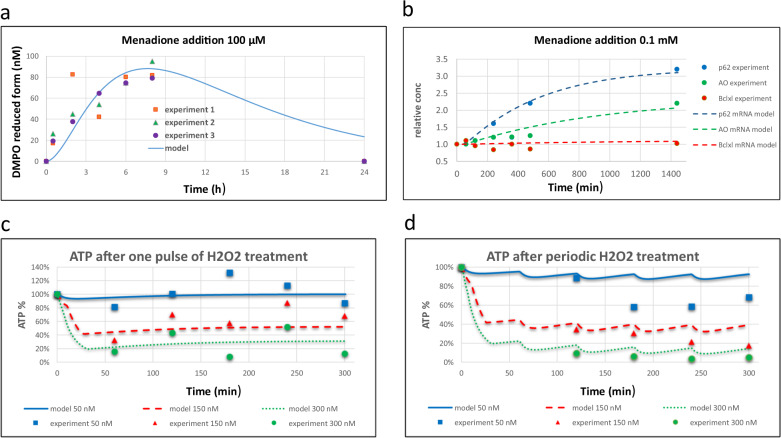
Validation of the comprehensive model D in terms of response to menadione and hydrogen peroxide. **a**, **b** Validation in terms of response to menadione. These data were taken from the study reported on by Deferme et al.^121^. **a** Change in the concentration of reduced DMPO (used as a sensor of ROS) upon addition at time zero of menadione (100 μM) in model D as compared to the response of HepG2 cells, starting from 0 at the steady state before the addition of menadione. **b** Fold change (relative to initial) in concentrations of mRNAs upon an addition at time zero of menadione (100 μM) in model D as compared to the response of HepG2 cells, starting from 1 at the steady state before the addition of menadione. A time delay of 35 min was taken into account in the modeling. **c**, **d** Changes in relative concentrations of ATP upon addition at time zero of H_2_O_2_ (50, 150, or 300 μM) to PC12 cells. **c** The concentration of ATP (% of its initial value) after one single addition to PC12 cells (at *t* = 0) of H_2_O_2_: 50 μM (blue), 150 μM (red), or 300 μM (green). This single treatment was either a continuous or a pulse treatment. In the pulse treatment, H_2_O_2_ was washed away after 30 min by replacing the medium. In the continuous treatment, no such action was taken. Separate experiments (Supplementary Fig. D.2A) showed that peroxide degrades much faster than the time before washing. Thus, we did not differentiate between pulse and continued treatment during model fitting. **d** The concentration of ATP (% of its initial value) during periodic treatment with different concentrations of peroxide. H_2_O_2_ 50 μM (blue), 150 μM (red), or 300 μM (green) was added once per hour throughout the experiment, starting at *t* = 0 h. In all cases model D (corresponding to Fig. 3) was used to calculate model predictions (see also Methods and Supplementary Information, Section D). The perturbations by menadione and H_2_O_2_ were modeled as follows. Both menadione and H_2_O_2_ are transported to the cell in reversible reactions, and degraded both in the extracellular media and in the cell. Their rate of degradation in the cell is higher than in the extracellular media. In the cell, menadione induced ROS generation (re41 in Fig. 3), which corresponds to the menadione-induced inhibition of mitochondrial complex I, enhancing electrons leakage for ROS generation. H_2_O_2_ catalyzes the production of other species, so-called “damage”, which correspond to ^.^OH radical formation and the accumulation of cellular damage done by the hydroxyl radical (such as DNA mutation, lipids oxidation, etc.). “Damage” is also removed (damage reparation). Similar to menadione, “damage” causes an increase of ROS generation (re41 in Fig. 3). When replacing this menadione module (**a**, **b**) with an H_2_O_2_ module (**c**, **d**), all parameters in the model were kept the same, except for those relating to ROS induction by menadione or H_2_O_2_, or the transport and degradation of menadione and H_2_O_2_. The model is publicly available at FAIRDOMHub^123^. The calibrated version is available at 10.15490/FAIRDOMHUB.1.MODEL.643.1. The model can be simulated online for FAIRDOMHub registered users, or the COPASI version of the model can be downloaded.

Model D was then fitted to two independent data sets stemming from in vitro experiments performed in different labs on different cell types (Supplementary Information, Section D). First, we fitted the comprehensive model to the fold change in the relative concentrations of ROS and mRNAs (Fig. 4a, b and Supplementary Table D.T1) upon addition of menadione (100 μM) to HepG2 cells, starting at 1 h of incubation with menadione. Menadione does not induce ROS immediately. A time delay of 35 min was therefore taken into account in the model simulation. The effect of menadione was modeled by assuming that menadione is transported into the cell and degraded, with a higher rate of degradation inside the cell than in the extracellular media. In the cell, menadione then induces ROS generation (re41 in Fig. 3). This corresponds to the menadione-induced inhibition of mitochondrial complex I, enhancing electron leakage towards ROS generation.

Then we studied another mechanism of ROS induction, through addition of H_2_O_2_, which was assumed to produce ^.^OH radicals damaging the electron transport chain. An additional species called “damage” was produced and removed (damage repair). Damage caused the increase of ROS generation in re41 (Fig. 3). When replacing the menadione module with an H_2_O_2_ module, all parameters in the model were kept the same. Although only the parameters of ROS induction by H_2_O_2_ were held adjustable, the model was capable to reproduce the experimentally observed data of the time-dependent ATP change (Fig. 4c, d and Supplementary Table D.T2). In these experiments, PC12 cells were exposed to H_2_O_2_ (50, 150, or 300 μM) in either one single treatment (Fig. 4c and Supplementary Fig. D.2A, B, E) where H_2_O_2_ was added only once at *t* = 0, or periodically (Fig. 4d and Supplementary Fig. D.2C, D, F) where H_2_O_2_ was added once every hour. For the highest H_2_O_2_ levels we no longer observed ATP experimentally. In such cases, the cells were dying or dead, which is what our model does not deal with other than predicting low ATP concentrations.

The fact that our comprehensive model D satisfied experimental data from both menadione and H_2_O_2_ titrations should provide some confidence in the model. On the other hand, a model of this complexity will need much more experimental validation and calibration in the future. Nevertheless, we decided to examine whether this somewhat validated model might resolve some important issues around aging and (Parkinson’s) disease.

### The regulation modes in the comprehensive model

As described in Supplementary Information E and F, we checked how the five designs identified above with the use of simplified models might continue to work in the detailed model. Memory and preconditioning persisted (Supplementary Information, Section E). The first three designs worked in a straightforward way: without mitophagy (Design 1) the system did not reach any steady state (Supplementary Fig. F.1), without limiting the mitochondrial synthesis (Design 2) the structural robustness was lost (Supplementary Fig. F.2), and without Keap1–Nrf2 signaling (Design 3) the homeostasis was substantially decreased (Supplementary Fig. F.3). Homeostasis was not lost completely, because other feedback mechanisms, such as NFκB and DJ-1 signaling, were still working in the system.

**Fig. 5:**
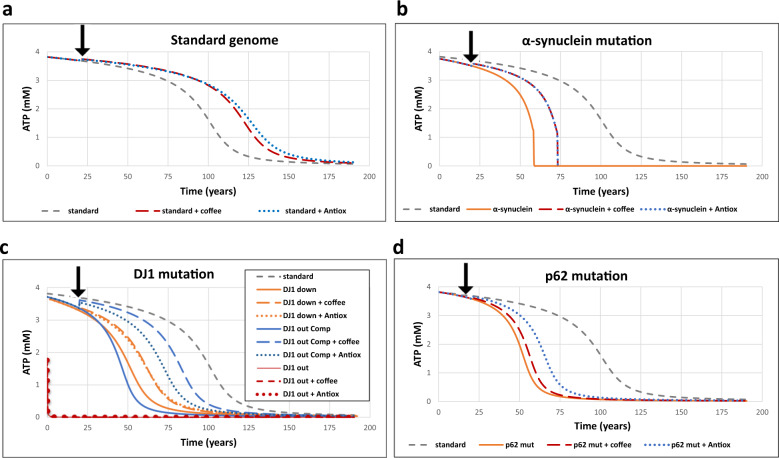
Personalized aging and medicine. The detailed model D (Fig. 3) was used for the simulations. Species representing ROS-induced damage accumulated in the model, which enhanced ROS production by impaired mitochondria. The treatment with coffee started at 20 years and was simulated by the 1.1-fold activation of Nrf2 nuclear import. The treatment by antioxidant started at 20 years as well and was simulated as the 1.2-fold activation of the synthesis of antioxidant proteins. Aging (represented as the decline of ATP concentration thought to represent generalized failure of energetics) was simulated for four scenarios (**a**–**d**). **a** Simulated ATP(*t*) in a healthy cell without any treatment (dashed gray line), and when treated with antioxidants, starting at 20 years as well, and simulated as the activation of antioxidant proteins synthesis (1.2-fold, dotted blue line), or with caffeine, started at 20 years and simulated by activation of the Nrf2 nuclear import (1.1-fold, dashed red line). **b** Aging when α-synuclein source and thereby the rate constant of α-synuclein “aggregates” formation (re36) is increased twofold without any treatment (solid orange line), or when being treated (like treatments in **a**) with either antioxidants (dotted blue line) or coffee (dashed red line), is compared with the standard aging of a healthy cell (dashed gray line). **c** Aging in the presence of DJ-1 mutations, without any treatment (solid lines), or when being treated (like treatments in **a**) with either antioxidants (dotted lines) or coffee (dashed lines) is compared with the standard aging in a healthy cell (dashed gray line). Three mutated sub-versions were modeled: (i) DJ-1 mutation (“DJ1 down”: orange lines), where DJ-1 activity is decreased twofold, (ii) DJ-1 mutation (“DJ1 out”: red lines), where the concentration of total DJ-1 protein is kept at almost 0, and (iii) DJ1 mutation compensated with the increased activity of Keap1–Nrf2 signaling, modeled by a fourfold increase of the rate constant of Nrf2 production (“DJ-1 out Comp”: blue lines). Dashed lines refer to the corresponding cases treated as in **c**. **d** Aging in the presence of a p62 mutation in which p62 mRNA level is fixed and is not regulated by ROS, without any treatment (solid orange line), and when being treated (like treatments in **a**) with either antioxidants (dotted blue line) or coffee (dashed red line). All cases are compared with the standard aging in a healthy cell (dashed gray line). The mutation of α-synuclein was simulated by increasing the fixed concentration of α-synuclein twice. The mutation of “DJ-1 down”, which means “DJ-1 knockdown”, was simulated by decreasing the total DJ-1 concentration twice. The mutation of “DJ-1 out”, which refers to a DJ-1 knockout, was simulated by decreasing the total DJ-1 concentration 500 times. The mutation of “DJ-1 out com”, meaning a DJ-1 knockout compensated by Nrf2, was simulated by taking the model with the DJ-1 knockout mutation and increasing the rate constant of NR2 synthesis fourfold. The mutation “p62 mut” was simulated by fixing the concentration of p62 mRNA at the initial steady-state value; thus, the transcription p62 was no longer regulated by Nrf2.

The interpretation of Design 4 (Supplementary Fig. F.4) and Design 5 (Supplementary Fig. F.5) in the detailed model was less direct at first sight. However, when considering these designs in the context of ROS-related aging, the role of both NFκB (Design 4) and DJ-1 (Design 5) signaling pathways became obvious. When NFκB (Supplementary Fig. G.1) or DJ-1 (Fig. 5c) signaling was compromised, an earlier ROS-induced aging was observed.

The role of α-synuclein in the detailed model (Supplementary Fig. H.1) was slightly different from the role discovered earlier in the simplified model C (Supplementary Information, Section C). The negative role of α-synuclein (increasing ROS concentration) persisted. However, the positive role (increasing the concentration of healthy mitochondria) was no longer observed. With the use of the detailed model, we also detected a possible role of increased concentration of misfolded α-synuclein in the development of early ROS-induced aging (Fig. 5b).

Since the abovementioned network designs might be compromised due to various mutations, for example mutations leading to reduced NFκB or DJ-1 signaling or to increased formation of α-synuclein aggregates, we used the comprehensive model to simulate age-related diseases, such as PD.

### Multiple processes that determine aging and network-based therapy design

An advantage of a comprehensive mathematical model of a disease detailed down to the level of molecular components is that it can be used for computational network-based identification of drug targets^87^. The effect of the interference with a molecular process on a flux can be quantified by a so-called flux-control coefficient; processes with the higher flux-control coefficients are the better drug targets if a flux (such as the growth rate of a parasite) is the culprit. In this case, the interest is in postponing the age at which aging accelerates, which we shall here take as the time (*T*_0.5_) at which ATP has declined to 50%. We here define an aging-time-control coefficient with respect to any molecular process essentially as the percentage increase in *T*_0.5_ upon a 1% activation of that molecular process (see Supplementary Information, Section I for a more precise definition). Table 1 identifies the processes with a more than proportional control on aging-time. Clearly, the control is not in one single process; it is distributed. Where the flux-control in metabolic pathways tends to be between 0 and 1, we here find an aging-time-control coefficient ranging from −2.6 to 1.7. The −2.6 and 1.7 mean that accelerated aging could be postponed by 50% by reducing mitochondrial synthesis rate by a mere 20%, or by increasing the rate of p62 synthesis by 30%. Physical exercise in terms of doubling cellular ATP consumption would have to be increased by 50% in order to produce the same effect. Targets with positive control coefficients should be activated, but this is often more difficult than inhibiting a process. Consequently, processes with the strongest negative control coefficients might be the preferred drug targets. There are also 33 processes with time-control coefficients smaller than 1 in absolute magnitude (Supplementary Table J). They should not be envisaged as drug targets; in this way, our analysis and model may help direct research investments away from unsuccessful leads. The control coefficients are high as compared to the numbers (such as 0.2) usually found for the control of metabolic fluxes or concentrations^88^. This is due to the time warp to be discussed below.

**Table 1:** Best network-based target processes for drugs to retard aging.

Process	Control on time when ATP = 0.5
Synthesis of mitochondria	−2.6
KEAP1 synthesis	−1.8
p62RNA-ase	−1.7
p62 synthesis	1.7
p62 transcription	1.7
KEAP1 damage	1.5
Degradation of damaged mitochondria	1.5
Synthesis of apoptotic machinery	1.5
Synthesis of antioxidant proteins	1.5
Transcription of antioxidant genes	1.5
ROS removal	1.5
Degradation of apoptotic machinery	−1.5
Degradation of antioxidant mRNA	−1.5
ROS synthesis	−1.5
Degradation of antioxidant protein	−1.5
Nuclear export of NRF2	−1.4
Repair of KEAP1	−1.4
NRF2 synthesis	1.4
NRF2 nuclear import	1.4
Degradation of damaged KEAP1	1.4
Binding of KEAP1 to NRF2	−1.2
Oxidative phosphorylation	−1.1
Damaging of mitochondria by ROS	1.1
Cellular ATP consumption	1.1
Generation of damaging factors	−1.0

Aging-time-control coefficients calculated for the comprehensive model by increasing the activity of each of the processes mentioned in the first column by 0.1%, computing the percent change in time at which ATP was 50% of maximal and multiplying the result by 10. Only processes with aging-time-control coefficients in excess of 1 in the absolute value are shown here (other processes and the actual numerical procedure used are shown in the Supplementary Information I, J and O). Positive and negative values mean that aging is delayed or accelerated, respectively, by activation of the process.

### Model calibration

The comprehensive model (model D) was then fine-tuned (refitted) so as to predict a steady-state concentration of Cyt *C* (6.6 μM)^89^, of mitochondria (2.1 nM) (using a cell volume of 2 pL^90^, an ATP/O ratio of 2.5, and a mitochondrial turnover time of some 10 days^91^). As shown in the Supplementary Information (Section K), and on the basis of a Y_ATP_ of 57 mmol ATP per gram dry weight^92^, we estimated that one mitochondrion should require approximately 0.77 billion ATP molecules to energize its synthesis and put this stoichiometry in place in the model. As a simplification, we assumed that this ATP would come at the cost of the maintenance reaction, such that total ATP consumption by the cell would remain the same. As shown in the Supplementary Information, the steady-state concentrations of ATP, ADP, oxygen, mitochondria, cytochrome *c*, and redox equivalents (NADH) were realistic. The resulting calibrated comprehensive model of ROS management was short named as ‘model D’. A complete listing of its parameters is given in Supplementary Information (Section L).

### Re-validation of the now calibrated comprehensive model by in vitro experiments

Model D was validated against the same experimental data as were used for the validation of model D, and turned out to be equally valid. This is understandable, as we calibrated the model in a conservative way (see Supplementary Information, Section K) and we used the interaction parameters with menadione and hydrogen peroxide to fit the new, calibrated model.

### Implications for aging, memory, and PD

We asked whether our comprehensive and partly validated ROS-management model could reproduce in silico, and thereby help us to understand, some intriguing observations reported in the literature. These include a time span and dynamics of aging much longer than the life times of the molecules engaged, the variegated effects of “Nature” (i.e. of various individual genes), the effects of “Nurture” (i.e. of diet, such as coffee and antioxidants), as well as personalized effects of diet–gene combinations.

### Aging’s time warp reproduced

Healthy aging in the absence of apparent diseases, which is what we shall discuss here, is a remarkable temporal phenomenon^93^: Although time proceeds steadily, there is little physical deterioration of health until high age is reached (typically 90 years). Then physical decline starts, slowly at first, but accelerating with time until a rather sudden breakdown occurs^94,95^. We refer to this peculiar time dependence as “aging’s time warp” because of the apparent, progressive contraction of the time dimension, leading to the relatively sudden jump from “healthy” to “frail”.

The challenge for the network of being resistant towards this aging differs from the five dynamic robustness challenges that we studied above. We therefore wondered whether the five corresponding network mechanisms, or the genes involved in them, impact aging, and indeed, whether our comprehensive model of ROS management produces such a time warp rather than just a gradual decline of performance. Under some conditions, such as a steady decrease in the temperature of water, phase transitions also exhibit such time warps.

We focused on somatic mutations caused by ROS on top of a constant drizzle of such mutations, damaging the genes encoding the mitochondrial respiratory chain, thereby leading to a gradually increasing production of ROS by damaged mitochondria. When incorporating this mechanism into the calibrated comprehensive model (model D) and simulating this model’s behavior at the timescale of years, we observed that at first there was nothing like aging: ATP was stable. But then, a decline (at first gradual, but subsequently sharp) of the ATP concentration, emerged around the age of 90. The relatively sudden change from healthy to pathological ATP levels was similar to the time warp of aging (dashed line in Fig. 5a). This time warp has the implication that although the system is never quite in a steady state, it is in a quasi-steady state, this is more so at ages before 75 years than around 90 years. At low ATP levels, which might cause death, the system would lose steady state, but this is not represented in our model. We conclude that our comprehensive model appears to predict a ROS-related aging that has this time-warp signature.

Aging comes with more than an initially slight reduction in ATP levels, but also with reduced activities of many processes involved in ROS management, such as decreased activities of catalase, glutathione, and SOD^41–44^, and of many other processes, most of which are not in our model. We chose ATP as a monitor of aging, as a reduced ATP/ADP ratio should indeed compromise most cellular quality control systems. Aging is pleiotropic however and due to all processes faltering more or less at the same time.

### Preconditioning and mitohormesis

Why is the effect of time so sudden after a long period of ineffectiveness? In other words, why did the drizzle of mutations remain ineffective for the first 50 years? Part of the answer may reside in some of the five regulation modes (designs) identified above. In further computations, we found a phenomenon related to the five functional modes that might explain the time warp: preconditioning, also called mitohormesis^96^. When we exposed the comprehensive model (model D) to consecutive stress events (i.e. the increase of the ROS generation rate constant) we discovered that prior stress stimuli enabled the system to deal better with a subsequent such stimulus (Supplementary Fig. E.1A). This preconditioning was observed on the timescale of hours and was explained in terms of “memory” of the antioxidant response that was maintained at an elevated level after stress stimuli (Supplementary Fig. E.1B). One should expect that a long series of stress events corresponding to the drizzle of mutations might also lead to increased preconditioning by ROS, thereby preventing any sign of aging. We tested this by increasing the impact of ROS on mitoptosis by elevating the rate constant of synthesis of the mitochondrial autophagy machinery by 50%. Indeed, this increased the lifespan by 80% (Supplementary Information, Section N). This means that at moderate intensity ROS signaling may have life prolonging effects, even though high doses of ROS are killing. Indications for this inversion of role, depending on the intensity at which ROS impinges on its receptors, have been observed experimentally for hydrogen peroxide^20,24,96^. It is held responsible for lifespan extension by substrate (glucose) limitation in worm^18^. Supplementary Information (Section J) shows further how the model illustrates mitohormesis and preconditioning.

Only when the preconditioning ability of the network runs out, the ROS stress can establish a grip on the network and compromise ATP production and therewith any energy requiring function. Further upon this, the system exhibited bi-stability. It was either quickly stabilized, or collapsed immediately upon a strong increase of the ROS generation rate constant (Supplementary Fig. E.2). Once the drizzle of mutations caused the ROS generation rate constant to exceed a critical level, the deterioration became sudden.

Noting the paradoxical effect of an increased sensitivity towards oxidative stress resulting from overexpression of superoxide dismutase, Kowald et al.^75^ used a mathematical model to show the feasibility of an explanation involving a positive feedback loop. In line with the preconditioning effect discussed above, we propose that the superoxide dismutase overexpression reduces the level of superoxide anion, and thereby reduces the preconditioning too. In our comprehensive model, preconditioning further hinges on the roles of damaged mitochondria, the antioxidant set of proteins, and mitophagy. The mechanism we propose may still be linked to the mechanism modeled in ref. ^75^, which involved the H_2_O_2_ sensor peroxiredoxin^96–98^, as well as a mitochondrial matrix protease^99^. The model of Kowald et al. was made for a microorganism (*Podospora anserina*) however, and it is still unclear whether the same mechanism exists and produces mitohormesis in humans. A candidate human matrix protease might be the mitochondrial matrix ubiquitin protease system (UPS) that controlled the level of succinate dehydrogenase subunit A and therewith mitochondrial respiration in HeLa cells^99^.

### Nature: genetic perturbations of regulation modes and PD

All components of our ROS model have a place in the PD map prepared by Fujita et al.^58^. Perturbing these components, we were indeed able to simulate several scenarios of PD development, corresponding to several hypothetical PD patients. We here focused on perturbations of genes involved in the designs identified above.

Dynamics of p62 were involved in designs 3–5. Indeed, two PD scenarios were related to p62: either direct mutations compromising p62 functioning or sequestration of p62 by misfolded α-synuclein. In standard aging, the initially high level of ATP was maintained for over 50 years, after which a slow ATP decline (up to 75 years) (Fig. 5a) was followed by a rapid drop below 50% at around 100 simulation years. Both the direct compromise of p62 functioning (Fig. 5d) and the sequestration of p62 by misfolded α-synuclein (Fig. 5b) brought the ATP decline forward. This would explain the experimental observations reported by^61^ in terms of the mutations meddling with regulation modes 3–5.

Other scenarios were related to DJ-1 encoded by Park7 being mutated in some PD cases (Fig. 5c)^33^: At early age, patients with a reduced DJ-1 functionality (Fig. 5c, orange line; simulating a knock down) behaved similarly to the standard aging. In these in silico patients, a high concentration of ATP was maintained for only around 50 years. Assuming that ATP levels need to exceed 50% for vitality, the virtual patient with the DJ-1 mutation would not be vital at 55 years of age, but the homozygous deletion would already be nonviable at zero age, which may explain why homozygous DJ-1 mutation is not observed in the human population (Fig. 5c, red line). However when DJ-1 knockout was compensated with an increased Nrf2 activity (Fig. 5c, blue line), the behavior was similar to that of a DJ-1 knockdown. The modeled effect of the p62 mutation was more severe and sudden, the concentration of ATP being maintained for only around 30 years (Fig. 5d, orange line).

### Nurture: nutrition and PD

From the previous subsection, we might expect that activation of Nrf2–Keap1 signaling (regulation mode 3) by dietary compounds (e.g. by caffeine and other coffee components) might protect from oxidative stress. Our simulations confirm that a PD-related collapse due to oxidative stress might be delayed under coffee-based or antioxidant treatment (Fig. 5a). Thus, our detailed model reveals a network mechanism through which diet may play a PD protective role.

Our simulations also demonstrate that our virtual patients with increased levels of α-synuclein might be helped by both coffee and antioxidants (Fig. 5b). Virtual patients with the DJ-1 knockout compensated by Nrf2 signaling were affected more strongly by coffee (Fig. 5c). The virtual patient with p62 mutations was sensitive to antioxidants more than coffee (Fig. 5d). Thus, inter-individual variations may cause disease variability between individuals, as well as variability in therapeutic effects. Taking into account the possibility to identify the group of patients who would respond to therapy, coffee-based PD treatment could be more promising than the existing population or randomized-trial studies (with a modest negative correlation between PD and coffee consumption) suggest^62–65^.

The simulated effects of “coffee” are based on the assumption that coffee indeed affects the Nrf2–Keap1 signaling mode strongly and specifically enough. The effects modeled in Fig. 5a are overstatements of reality; the average strong coffee drinker does not live until the age of 150, and PD reduces lifespan only by a few years^100^. Coffee may not work as strongly on its receptors as modeled, its other effects accelerate aging, or our model is wrong after all. The importance of Fig. 5 is that it shows that in principle the model can deal with nutrition.

## Discussion

In this paper, we specified five types of robustness/homeostasis that may be important for ROS networks. We also identified five network mechanisms that serve to bring each of these in place. Integrating these mechanisms and then adding more detail, we then constructed a comprehensive model of aging and PD. The ROS module was validated by two experimental data sets. This model exhibited the time “warp” (distortion) that appears to be characteristic of aging: a long period of health followed by a much shorter period of drastic deterioration. We then found that the model explains that the critical time of aging is affected by mutations that have been related to PD, as well as by nutritional interventions such as caffeine. We also predicted new drug targets vis-à-vis aging and PD. We suggest that this is an example of what molecular systems biology can offer, but why?

One of the motivations for systems biology is the tenet that any biological function is effected by a network of molecules rather than by any molecule on its own^50^. Any failure of function, i.e. any disease, can thereby be due to any molecular failure that interferes with the functioning of the corresponding network. A therapy could be anything that restores network function, even if the molecular malfunction first causing the disease remains in place. Virtually all molecules in the cell are connected with each other however^49^. Accordingly, the many different biological functions of a cell (or organism) somehow correspond to the functioning of the single total network and all different diseases correspond to malfunctions of the same network. If this is so, how are we ever going to find specific network cures for any specific disease?

The answer may be that biological functions correspond to functional modes of the network and diseases represent failures of those modes. This concept may be clearest for inborn errors of metabolism, where genetic errors interfere with the functional flux mode from nutrition to an essential molecule (e.g. brain tyrosine for phenylketonuria). Using flux balance analysis (FBA) such flux modes and their impairments can be identified^101^, but FBA depends on applying the mass conservation principle to every network node, and is thereby inappropriate for regulatory networks. A different methodology would be required for networks that abound in signal transduction or gene expression. In the present paper we implemented such a methodology in the highly complex network that manages age-related PD. The role played by the metabolic-flux mode in the FBA of inborn errors of metabolism is taken by what we called the “regulation mode”, or “design”. For the PD network we have identified five such designs, each corresponding to a regulatory function of the network. These functions were stationarity, robustness, homeostasis, dynamic robustness, and repetition robustness. The corresponding five networks are depicted in Fig. 1. In this way, the present paper documented five biological functions and five network mechanisms. The way we dealt with these five functional notions, i.e. by testing whether they would work in a mathematical model, is similar to^76^ elegant use of mathematical models to evaluate various other ideas about ageing.

Some of these functions may appear almost trivial. This may be true for the function of stationarity, but less so for the fifth function of robustness against repeated challenges; it is not among the standard requirements formulated for regulatory pathways. Not surprisingly, also the corresponding design is fairly complex (Fig. 1e).

How certain can we be about these five dynamic robustness functions and five regulatory modes that support them? For functions carried by a single protein, such certainty is obtained by modulating the gene expression level of that protein. For network functions a validation is much more complicated. It is virtually impossible experimentally to increase the levels of all proteins in the subnetwork corresponding to the function by the same factor without changing the expression levels of many other proteins. An alternative is in silico validation, where one develops a precise mathematical model of the network, validates this model experimentally, and discovers the network functions and mechanisms in silico. This is the molecular systems biology strategy that we used in this paper. The problem with this strategy is that the experimental validation is still incomplete, but as this validation becomes more and more complete in the future, we can continue to check whether the network functions, and disease mechanisms documented here, continue to be supported. Yet, the findings of this paper should be taken with appropriate reservations. Our discoveries are tentative, but should not this be so for all scientific discoveries^102^?

As complex as our “comprehensive” model of more than 60 substances and processes, and well over a hundred parameters may be, it is a vast simplification of reality. Our model only contains a fraction of all that is known about the networks they deal with. It lacks processes such as mitochondrial fusion–fission, the apoptotic pathways downstream of Cyt *C*, proton pumping, as well as redox metabolism, MAP kinase signaling, p38, p53, HIF1, HSF-1, and PTEN actions. It fails to differentiate between the various molecular species all too often brought under the common denominator of “ROS”^75,103^ and it is not cell-type specific, but rather a blueprint model that will need to be fine-tuned for every new cell type (indeed, the limited experimental validation used two different cell types). Another issue is the physical and chemical constraints, such as the maximum for the mitochondrial concentration. We have dealt with this issue implicitly by showing that with our five designs in place, mitochondrial concentrations would not change much when the network is challenged (Fig. 2e).

In all these senses, our “comprehensive” model (model D) is *not* quite comprehensive; it is an overly small “blueprint” model. From another perspective, the model is too big already: The two sets of experimental results presented in this paper and found to be consistent with our model are nowhere near a complete validation. Our comprehensive model would require 12,000 data points concerning intracellular concentrations, obtained after 100 independent experimental modulations and at the second timescale for a more or less complete validation. The model should then be refined using a procedure managing the expected uncertainties in both parameters and variables, for which the computational power is not yet available^104^: *complete* validation is impossible therefore at this moment in time.

How could our model be successful in its predictions, in the absence of such complete validation? We surmise that reality is simpler than it could have been theoretically: many fewer substances interact directly than theoretically possible and interactions are indeed subject to diffusion limitation and reasonable thermodynamics leading to the default set of dissociation equilibrium constants that we put into our model. Apparently, a model as limited in complexity and validation as our comprehensive model can produce known function and malfunction. We conjecture that the ROS network is not as complex as it seems. Five much simpler subnetworks explain its robustness functionalities. Because it is five network structures rather than five molecules, the system appears complex. Our network analysis thereby reduces the complexity tremendously, without reducing away the essence.

On the other hand, this should serve as a warning that activities attributed in our model to certain molecular factors may in fact be due to other factors, which we neglected or overlooked. Further validation and analysis are needed therefore, but at least now there is something to be validated and analyzed.

At present, the validation of our models in this paper, vis-à-vis two experimental data sets, is still limited. In view of the tremendous complexity of the model, this validation is highly incomplete but a full validation also impossible at present. We can only hope that the very public availability of our model through this publication will serve as a further support of validation of the model itself or of improved variants thereof. An inspiring type of validation of a model occurs when persons not involved in its construction ask whether the model can reproduce a hitherto unaddressed experimental observation of functional interest. One of the reviewers of this paper asked whether the model could reproduce mitohormesis^18,20,24^ and whether increasing ROS generation could decrease the toxic effects of ROS. The reviewer also asked whether the model could illuminate the dual role of Nrf2, i.e. in the embryonic lethality of Keap1 deficiency^45^ and in the invigoration of adult cells, e.g. as driver of hallmarks of cancer such as resistance to apoptosis^35,46^. In the Supplementary Information, we now show that without any further modification the model could execute all these three tasks: this triple challenge resulted in a triple validation.

The large number of parameters and the model’s nonlinearity also come with the possibility that even the qualitative conclusions we have drawn may not be valid for parameter values different from the ones we have used here: the parameterization of our models may have affected the in silico observations even qualitatively. We have performed two sensitivity analyses to begin to deal with this problem: The Supplementary Information shows the sensitivity coefficients for the steady-state concentrations. The control coefficients shown in Table 1 are themselves sensitivity coefficients for the dependence of the predicted lifespan on all process rate parameters. The former sensitivity coefficients are largely below 1 and all below 6 in absolute magnitude, showing that the model is well-behaved in and immediately around our standard set of parameter values. The control coefficients are below 3, again reflecting model robustness. A comprehensive sensitivity analysis of our model of more than 100 parameters is impossible, as it would take many years. A sensitivity analysis for a much more limited number of parameter values chosen at random would under-sample massively. Our method of classifying parameter and variable values in terms of uncertainty and then scanning the least reliable ones most^104^ is one that we envisage to develop for the near future for this model. With this we will then try to integrate all relevant literature data on ROS plus new data to be generated in our own laboratories.

That the network of the comprehensive model is nonlinear kinetically, has the consequence that the regulatory role of any of the above five designs is influenced by the presence or absence of the other designs. This is a property inherent to nonlinear conglomerate systems, and therefore something we have not tried to minimize, not even for didactic reasons: this paper is research in the (in silico) discovery mode, not in the review and teaching mode a posteriori. While we find both modes highly important, we also prefer to keep them apart in separate publications.

A related issue is whether the designs continue to execute their stabilizing function in the comprehensive model. They do this: As described in Supplementary Information (Sections E and F), we checked how the five designs identified above with the use of simplified models continue to work in the detailed model. Memory and preconditioning persisted (Supplementary Information, Section E). The first three designs worked in a straightforward way: without mitophagy (Design 1) the system did not reach any steady state (Supplementary Fig. F.1), without limiting the mitochondrial synthesis (Design 2) the structural robustness was lost (Supplementary Fig. F.2), and without Keap1–Nrf2 signaling (Design 3) the homeostasis was substantially decreased (Supplementary Fig. F.3). Homeostasis was not lost completely, because other feedback mechanisms, such as NFκB and DJ-1 signaling, were still working in the system.

Still, the nonlinearity of the system does have an implication for the way one can analyze the network and present the results of this analysis. The functioning of the five subnetworks depends to some extent on the order in which the subnetworks are introduced, i.e. on which other subnetworks are already present when any of the networks is introduced. One can thereby not present the five design in a form unequivocally allowing effects to be attributed to subnetworks/functions. We have chosen to use our “domino approach”^72^ to analyzing this complex system.

We began with the simplest subnetwork, most connected to the essential cellular function of mitochondrial oxidative phosphorylation, but producing ROS. We then added subnetwork 2, which was the next closest connected to the ROS. We then added the next network closest to this, etc. This has been our in silico discovery mode. We followed the same order in the presentation of our findings in this paper. An alternative way of presenting the various regulatory modes, may be to first execute some sort of orthogonalization and then present the resulting “normal modes”. Such orthogonalization should be valid only locally (i.e. for one set of parameter values, and in one consequent state) and might well result in somewhat abstract combinations of the five networks that we have considered here. Although the result should be purer mathematically and independent of the sequence of presentation, it would be less comprehensible biologically. Moreover, we are here not addressing a metabolic network where one could orthogonalize a single stoichiometric matrix *N*; we here have a combination of reaction stoichiometry and other regulatory interactions, and it should not necessarily make sense to treat these equally in the presentation. Yet another method of presenting might optimize from the didactic point of view. In view of the complexity of these issues we should like to postpone the development of the optimal way of precisely finding and presenting the regulatory modes, to a paper completely focusing on this issue.

To the extent possible, given current knowledge and the limited complexity we put in place, the predictions by our model appear to be valid. But, are they relevant beyond the provision of academic understanding of what we already know? Could they help identify new drug targets to cure PD, delay aging or otherwise help manage perturbations of functions? We have shown that when reassembled into a comprehensive network, the five subnetworks come with a set of drug targets. Table 1 shows control coefficients identifying that at least 25 out of the 57 processes considered determine ageing tenfold more than proportionally. This is a surprisingly high number as compared to drug targets in metabolic and normal signal transduction pathways, which are usually less than proportional. As the network steps with the highest negative time-control coefficients should be the best drug targets, 12 targets may be relevant. For a much smaller model than ours, Kowald et al.^75^ also showed that multiple factors controlled six concentration variables including superoxide anion and hydrogen peroxide.

One may wonder whether these many drug targets are not too tentative. The answer is that they are the best available and offer an equal number of ways to validate (and perhaps improve) the present study. It would not be the first time that this strategy proves to effective^105^.

Another way in which our comprehensive model of aging appeared to be useful was in reproducing/predicting the peculiar aspect of healthy aging that we called the “time warp”: a delayed aging process emerged from quick processes in the network. This is an example of multiscale modeling, where the minutes’ timescales of molecules are shown to be relevant for a hundred year process in a multicellular organism. The model reproduced this even though there were no time constants in the range of 1/100 year. On the contrary, all processes in the model were molecular systems biology processes at a timescale much faster than 1 h. The frequency of mutations was approximately 2 per year around the time of the time warp, i.e. at 100 years of age. Somehow we here came across one of the difficult issues of systems biology, i.e., the multiscale nature of biology, in this case the multi-timescale nature.

We examined what could be the basis of this timescale jump from minutes (molecular) to 100 years (whole human aging) and more particularly of the fact that initially (for the first 70 years) the ageing seemed to be without effect, to become effective progressively only later. We found that the ROS network exhibited preconditioning, i.e., the phenomenon that it became more robust to a challenge after it had experienced it already. This could then of course lead to the effect that a repetitive challenge would be ineffective, a phenomenon also produced by our fifth design. There may well be another or additional reason however. This is the phenomenon that control coefficients tend to be small, but increase when the controlling factor is jeopardized. If that factor is being jeopardized progressively, this will at first have little effect, but subsequently a sudden and strong effect as in aging^93^.

More particularly for the PD network, our study revealed the following designs (regulatory flux modes) and the roles of molecules: (i) mitophagy enabling steady state (Design 1), (ii) the dynamically variable concentration of mitochondria ensuring that that steady state is structurally robust against fluctuations in ROS generation (Design 2), and (iii) mitochondrial recovery via NFκB signaling serving robustness against increased ROS production. Paradoxically, a high rate of mitochondrial recovery is not always beneficial, but may harm the cell if ROS generation suddenly fluctuates (Design 4).

We have shown that mitochondrial recovery and mitophagy should also be coordinated with the antioxidant response. We have identified roles of Nrf2–Keap1 (Design 3) and DJ-1 (Design 5) in this coordination. The Nrf2–Keap1 system works as ROS sensor and forms the first line of defense by activating the antioxidant response and mitophagy^106,107^. DJ-1 is an additional ROS sensor that amplifies the activity of Nrf2–Keap1 signaling and coordinates it with mitochondrial recovery^32^. Our models predicted that DJ-1 upregulation increases the cell’s robustness, whereas its downregulation should sensitize the system to oxidative stress. Indeed, some cancers are associated with upregulation of DJ-1^108^, while some cases of neurodegeneration are related to DJ-1 mutation or downregulation^33^. It is remarkable that the same components are oppositely mistuned in opposing diseases: in cancer the cell survives elevated ROS, while in neurodegeneration the cell dies from ROS. Taking into account that DJ-1 is localized mostly in the mitochondria, whereas Keap1 is localized in the cytoplasm, one could foresee the importance of adding spatial aspects to the complexity of ROS management. Here we have done this in the simplest way, i.e. by discussing three compartments, i.e. nucleus, cytosol and mitochondrion, but without being explicit about their volumes.

When we increased the resolution of model B5 to obtain the comprehensive model D, dynamic homeostasis emerged: several consecutive pulses of increased ROS generation (mild oxidative stress), “train” the ROS management system to deal with subsequent larger stresses^96^. This may explain several paradoxes reported in the literature, for example, those related to the observations that antioxidants may exhibit a hormesis response^109^ and that, in some cases, antioxidant therapies disappoint clinical experience in cancer treatment^110^.

Our modeling results are also compatible with previous reports^111,112^ demonstrating how the sequel of oxidative stress events may lead to the development of PD via a vicious cycle. For example, when rats were exposed to three pulses of paraquat (PQ) in order to mimic oxidative stress, the second addition allowed the system almost perfectly to compensate for the stress (adaptation), while the third pulse of PQ caused again a larger effect (and ultimately perhaps collapse)^113^. The authors explained these phenomena by bi-stability^111,112^. Our models also contain bi-stability and may allow a similar interpretation, the first pulse of PQ leading to quick adaptation via activation of an antioxidant response and mitophagy that make the system more tolerant to the consecutive mild stress, but with the third pulse of PQ exceeding the threshold of the accumulated ROS-induced damage, with the system then collapsing.

In several simulations, we observed that activated Nrf2 oscillated transiently (model 3). This correlates with literature data^114^ showing that Nrf2 undergoes autonomous frequency-modulated oscillations between cytoplasm and nucleus. Oscillations occurred when cells were stimulated at physiological levels of activators. They decreased in period and amplitude and then evoked a cyto-protective transcription response. According to the data shown in ref. ^17^, Nrf2 might be activated in cells without a change in total cellular Nrf2 protein concentration. In our model, an activation of Nrf2 is also related mostly to the shift between nuclear and cytoplasmic localization.

Metabolic reprogramming may vary the NADH/ATP/ROS ratio, as well as the sensitivity to oxidative stress^115^. Such metabolic reprogramming corresponds to changes in the operating regime of our blueprint model, for example, where we use the NADH redox level as our model’s input and the ATP hydrolysis work load as one of our model’s outputs. Addition of this extra complexity is yet another potential application of our blueprint model. This also brings the challenge of explaining the effect of ketogenic diets on lifespan through its effects on mitohormesis^96^.

We built our models largely ab initio, i.e. starting from the molecular cell physiology of the response to oxidative stress and increasing the complexity of the network step by step. Adding every new level of complexity in a domino approach enabled us to identify designs of ROS management. We feel that this initial simplicity helped this identification to succeed. At the same time, our most complex model, which still comprised these designs, became a blueprint model into which the information from the available disease maps could be projected. Overlaying our blueprint model with the PD map^58^, we obtained several PD-related patient-specific models.

When examining a particular patient with single-nucleotide polymorphisms (SNPs) in a set of relevant genes, such particularization will become important and possible. We entertain the concept that an individual’s genome may be examined for SNPs in pro-and anti- apop- and mitop- totic factors, the implications then becoming predictable by more detailed versions of our models. Even before their precise validation, our calculations may serve as case studies connecting data-driven biomedical disease maps with systems biological dynamic models built ab initio. They then serve as proofs of concept showing how personalized medicine might benefit from such connections and how fundamental design studies may inspire practical biomedical questioning.

Whether the designs we identified do operate more generally should now be a matter of further experimental validation. In a way this paper merely helped to recognize what important principles are there to be validated in the near future. We did present two lines of model validation, but much more such validation is needed.

In conclusion, we showed how to deal with overlapping functions of complex regulatory networks by identifying a limited number of regulatory flux modes. We implemented this for PD and aging, showed how this can be used for network-based drug target identification, and gave several examples of how nature and nurture interactions may be identified, and personalization may be achieved.

## Methods

### Model building

The comprehensive model has 63 substances, 61 reactions, and more than 100 parameter values. The basis for substances and for the processes between them is the networks of ROS management known from the scientific literature. Model diagrams (e.g. Figs 1 and 3) were generated using CellDesigner (v4.4; Systems Biology Institute, http://celldesigner.org/index.html), a graphical front-end for creating process diagrams of biochemical networks in Systems Biology Markup Language^116^. CellDesigner-generated models were transferred to COPASI (v4.6, build 32) (www.copasi.org), which is another Systems Biology Markup Language-compliant program, but with a wider variety of analysis options. For each reaction, a kinetic term can be included, detailing the interactions between the species.

### Model-stability checks

Two procedures to check model stability were used: (I) We took the final state as the initial state (Copasi enables one to do this with a single click) and then ran the model again and checked that then nothing changed with time. Subsequently, the initial condition of some variables were changed somewhat (e.g. by a factor of 2) and a time calculation was started, again checking that the same final steady state was reached. (II) We ran the steady-state mode of Copasi and then activated its stability analysis, checking that none of the eigenvalues were reported to be positive.

### Model parameterization

Several parameter values were identified from the literature. Other parameter values were initially chosen in the physiological range and adjusted to achieve the biologically meaningful steady state. When validating the model by the experiments with menadione-induced oxidative stress the parameters were adjusted to reproduce the experimental data. When validating the model with the data on the changes of the ATP concentration induced by H_2_O_2_, only the rate constants of (i) H_2_O_2_ transport to the cell, (ii) H_2_O_2_ degradation, and (iii) H_2_O_2_-induced ROS generation were adjusted. All other parameters were the same as in the experiments with menadione.

### Model fitting

Parameter values used within the model were fitted to known biological behavior of the system, while maintaining these parameters and even more so the consequent dependent variables within previously determined or proposed, biologically realistic bounds, such as diffusion limitation, known equilibrium constants for associations, and known intracellular concentrations. The known biological behavior included the concentrations of substances like ATP (a few mM), ROS (nM range), proteins (nM range), mRNA (thousands) and genes (diploid numbers). Little of this was consequential for the behavior of the model, as the second-order rate constants (which are virtually unknown in practice for any single cell type) can be adjusted to the concentrations without affecting the more relevant quasi-first-order rate constants, and the fitted behavior was considered definitive, for this presentation. The fitting was done manually and strategically, i.e. by identifying and then tuning parameters with uncertain values close to what was modulated experimentally. In view of the above, the parameter values in the models of this paper should be considered with caution and not be applied uncritically in other contexts without more precise experimental validation.

### Modeling of the mechanism underlying the aging

Without the aging process the model produced a steady state. We added a slow aging process through a new variable called “ROSAging” the time increase of which was made proportional to [ROS]^4^. The ROS synthesis rate was proportional to 1 + the value of ROSAging multiplied by a constant factor. Mutations were modeled by halving or zeroing gene dosages, or otherwise altering activities as specified in legends.

### ATP experiments (with H_2_O_2_ perturbations): cell cultures and treatments

PC12 (clone 615; overexpressing the TrkA receptor was kindly provided by M.V. Chao (Skirball Institute, New York University School of Medicine, NY)) cells^117^ were maintained in Dulbecco’s modified Eagle’s medium supplemented with 10% fetal bovine serum, 5% heat-inactivated horse serum, 2 mM l-glutamine, 100 µg/ml streptomycin, 100 U/ml penicillin in a humidified atmosphere of 95% air 5% CO_2_ at 37 °C, as previously described^118^. All cell culture reagents were purchased from EuroClone (Milano, Italy).

### ATP experiments (with H_2_O_2_ perturbations): measurement of H_2_O_2_ half-life

For measurement of H_2_O_2_ half-life, PC12 cells were treated with H_2_O_2_ (50, 150, or 300 µM). Aliquots of the culture medium were taken at the indicated times and analyzed for H_2_O_2_ content using the AMPLEX RED Hydrogen Peroxide KIT (Life Technology). Values were normalized by the protein content.

### ATP experiments (with H_2_O_2_ perturbations): ROS analysis by flow cytometry

Determination of intracellular levels of total ROS was carried out by flow cytometry using 2′,7′-dichlorodihydrofluorescein diacetate (DCH2FDA, Thermo Fisher Scientific), as previously described^119^. PC12 (2 × 10^5^ cells/well) were plated in six-well plates (EuroClone) pre-coated with poly-l-lysine (0.1 mg/ml). DCH2FDA was added during the final 30 min of treatment. Cells were then washed with PBS, harvested with 0.08% Trypsin, and analyzed by FACS (FACScan Becton-Dickinson, San Jose, CA) using the Cell Quest Software (BD Bioscience). Fluorescence was measured on 1 × 10^4^ cells and data were analyzed by using the Flowing Software 2.5.1 (Turku Centre for Biotechnology, University of Turku, Finland).

### ATP experiments (with H_2_O_2_ perturbations): ATP determination

PC12 cells were plated into six-well plates (7 × 10^4^ cells) and exposed to H_2_O_2_ for the indicated times. Cells were then lysed by using lysis buffer and ATP activity was analyzed by using the Adenosine 5′-triphosphate (ATP) Bioluminescent Assay Kit (Sigma-Aldrich) according to the manufacturer’s instructions. The light intensity was measured using a luminometer (Lumat LB9507, Berthold) in a 5-s time period and expressed as relative light units/μg of protein.

### ATP experiments (with H_2_O_2_ perturbations): statistical analysis

Data are presented as the mean ± SEM of the number of independent samples in separate experiments, as indicated in the figure legends. Statistical analysis was performed by using GraphPad Prism 6.0 (GraphPad Software, La Jolla, CA, USA). All quantitative data were analyzed by one-way ANOVA and Dunnett’s multiple comparisons test for multiple treatments or by Student’s *t*-test for single comparisons (*p ≤ 0.05, **p ≤ 0.01, ***p ≤ 0.001 versus control (CTR)), as indicated in the figure legends. For morphology analyses, individual images of CTR and treated cells were assembled and the same adjustments were made for brightness, contrast, and sharpness using Adobe Photoshop (Adobe Systems, San Jose, CA).

### Menadione experiments: transcriptomics

In the experiment HepG2 cells (ATCC, catalog number HB-8065) were exposed to menadione and a consequent increased ROS intracellular concentration and via microarray analysis of whole-genome gene expression the fold change in the expression of p62, Bclxl, and NQO1 (the gene representing the antioxidant response) were measured. The experiment lasted 24 h. For the measurement the following time points were chosen: 0.5, 1, 2, 4, 6, 8, and 24 h after menadione addition. The data used in this paper were taken from the dataset deposited inNCBI’s gene expression omnibus^120^ are accessible through GEO seriesaccession number GSE39291. The experimental methodology wasdescribed in ref. ^121^. It is based on microarray confirmed by PCR.

### Menadione experiments: cell culture, menadione dose selection, and ROS formation

HepG2 cells (ATCC, LGC logistics) were cultured in six-well plates in the presence of minimal essential medium supplemented with 1% nonessential amino acids, 1% sodium pyruvate, 2% penicillin/streptomycin, and 10% fetal bovine serum (all from Gibco BRL, Breda, The Netherlands). The cells were incubated at 37 °C and 5% CO_2_. When cells were 80% confluent, the medium was replaced with medium containing menadione (Sigma-Aldrich, Zwijndrecht, The Netherlands). As a solvent control, just medium was used. A non-cytotoxic concentration of menadione was selected based on a 3-(4,5-dimethylthiazol-2-yl)-2,5-diphenyltetrazolium bromide cytotoxicity assay with 80% viability after 24 h exposure. In addition, using ESR spectroscopy, the final concentrations of 100 µM menadione was determined based on maximum oxygen radical formation at a non-cytotoxic dose. Radical formation in HepG2 cells was measured by ESR spectroscopy in combination with the spin trapping technique using 50 mM 5,5-dimethyl-1-pyrolline N-oxide (Sigma-Aldrich). Protein oxidation was confirmed via a protein carbonyl assay and oxidative DNA damage via detection of this using the Fpg comet assay^121^.

## Supplementary information

Supplementary Information

## Data Availability

Source data for all figures, mathematical models for simulations in COPASI (cps format), and simulations results are in Open Access at FAIRDOMHub (10.15490/FAIRDOMHUB.1.INVESTIGATION.399.3)^122^. This DOI is assigned for a snapshot of an entire catalog where all materials are ordered according to figures and Supplementary Information in the manuscript. See Supplementary Information for detail. Additional DOIs are assigned for the final “Detailed model of ROS management” (10.15490/fairdomhub.1.model.571.1), calibrated version of this model called “Calibrated comprehensive model of ROS management” (10.15490/FAIRDOMHUB.1.MODEL.643.1)” and calibrated model of ROS management tuned for validation experiments with menadione called “Calibrated comprehensive model of ROS management Improved” (10.15490/FAIRDOMHUB.1.MODEL.734.1). In the main catalog, these models are grouped under the directory “Main ROS Detailed models” and are also publicly accessible. In the annotations of the models, instructions are given on how to reproduce the various figures of this paper by running those models. The models can be simulated online by FAIRDOMHub registered users. The COPASI versions of the model inclusive of the parameter values used can be downloaded.
