# Supplementary material for: ROS networks: designs, aging, Parkinson’s disease and precision therapies
Source: NPJ Syst Biol Appl. 2020 Oct 26;6:34. doi: 10.1038/s41540-020-00150-w (PMC7589522; doi:10.1038/s41540-020-00150-w)
Supplement: Supplementary file 1 — Supplementary Information [file 41540_2020_150_MOESM1_ESM.pdf]

Supplementary Information

Table of Contents

Section A. The simplest "no-design" module. Design A. Model A. 3

Section B1. Model B1. 4

Section B2A. Design 2A. Models B2.A1-B2.A3 6

Section B2B. Design 2. Model B2B. 10

Section B3. Design 3. Model B3. 13

Section B4. Design 4. Model B4. 17

Section B5. Design 5. Models B5.1-B5.3A. 22

Section C. Design C. Model C. 30

Section D. Comprehensive Model D: fitting to experimental data 36

Section E. Memory in comprehensive Model D 40

Section F. Re-examining 5 designs in the detailed model 42

Section G. Aging with compromised NFkB signaling 47

Section H. The effect of α-synuclein on ATP and ROS concentrations in the detailed model . 49

Section I. Aging-time-control coefficient 51

Definition 51

Determination 51

Section J. Control of aging 53

Section K. Calibration of the comprehensive model. 54

Changes without effect other than factorial changes in concentrations 55

ATP/O stoichiometry, e/ATP stoichiometry...55

Cytochrome c concentration 55

Mitochondrial concentration 55

Changes without effect on steady state concentrations but with effects on the dynamics .. 55

Mitochondrial turnover 55

Changes with effect on steady state concentrations and the dynamics 58

ATP/mitochondrial synthesis stoichiometry 58

Section L. Parameter values 59

Section M. ROS and aging 65

Section N. Triple extra validation: mitohormesis, preconditioning and dual role of NRF2 .. 66

Section O. Sensitivity analysis 69

Section P. On the roles of Parkin, p62 and mitochondria in the various designs, as dependent versus independent variables 70

Section A. The simplest "no-design" module. Design A. Model A.

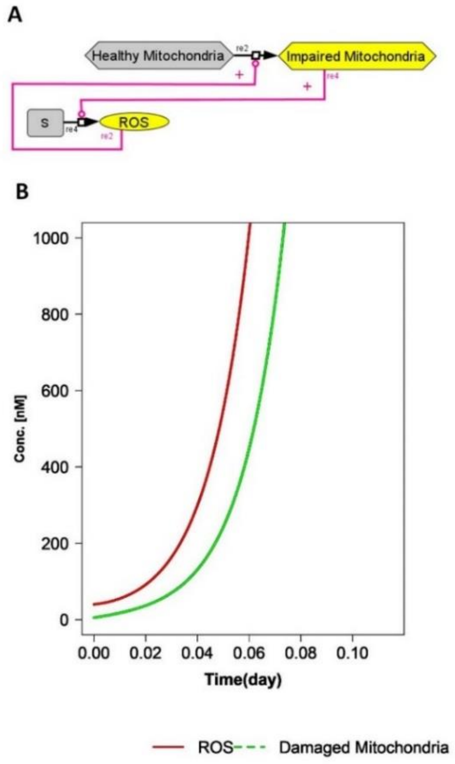

Supplementary Figure A.1. The 'No design' Model A. Network diagram showing the positive feedback loop formed by ROS damaging the mitochondria (re2) and damaged mitochondria producing ROS (re4). **B.** Simulated concentrations of ROS and damaged mitochondria (model A.cps) corresponding to the "no-design" case.

The following conclusions were drawn from Supplementary Figure A.1:

The system is unstable and is not able to reach a steady state.

Model building and simulations:

Model A.cps was used for generation of the figure. The model and the simulation results have been assembled into the model archive file called 'SM-A-no design'. The general idea used in the modelling is described below:
The reaction of ROS generation is catalyzed by impaired mitochondria at the rate described by the mass action equation:

Rate of ROSgeneration=ROSsynCoef*([DamagedMitochondria(t)]).

Thus, in the absence of damaged mitochondria ROS are not produced. Analogously, the mitochondrial aging is catalyzed by ROS at a rate described by the mass action equation: Rate of Mitochondrial Aging=k*([HealthyMitochondria(t)]*[ROS(t)]).

Thus, in the absence of ROS, mitochondria are not damaged. Simulations started at initial conditions where the concentration of ROS was equal to 10^{-5} (nM) and the concentration of

damaged mitochondria was equal to 10^{-5} (arbitrary units). Time courses for ROS and Damaged Mitochondria were simulated.

No steady state is observed. The concentration of both ROS and Damaged Mitochondria varies exponentially with time ('exploded').

Section B1. Model B1.

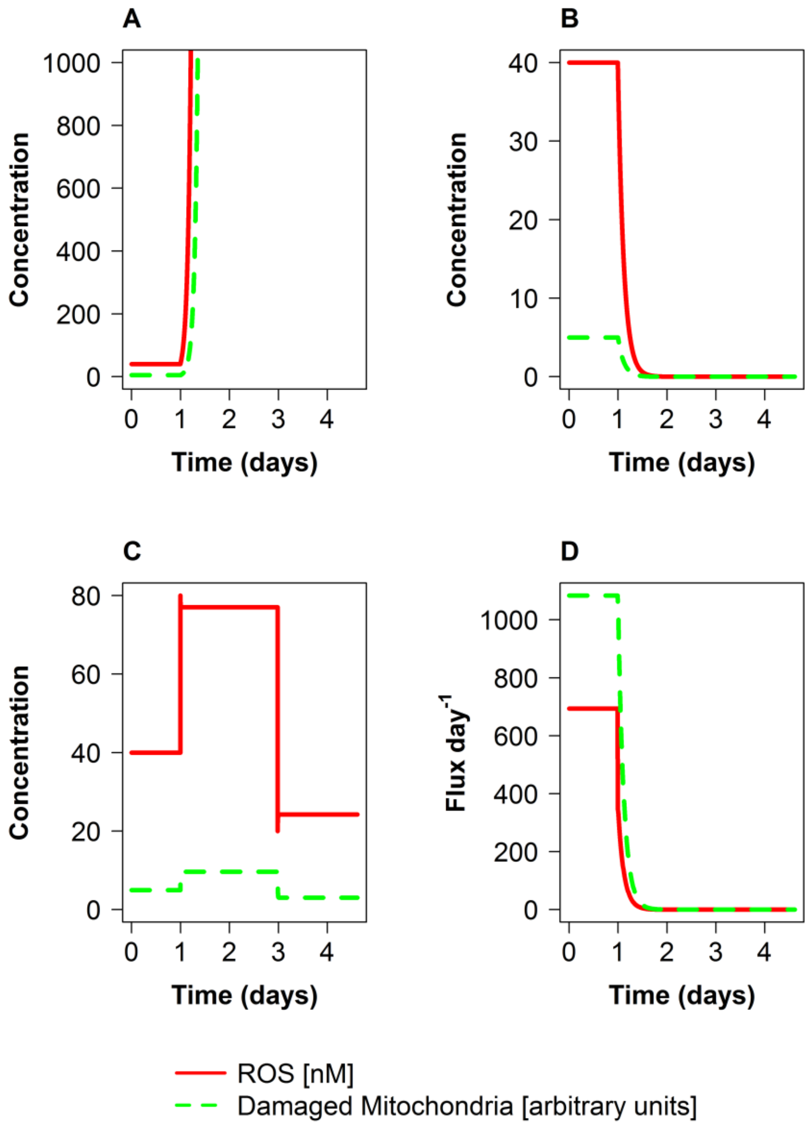

Supplementary Figure B1.1. Steady state in the ROS core model. Structural and dynamic instability of the ROS core model (Model B1; design 1).

Concentration of ROS (in nM) and damaged mitochondria (in arbitrary units) (A-C), fluxes of ROS production and generation of damaged mitochondria (D) for doubling ROS generation rate constant on day 1 (A), for halving the rate constant on day 1 followed by its restoration to its original value on day 3 (B, D), and for injection of ROS at day 1 followed by a wash out on day 3 (C).

The following conclusions were drawn from Supplementary Figure B1.1:

When simulations starts from a specific set of initial conditions, the initial state persists if a steady state is maintained. However, when the ROS generation rate constant is doubled on day 1, both the concentrations of ROS and of damaged mitochondria increase indefinitely and the system 'explodes': the state is structurally unstable. When the rate constant of ROS production is instead halved, the system relaxes irreversibly to the "quasi-perfect" state with the concentrations of ROS and damaged mitochondria equal to 0. The fluxes in the systems disappear accordingly. When on day 3 ROS generation is returned to its initial rate, the system stays at the same "quasi-perfect" zero-state. When at the initial steady state ROS are injected or eliminated, ROS concentration changes as expected, but does not relax back to its initial value, showing that the steady state is not dynamically stable in the sense of Lyapunov stability.

Model building and simulations:

Model B1 (B1.cps) used for generation of every figure, as well as simulation results have been assembled in the model archive file SM-B1.

The general idea used in the modelling is described below:

Generation of ROS is catalyzed by impaired mitochondria at a rate described by the mass action equation: Rate of ROSgeneration=ROSsynCoef*([DamagedMitochondria(t)]). Thus, in the absence of damaged mitochondria ROS are not produced.

Analogously, mitochondrial aging is catalyzed by ROS with the rate described by the mass action equation: Rate of Mitochondrial Aging=k*([HealthyMitochondria(t)]*[ROS(t)]). Thus, in the absence of ROS, mitochondria are not damaged.

Both rates of removal of ROS and removal of impaired mitochondria (mitophagy) were modelled as mass action reactions:
Rate of ROS removal = k*[ROS(t)]*[Antiox(t)]
Rate of Mitophagy = k*[Impaired mitochondria(t)]*[parkin(t)] *[p62(t)]

Antiox was included in the reaction as catalyst (i.e. without being consumed) because we considered not only antioxidant metabolites (e.g. various antioxidants which the cell would maintain at constant concentrations), but also enzymes (e.g. superoxide dismutase) catalyzing ROS removal. P62 and parkin are consumed for mitophagy as substrates. Antiox, p62 and parkin concentrations are taken constant, i.e. independent of time. Simulations started with initial conditions where the concentration of ROS was equal to 40 nM and the concentration of damaged mitochondria was equal to 5 nM. Time courses for ROS and Damaged Mitochondria were then simulated using Copasi.

Section B2A. Design 2A. Models B2.A1-B2.A3

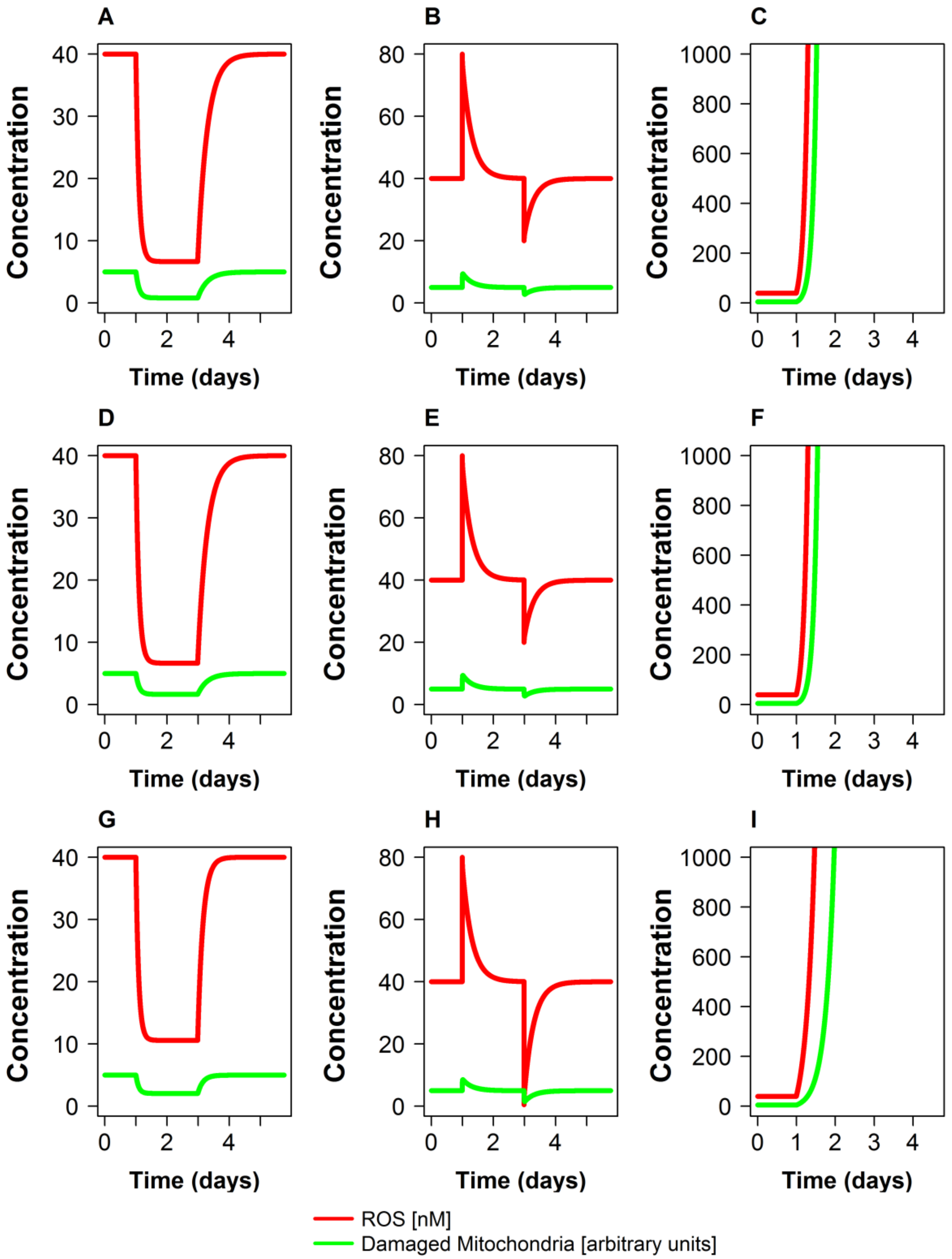

Supplementary Figure B2A.1 (Models B2A.1-B2A.3). Dynamic stability obtained when basal ROS generation and/or basal mitoptosis (i.e. autophagy specific for impaired mitochondria) were added to Design 1 thereby obtaining Design 2A. Concentrations of ROS (in nM) and damaged mitochondria (in arbitrary units) for three subtypes of Design 2A. Simulations used a model with basal ROS generation only (A-C; top row: model B2A.1-BasalROS.cps), a model with basal mitoptosis only (D-F; middle row; model B2A.2-BasalMitochondrialAging.cps) or a model with both basal ROS generation and basal mitoptosis (G-I; bottom row; model B2A.3-BasalAll.cps). In A, D, and G, ROS generation rate constant was first decreased 2 fold (day 1) and then returned back (day 3). In B, E, and H, ROS concentration was instantaneously increased two fold (day 1) and then decreased two fold (day 3), whereas in C, F, and I, ROS generation rate constant was increased 2 fold on day 1.

The following conclusions were drawn from Supplementary Figure B2A.1:

A) Concentrations of both ROS and damaged mitochondria follows the perturbation of ROS synthesis. Concentrations of both species decrease 6-fold to a lower steady value and then return to their initial levels. **B)** Concentrations of both ROS and damaged mitochondria transiently respond to perturbations, but both concentration changes are transient: ROS and damaged mitochondrial concentrations ultimately return to their initial levels. **C)** The system explodes. **D)** The concentration of ROS follows the perturbation, first decreasing 6-fold and then returning to initial level. The concentration of damaged mitochondria follows the perturbation, first decreasing 3-fold and then returning to initial level. **E)** Concentrations of both ROS and damaged mitochondria transiently respond to perturbations, but both concentration changes are transient: ROS and damaged mitochondrial concentrations ultimately return to their initial levels. **F**) The system explodes. **G**) ROS concentration follows the perturbation, first decreasing around 4-fold and then returning to initial level. The concentration of damaged mitochondria follows the perturbation, first decreasing around 2.5-fold and then returning to initial level. **H**) Concentrations of both ROS and damaged mitochondria transiently respond to perturbations, but both concentration changes are transient: ROS and damaged mitochondrial concentrations ultimately return to their initial levels. I: The system explodes.

We conclude that for limited extents of perturbation, the system is both structurally and dynamically stable, whilst for substantial upward perturbation of ROS production, the system remains unstable.

Model building and simulations:

A compendium of models B2A.1-B2A.3 (B2A.1-BasalROS.cps, B2A.2-BasalMitochondrialAging.cps and B2A.3-BasalAll.cps) used for generation of every figure, as well as simulation results have been assembled in the model archive file SM-B2A.

The general idea used in the modelling is described below:

Both rates of removal of ROS and removal of impaired mitochondria (mitophagy) are modelled as mass action reactions:

Rate of ROS removal = k*[ROS(t)]*[Antiox(t)]

Rate of Mitophagy = k*[Impaired mitochondria(t)]*[parkin(t)] *[p62(t)]

Antiox was included in the reaction as catalyst (i.e. without being consumed) because we considered not only antioxidant metabolites (e.g. various antioxidants which the cell would maintain at constant concentrations), but also enzymes (e.g. superoxide dismutase) catalyzing ROS removal. P62 and parkin are consumed for mitophagy as substrates. Antiox, p62 and parkin concentrations are taken constant, i.e. independent of time. Simulations started with initial conditions where the concentration of ROS was equal to 40 nM and the concentration of damaged mitochondria was equal to 5 nM. Time courses for ROS and Damaged Mitochondria were then simulated using Copasi.

*Model with basal ROS generation (*B2A.1-BasalROS.cps*):*

The rate of mitochondrial aging was described in the same way as in model B1:
Rate of Mitochondrial Aging=k*([HealthyMitochondria(t)]*[ROS(t)])

However, the rate of ROS generation was described by the following reaction:
Rate of ROSgeneration=ROSsynCoef*([DamagedMitochondria(t)]+kbasalROS)
Thus, even in the absence of damaged mitochondria ROS are still produced to a certain extent.

In the model B2.2-BasalROS.cps, values of ROSsynCoef and kbasalROS were calculated analytically so that initial steady state values of all variables in model B2.2-BasalROS.cps were equal to steady state values of these variables in model B1.cps (design 1).

*Model where mitochondria may get damaged also in the absence of ROS (*B2A.2-BasalMitochondrialAging.cps):

The rate of ROS generation was described in the same way as in model B1:
Rate of ROSgeneration=ROSsynCoef*([DamagedMitochondria(t)])

However, the rate of mitochondrial aging was described by the following reaction:
Rate of Mitochondrial Aging =
MitochAgingCoef*([HealthyMitochondria(t)]*[ROS(t)]+kbasalMitochAging)
Thus, even in the absence of ROS, mitochondrial damage takes place to a certain extent.

Values of MitochAgingCoef and kbasalMitochAging were calculated analytically so that initial steady state values of all variables in model B2A.2-BasalMitochondrialAging.cps were equal to steady state values of these variables in model B1.cps (design 1).

Model where mitochondria may get damaged in the absence of ROS and ROS may be produced in the absence of damaged mitochondria (Model B2A.3-BasalAll.cps*):*

The rate of mitochondrial aging was described by the following reaction:
Rate of Mitochondrial Aging=
MitochAgingCoef * ([HealthyMitochondria(t)]*[ROS(t)]+kbasalMitochAging)

The rate of ROS generation was described by the following reaction:
Rate of ROSgeneration=ROSsynCoeficient* ([DamagedMitochondria(t)]+kbasalROS)
Thus, even in the absence of ROS, mitochondrial damage takes place to a certain extent. As well, ROS are produced in the absence of damaged mitochondria.

Values of ROSsynCoef, kbasalROS, MitochAgingCoef and kbasalMitochAging were calculated analytically so that initial steady state values of all variables in model B2A.3-BasalAll.cps were equal to steady state values of these variables in model B1.cps (design 1).

Section B2B. Design 2. Model B2B.

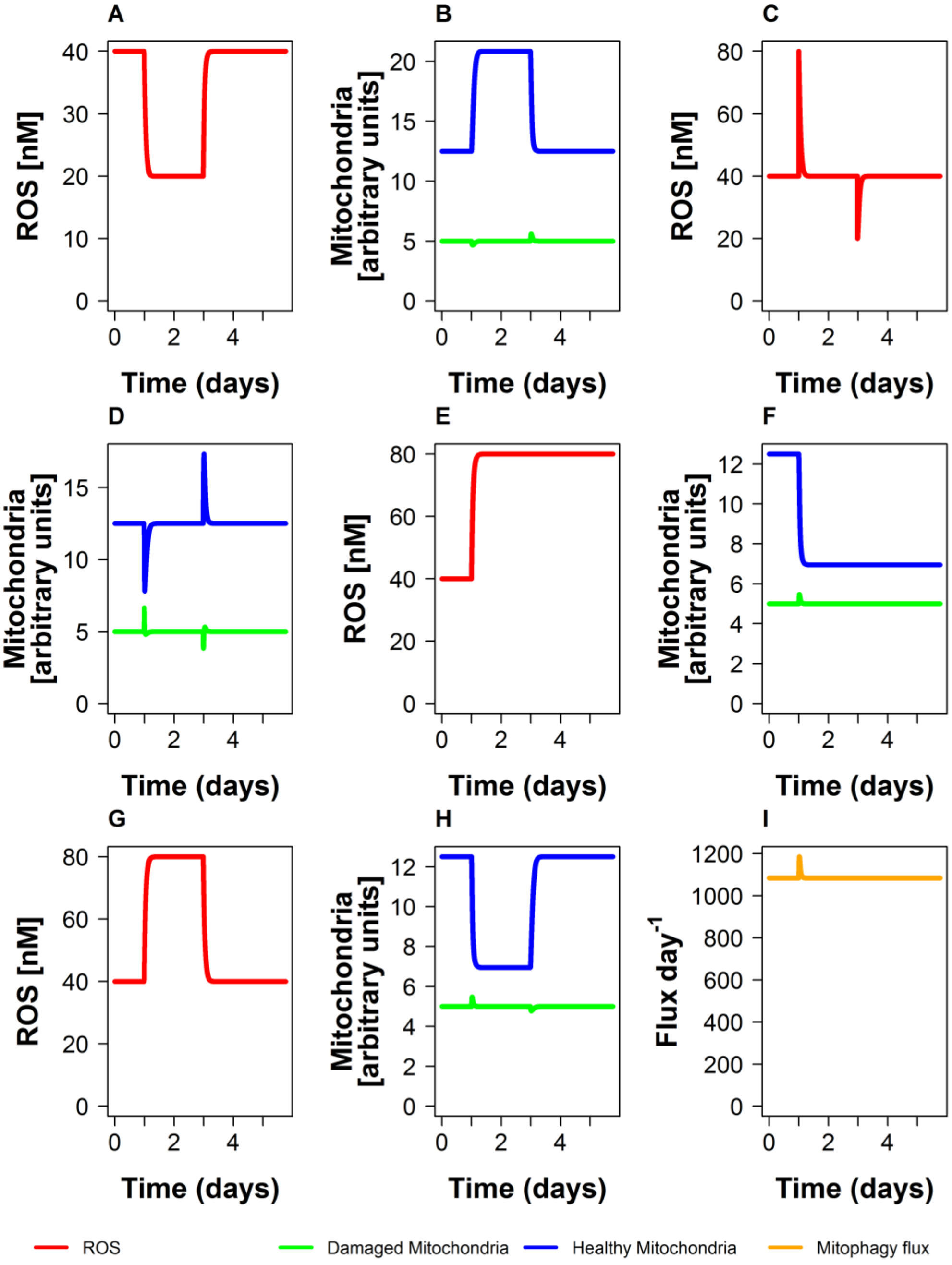

Supplementary Figure B2B.1. Design 2 (Model B2B.cps). Dynamic and structural stability obtained when the reaction of mitochondrial synthesis was added to Design 1 thereby obtaining Design 2. Concentrations of ROS (in nM) and damaged mitochondria (in arbitrary units) for design 2. Simulations used a model 2B.cps. In A and B, ROS generation rate constant was first decreased 2 fold (day 1) and then returned back (day 3). In C and D, ROS concentration

was instantaneously increased two fold (day 1) and then decreased two fold (day 3). In E, F and I the ROS generation rate constant was increased 2 fold on day 1. In G and H, ROS generation rate constant was increased 2 fold on day 1 and then decreased to the initial level on day 3.

The following conclusions were drawn from Supplementary Figure B2B.1:

A: The concentration of ROS follows the perturbation of ROS synthesis. First, ROS concentration decrease 2 fold to a lower steady value and then return back to its initial level.

B: The concentration of healthy mitochondria follows the perturbation of ROS synthesis; first the concentration of healthy mitochondria increases to a higher steady value and then return back to its initial level. The concentration of damaged mitochondria respond to perturbations, but the concentration changes are small and transient, damaged mitochondrial concentration is ultimately returned to its initial level.

C: The concentration of ROS transiently respond to perturbations, but concentration changes are transient, ROS concentration is ultimately returned to its initial level.

D: Concentrations of both healthy and damaged mitochondria transiently respond to perturbations, but both concentration changes are transient, healthy and damaged mitochondrial concentrations are ultimately returned to their initial levels.

E: ROS concentration follows the perturbation of ROS synthesis and increases 2 fold to a new steady value.

F: The concentration of healthy mitochondria follows the perturbation of ROS synthesis and decrease to a lower steady value. The concentration of damaged mitochondria responds to perturbations, but concentration changes are small and transient, damaged mitochondrial concentration is ultimately returned to its initial level.

G: ROS concentration follows the perturbation of ROS synthesis. First, ROS concentration increases 2 fold to a higher steady value and then returns back to its initial level.

H: The concentration of healthy mitochondria follows the perturbation of ROS synthesis; first, the concentration of healthy mitochondria decreases to a lower steady value and then returns back to its initial level. The concentration of damaged mitochondria responds to perturbations, but the concentration changes are small and transient, damaged mitochondrial concentration is ultimately returned to its initial level.

I. After the fluctuation at the moment of perturbation, the flux of mitophagy returns to the initial level.

We conclude that the system is both structurally and dynamically stable, for both downward and upward perturbation of ROS production.

Models building and simulations:

Model B2B and all simulations for generation of every figure have been assembled in the model archive file (SM.B2B).

The general idea used in the modelling is described below:

(re1) Mitochondria are synthesised with the constant rate. The rate constant is set in such a way that the concentration of healthy mitochondria is equal to the concentration of healthy mitochondria in models B1 and B2A.

(re2) The reaction of mitochondrial aging is catalyzed by ROS with the rate described by mass action kinetics: Rate of Mitochondrial Aging=k*([HealthyMitochondria(t)]*[ROS(t)]). Thus, in the absence of ROS, mitochondria were not damaged.

(re3) The rate of removal of impaired mitochondria (mitophagy) is modelled with irreversible mass action kinetics:
Rate of Mitophagy = k*[Impaired mitochondria(t)]*[parkin(t)] *[p62(t)]

(re4) The reaction of ROS generation is catalyzed by impaired mitochondria with the rate described by mass action kinetics:

Rate of ROSgeneration=ROSsynCoef*([DamagedMitochondria(t)]). Thus, in the absence of damaged mitochondria ROS are not produced.

(re5) ROS removal is modelled as irreversible mass action reaction:
Rate of ROS removal = k*[ROS(t)]*[Antiox(t)]

Antiox was included in the reaction as catalyst (i.e. without being consumed) because we considered not only antioxidant metabolites (e.g. various antioxidants which the cell would maintain at constant concentrations), but also enzymes (e.g. superoxide dismutase) catalyzing ROS removal. P62 and parkin are consumed for mitophagy as substrates. Antiox, p62 and parkin concentrations are taken constant, i.e. independent of time. Simulations started with initial conditions where the concentration of ROS was equal to 40 nM and the concentration of damaged mitochondria was equal to 5 nM. Time courses for ROS and Damaged Mitochondria were then simulated using Copasi.

Section B3. Design 3. Model B3.

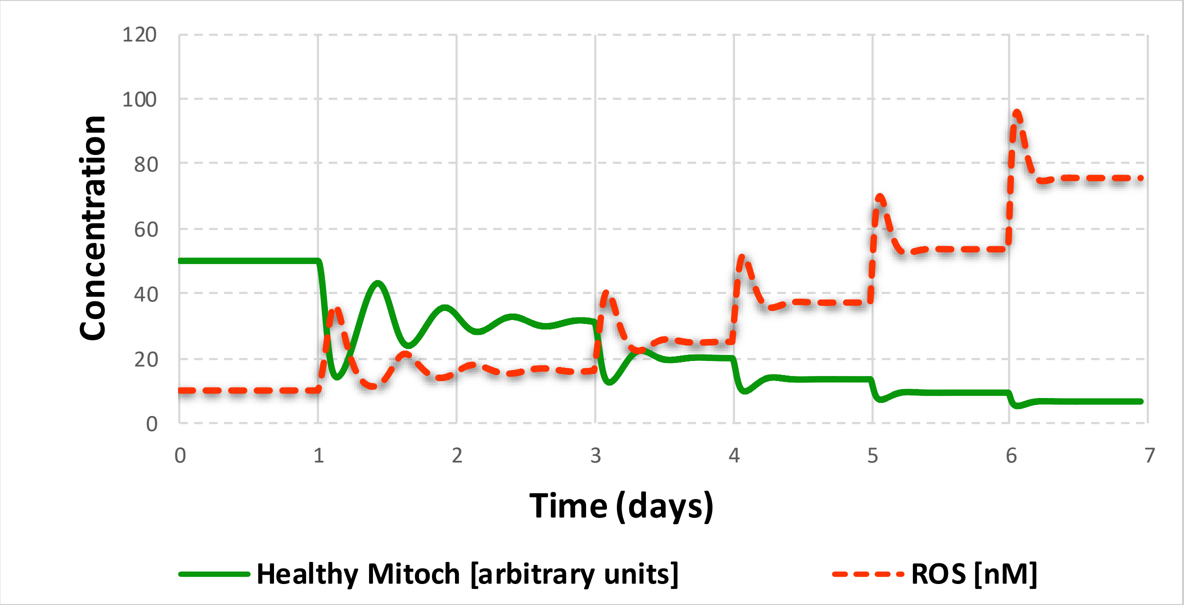

Supplementary Figure B3.1. Design 3 (Model 3.cps). Homeostasis obtained when the negative feedback loop was added to design 2 thereby obtaining design 3. Concentrations of ROS (in nM) and healthy mitochondria (in arbitrary units) for design 3. Simulations used a model 3.cps. ROS synthesis was doubled stepwise: day 1-ROS synthesis was doubled comparing to initial steady state (2 fold increase from initial value); day 3 – ROS synthesis was doubled comparing to day 1 (4 fold increase from initial value); day 4 – ROS synthesis was doubled comparing to day 3 (8 fold up from initial value); Day 5 – ROS synthesis was doubled comparing to day 4 (16 fold up from initial value); Day 6 – ROS synthesis was doubled comparing to day 5 (32 fold up from initial value).

The following conclusions were drawn from Figure SMB3.1:

In the response to each perturbation, concentrations of ROS and healthy mitochondria transiently reaches new steady state. Upon every doubling of ROS synthesis, ROS concentration is first increased twice, but then decreases back to the value a bit higher than the initial one. Upon every perturbation, the concentration of healthy mitochondria first decreases 2 fold, but then increases back to the value a bit lower than the steady state value before perturbation. We conclude that the system actively counteracts the increase of ROS.

A

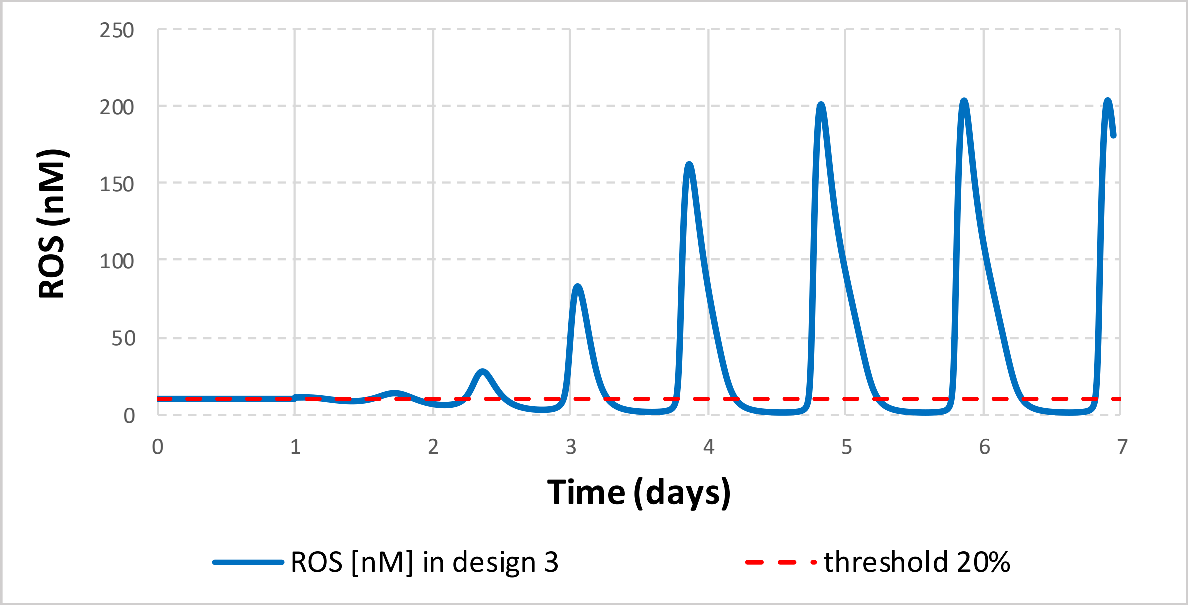

B

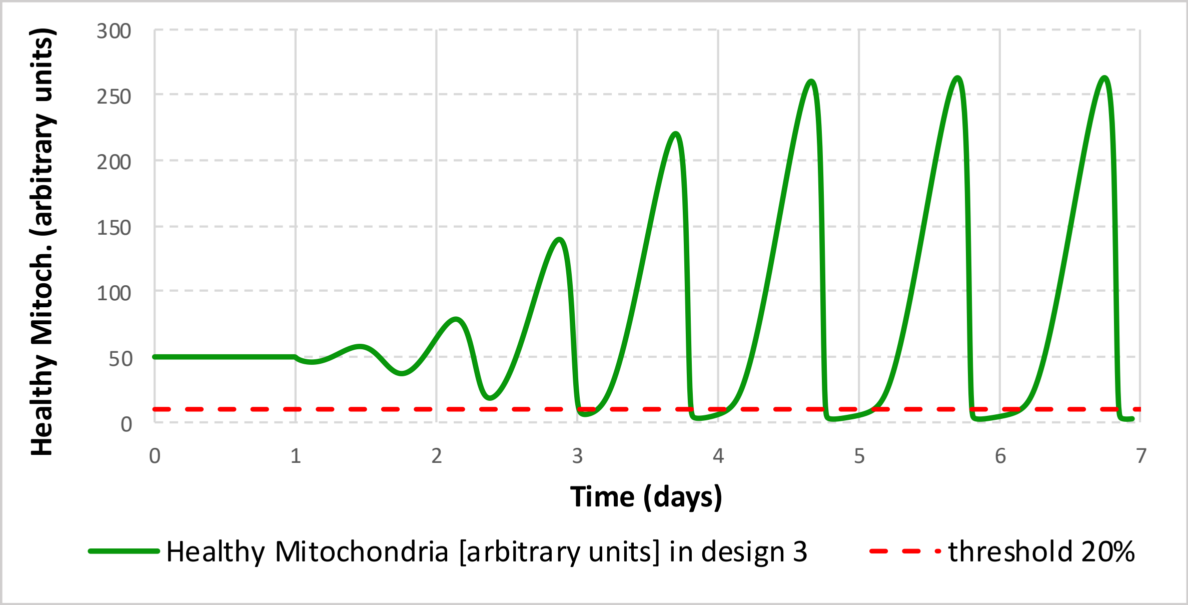

Supplementary Figure B3.2. Design 3 (Model 3.cps). Compromised dynamic stability in homeostatic design 3. Concentrations of (A) ROS (in nM) and (B) of healthy mitochondria (in arbitrary units) for design 3. Simulations used a model B3.cps. The initial ROS concentration was perturbed (transient increase from 10 nM to 11 nM) at day 1.

The following conclusions were drawn from Supplementary Figure B3.2:

A: The increase of ROS concentration from 10 nM t 11 nM at day 1 triggers an oscillatory behaviour of the ROS concentration.

B: The increase of ROS concentration from 10 nM to 11 nM at day 1 triggers an oscillatory behaviour of the ROS concentration. During oscillations, the concentration of healthy mitochondria sweeps below a hypothetical viability that corresponds to the threshold line (marked as a dotted red line) dissecting 20% (10 a.u) of the initial concentration of healthy mitochondria (50 a.u).

We conclude that the system is dynamically unstable against the perturbation of ROS concentration.

Model building and simulations

Model B3 and all simulations for generation of every figure have been assembled in the model archive file (SM.B3).

The general idea used in the modelling is described below:

The reactions of synthesis and degradation of Antiox (re8 and re9), p62 (re6 and re7) and parkin (re10 and re11) were added to design 2 (see Supplementary Information SM.B2B) and set in the way to reach steady state concentrations of Antiox, p62 and parkin equal to their steady state concentrations in model B2B. A negative ROS-regulated feedback loop was added. ROS catalyze reaction 12 and shift the equilibrium towards a higher rate of keap1 oxidation (deactivation). Keap1 catalyzes reaction 14 and shifts the equilibrium towards the higher fraction of inactive Nrf2. Thus, ROS deactivate keap1 and activate Nrf2. When active, Nrf2 activates the expression of p62 (reaction 6) and the antioxidant response (reaction 8). The increase of ROS concentration activates ROS removal in antioxidant response, as well as p62-mediated removal of damaged mitochondria.

Computations were performed in ds. Comparing with previous models (B1, B2A and B2B) the ROS generation rate constant was decreased 4 fold to start simulations from the steady state with higher concentration of healthy mitochondria and lower concentration of damaged mitochondria and ROS (because in the following experiments the ROS generation rate constant increased several folds). Perturbations were performed using “time event” function in COPASI. The “factor time” was accumulating in time. At the moment “factor time” reached a certain level, the following events were triggered for Figure SMB3.1:
day 1 (factor time =10)-ROS synthesis is doubled (2 fold up from initial value);
day 3 (factor time =30)-ROS synthesis is doubled (4 fold up from initial value);
day 4 (factor time =40)-ROS synthesis is doubled (8 fold up from initial value);
day 5 (factor time =50)-ROS synthesis is doubled (16 fold up from initial value);
day 6 (factor time =60)- ROS synthesis is doubled (32 fold up from initial value).

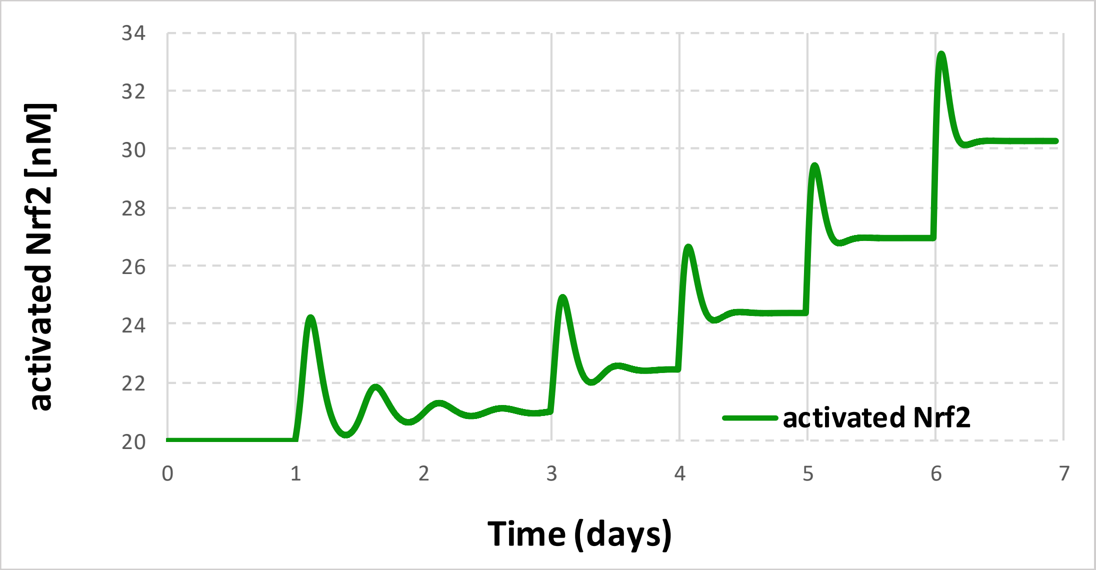

Supplementary Figure B3.3. Design 3 (Model 3.cps). Oscillations of activated Nrf2. The concentration of Nrf2 (in nM) was simulated. ROS synthesis was doubled stepwise: day 1-ROS synthesis was doubled comparing to initial steady state (2 fold increase from initial value); day 3 – ROS synthesis was doubled comparing to day 1 (4 fold increase from initial value); day 4 – ROS synthesis was doubled comparing to day 3 (8 fold up from initial value); Day 5 – ROS synthesis was doubled comparing to day 4 (16 fold up from initial value); Day 6 – ROS synthesis was doubled comparing to day 5 (32 fold up from initial value).

The following conclusions were drawn from Figure SMB3.3:

In the response to each perturbation, concentrations of activated Nrf2 transiently reaches a new steady state. At some range of the ROS generation rate constant, Nrf2 transiently oscillates.

Section B4. Design 4. Model B4.

A

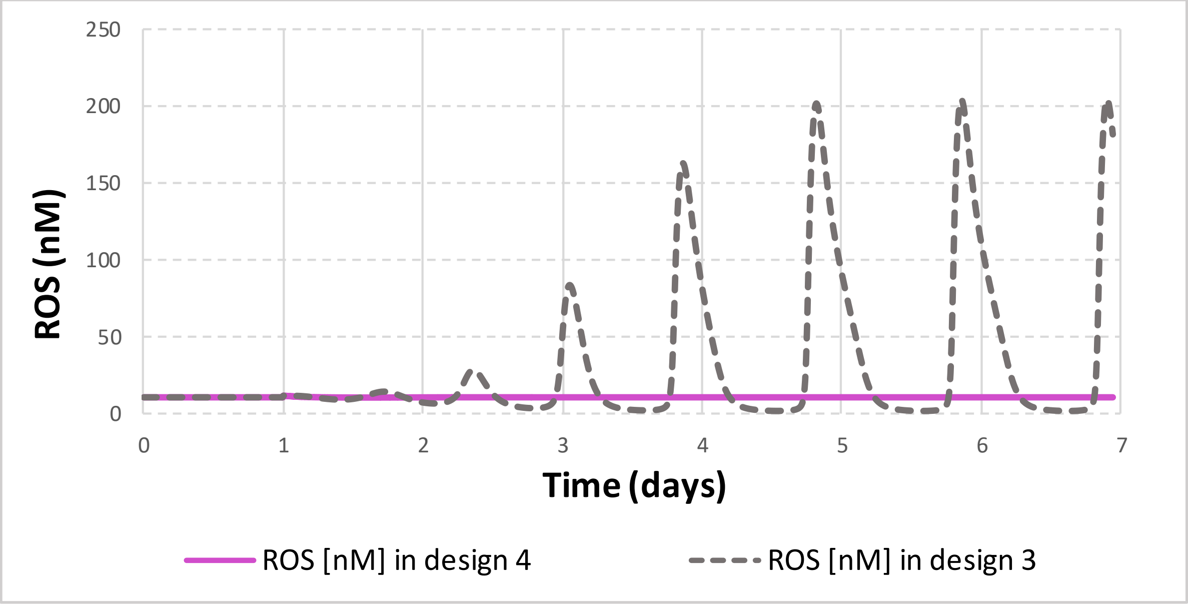

B

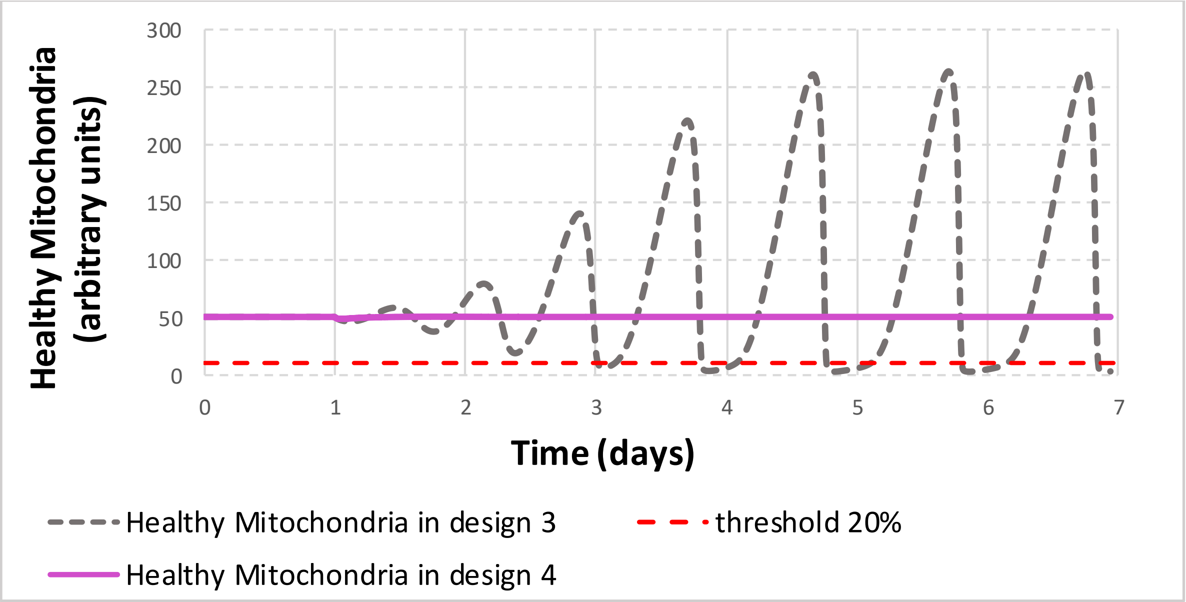

Supplementary Figure B4.1. Design 4 (Model B4.cps). Dynamic stability obtained when NFκB signalling was added to design 3 thereby obtaining design 4. Concentrations of (A) ROS (in nM) and (B) of healthy mitochondria (in arbitrary units) for design 4 (solid purple line) comparing with design 3 (dashed grey line). Simulations used models 3.cps and 4.cps. The Initial ROS concentration was perturbed (transient increase from 10 nM to 11 nM) at day 1.

The following conclusions were drawn from Supplementary Figure B4.1:

A: In design 3, the increase of ROS concentration from 10 nM to 11 nM at day 1 triggers an oscillatory behaviour of the ROS concentration. On the contrary, in design 4, ROS concentration increases transiently, but then quickly returns to the initial value.

B. In design 3, the increase of ROS concentration from 10 nM to 11 nM at day 1 triggers an oscillatory behaviour of ROS concentration. During oscillations, the concentration of healthy mitochondria sweeps below a hypothetical viability that corresponds to the threshold line (marked as a dotted red line) dissecting 20% (10 nM) of the initial concentration of healthy mitochondria (50 nM). On the contrary, in design 4, immediately upon perturbation, the concentration of healthy mitochondria drops from 50 nM to 48 nM, but then quickly returns to the initial value (50 nM) and does not sweep below a viability line (threshold line of 10 nM, shown as a dotted red line that dissects 20% of the initial concentration of healthy mitochondria).

We conclude that design 4 (comparing with design 3) gains dynamical stability against the perturbation of ROS concentration.

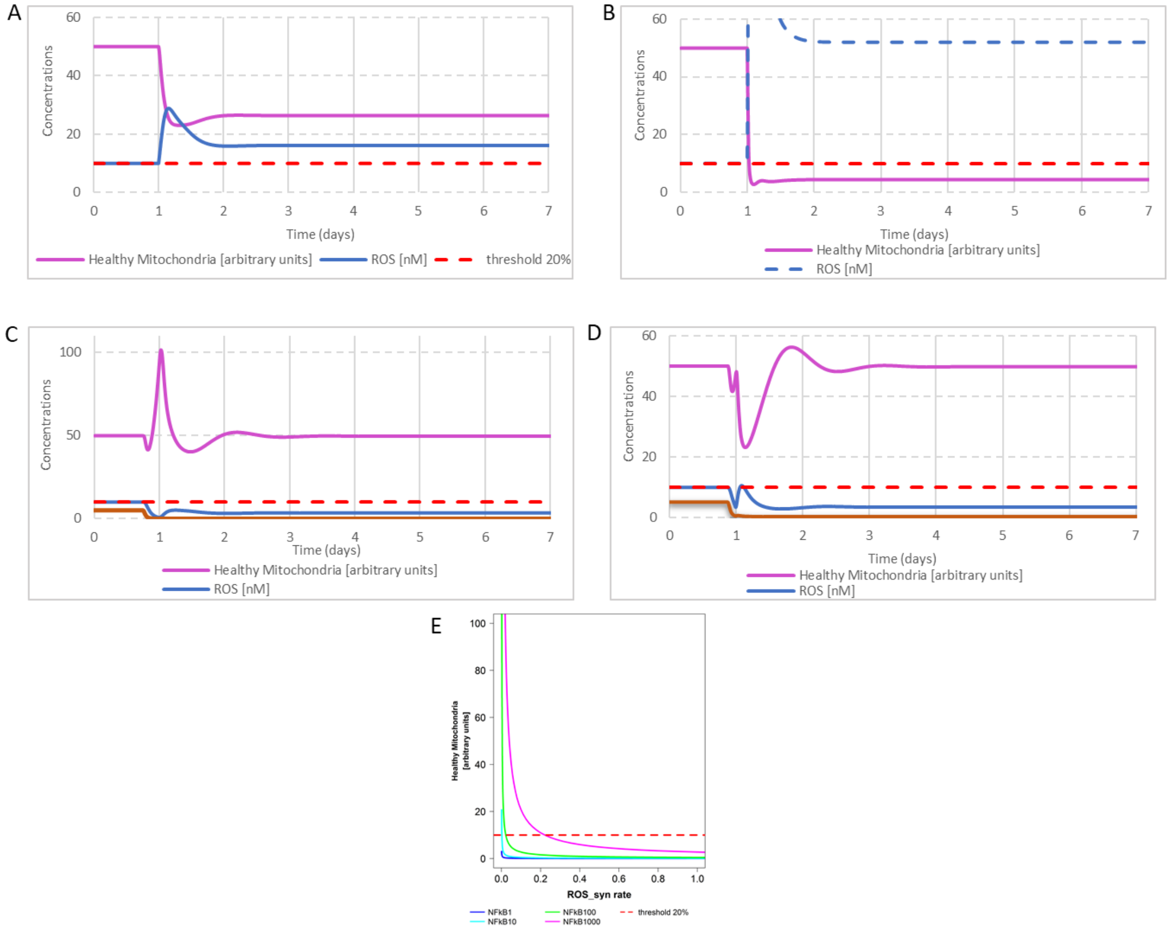

Supplementary Figure B4.2. Design 4 (Model B4.cps). NFκB signalling protects from the increase of ROS generation in design 4. Concentrations of ROS (in nM) and of healthy and damaged mitochondria (in arbitrary units) for design 4. Simulations used a model 4.cps. In A the

ROS generation rate constant was increased 2 fold on day 1. In B the ROS generation rate constant was increased 15 fold on day 1. In C the ROS generation rate constant was increased 15 fold on day 1, but, 6h before the increase of ROS generation, the NFκB signalling was increased 15 fold. In D the ROS generation rate constant was increased 15 fold on day 1, but, 3h before the increase of ROS generation, the NFκB signalling was increased 15 fold. In E the steady state concentrations of healthy mitochondria (the ordinate) are shown for different rates of ROS generation (the abscissa) for 4 levels of NFκB signalling: (i) activated 1 fold (dark blue line); (ii) activated 10 fold (light blue line); (iii) activated 100 fold (green line); (iv) activated 1000 fold (purple line).

The following conclusions were drawn from Figure SMB4.2:

A: The concentration of ROS first increases, but then decreases and reaches the new steady state, with the level around 30% higher than the initial one. The concentration of healthy mitochondria decreases around 2 fold and persists at the new steady state level.

B: The concentration of healthy mitochondria drops below a viability line (the threshold line of 10 nM shown as a dotted red line that dissects 20% of the initial concentration of healthy mitochondria).

C: When NFκB signalling is increased, the damaged mitochondria (brown line) are converted into healthy mitochondria (purple line). The concentration of damaged mitochondria decreases. Thus, ROS concentration decreases as well. The increase of ROS synthesis stops this growth. ROS concentration increases again and the concentration of healthy mitochondria drops again. However, the concentration of healthy mitochondria does not drop below a viability line (the threshold line of 10 nM shown as a dotted red line that dissects 20% of the initial concentration of healthy mitochondria).

D: When NFκB signalling is increased, the damaged mitochondria (brown line) are converted into healthy mitochondria (purple line). The concentration of damaged mitochondria decreases. Thus, ROS concentration decreases as well. The increase of ROS synthesis stops this growth. ROS concentration increases again and the concentration of healthy mitochondria drops again. This dynamics is similar to the one described in C. However, the reduced time gap between the activation of NFκB signalling and the increase of ROS generation does not allow to achieve high peak of the concentration of healthy mitochondria. Nevertheless, at the end all concentrations reaches the same steady state values as in C. The concentration of healthy mitochondria does not drop below a viability line (the threshold line of 10 nM shown as a dotted red line that dissects 20% of the initial concentration of healthy mitochondria).

E. The higher is ROS generation, the lower is the steady state concentration of healthy mitochondria. The higher is NFκB signalling, the higher is the concentration of healthy mitochondria.

We conclude that the NFκB signalling helps to protect against the increase of ROS generation.

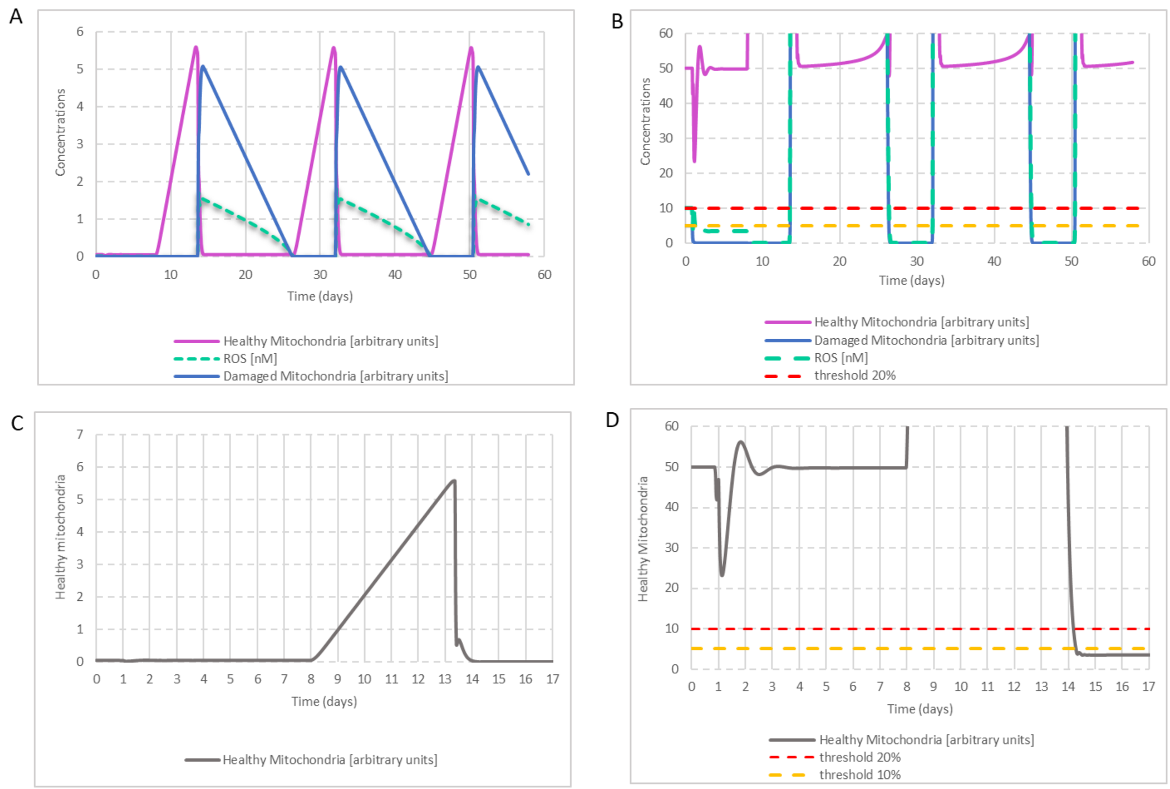

Supplementary Figure B4.3. Design 4 (Model B4.cps). A potential danger of the NFκB-mediated accumulation of healthy mitochondria. Concentrations of ROS (in nM) and of healthy and damaged mitochondria (in arbitrary units) for design 4. Simulations used a model 4.cps.
In A (bird-eye view) and B (high resolution view) the ROS generation rate constant was increased 15 fold on day 1, but, 3h before the increase of ROS generation, the NFκB signalling was increased 15 fold (the same perturbation as on Supplementary Figure B4.2D). When the system reached a steady state, the ROS generation rate constant was decreased 15 fold (on day 8) and oscillations started. In C (bird-eye view) and D (high resolution view): when a system was in an oscillatory mode (shown in A and B), the ROS generation rate constant was increased for the second time (on day 13.3). This was a time point when the concentration of healthy mitochondria was near its peak value.

The following conclusions were drawn from Supplementary Figure B4.3:

A: Upon the decrease of the ROS generation rate constant the system enters the oscillatory mode.

B: During the oscillation, the concentration of healthy mitochondria goes up and down the initial steady state value and does not sweep below a viability line (a threshold line of 10 nM shown as dotted red line that dissects 20% of the initial concentration of healthy mitochondria).

C: When the ROS generation rate constant is increased for the second time, the concentration of healthy mitochondria quickly goes down and oscillations stop.

D: When the concentration of healthy mitochondria goes down, it sweeps below a viability line (a threshold line of 10 nM shown as dotted red line that dissects 20% of the initial concentration of healthy mitochondria). We can note that the initial concentration of healthy mitochondria was at 50 nM. When the ROS generation rate constant is decreased, the concentration of healthy

mitochondria goes up. However, when the ROS generation rate constant is increased for the second time, the concentration of healthy mitochondria sweeps much below the initial value (50 nM).

We conclude that a very high accumulation of healthy mitochondria creates a potential danger for the collapse when the ROS generation rate constant is increased for the second time. Design 4 is unstable against the second pulse of the increased ROS generation rate constant.

Model building and simulations:

Model B4 and all simulations for generation of every figure have been assembled in the model archive file (SM.B4).

The general idea used in the modelling is described below:

Model B4 was built by adding 5 additional reactions (with mass action kinetics) to model B3:

(re16): parkin activates NFκB signaling via IKK

(re17): the removal of NFκB signal

(re18): NFκB activates the expression of Bclxl

(re19): the degradation of Bclxl

(re20): Bclxl activates the recovery of mitochondria via biosynthesis.

The rate law of reaction 6 was modified in such a way that NFκB signal could activate p62 transcription: v_6= kf * NrfAct(t)*NFκB(t)*S, kf was fitted to reach steady state concentration of p62 equal to its concentration in model B3.

The rate constant of reaction 2 was adjusted in such a way that steady state concentrations of healthy and damaged mitochondria were equal to their steady state concentrations in model B3.

Computations were performed in ds, changes are shown in the scale of days. Perturbations were performed using "time event" function in COPASI.

Computations were performed in ds. Perturbations were performed using "time event" function in COPASI. The "factor time" was accumulating in time. At the moment "factor time" reached a certain level, the events were triggered.

Section B5. Design 5. Models B5.1-B5.3A.

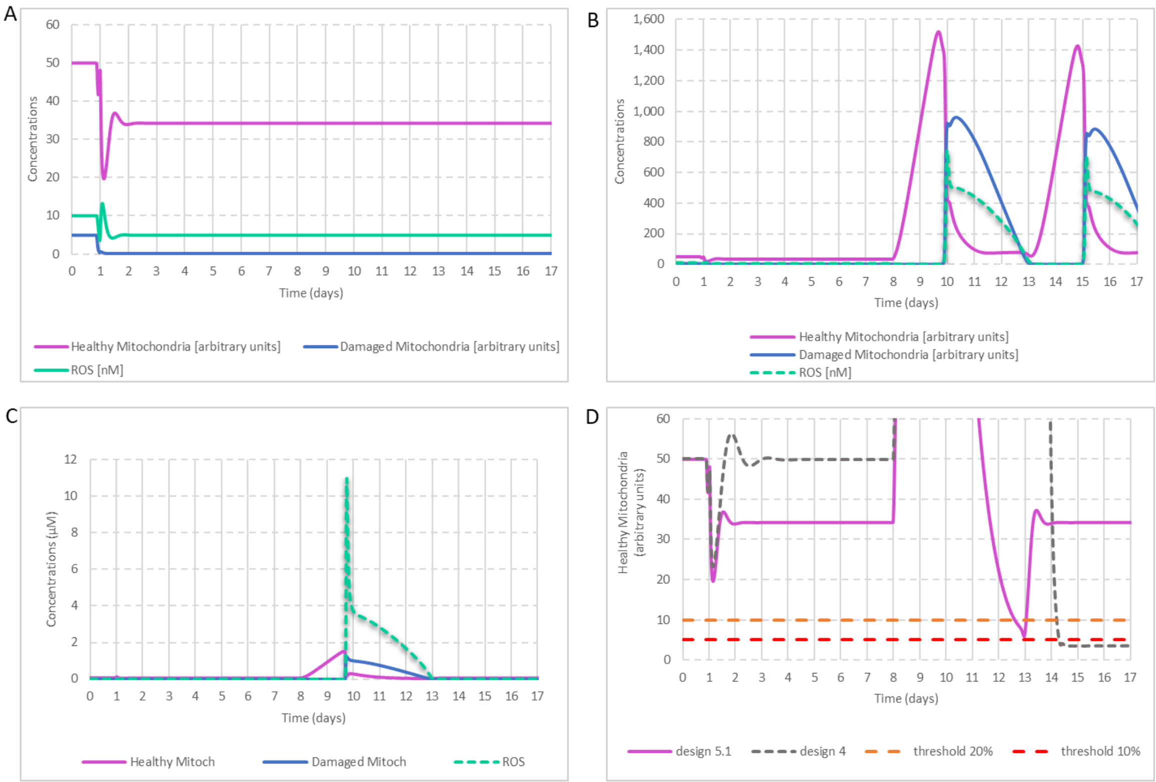

Supplementary Figure B5.1. Design 5.1 (Model B5.1.cps). Robustness vis-à-vis with respect to the second pulse of ROS obtained when the regulation of NFkB signalling by ROS via DJ1 was added to design 4 thereby obtaining design 5.1. Concentrations of ROS (in nM) and of healthy and damaged mitochondria (in arbitrary units) for design 5.1 (A-D) and design 4 (D). In A-D simulations model B5.1.cps was used. In D simulations model B5.1.cps was compared with model B4.cps.

In A, B, C and D the ROS generation rate constant was increased 15 fold on day 1, but, 3 hours before the increase of ROS generation, the NFκB signalling was increased 15 fold (the same perturbation as on Figures SM.B4.2D and SMB4.3). In A, the system reached a new steady state and no further perturbations were applied. In B, C and D the initial perturbations were followed by the decrease of ROS generation rate constant 15 fold on day 8. In C (bird-eye view) and D (high resolution view), when a system was in an oscillatory mode, the ROS generation rate constant was increased for the second time on day 9.8, at the time point when the concentration of healthy mitochondria was near its peak value.

The following conclusions were drawn from Supplementary Figure B5.1:

A: The system reaches a new steady state with the lower value of healthy mitochondria.

B: Upon the decrease of the ROS generation rate constant the system enters the oscillatory mode.

C: When the ROS generation rate constant is increased for the second time, the concentration of healthy mitochondria quickly goes down and oscillations stop.

D: In both model B4.cps (design 4) and model B5.1.cps (design 5.1) the concentration of healthy mitochondria sweeps below a viability line (a threshold line of 10 a.u. shown as a dotted red line that dissects 20% of the initial concentration of healthy mitochondria). However, in model B5.1.cps (design 5.1) the concentration of healthy mitochondria sweeps below a viability line only transiently and quickly recovers back to the initial level.

We conclude that the regulation of NFκB signalling by ROS via DJ1 provides limited robustness vis-à-vis with respect to the second pulse of ROS.

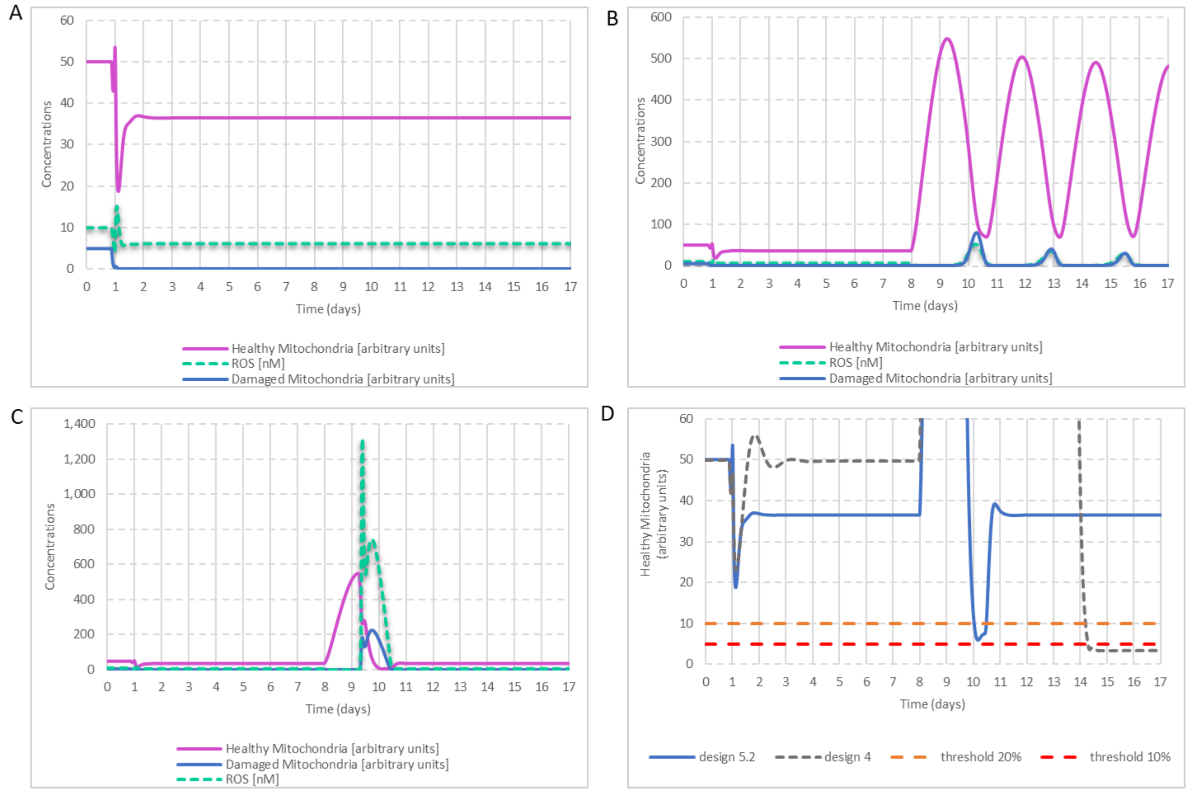

Supplementary Figure B5.2. Design 5.2 (Model B5.2.cps). Robustness vis-à-vis with respect to the second pulse of ROS obtained when the regulation of Nrf2 signalling by ROS via DJ1 was added to design 4 thereby obtaining design 5.2. Concentrations of ROS (in nM) and of healthy and damaged mitochondria (in arbitrary units) for design 5.2 (A-D) and design 4 (D). In A-D simulations model B5.2.cps was used. In D simulations model B5.2.cps was compared with model B4.cps.

In A, B, C and D the ROS generation rate constant was increased 15 fold on day 1, but, 3h before the increase of ROS generation, the NFκB signalling was increased 15 fold (the same perturbation as on Figures SM.B4.2D, SM.B4.3 and SM.B5.1). In A, the system reached a new steady state and no further perturbations have been made. In B, C and D the initial perturbations were followed by the decrease of ROS generation rate constant 15 fold on day 8. In C (bird-eye view) and D (high resolution view), when a system was in an oscillatory mode, the ROS generation rate constant was increased for the second time on day 9.3, at the time point when the concentration of healthy mitochondria was near its peak value.

The following conclusions were drawn from Supplementary Figure B5.2:

A: The system reaches a new steady state with the lower value of healthy mitochondria.

B: Upon the decrease of the ROS generation rate constant the system enters the oscillatory mode.

C: When the ROS generation rate constant is increased for the second time, the concentration of healthy mitochondria quickly goes down and oscillations stop.

D: In both models B4.cps (design 4) and model B5.2.cps (design 5.2) the concentration of healthy mitochondria sweeps below a viability line (a threshold line of 10 a.u. shown as a dotted red line that dissects 20% of the initial concentration of healthy mitochondria). However, in model B5.2.cps (design 5.2) the concentration of healthy mitochondria sweeps below a viability line only transiently and quickly recovers back to the initial level.

We conclude that the regulation of Nrf2 signalling by DJ1 provides limited robustness vis-à-vis with respect to the second pulse of ROS.

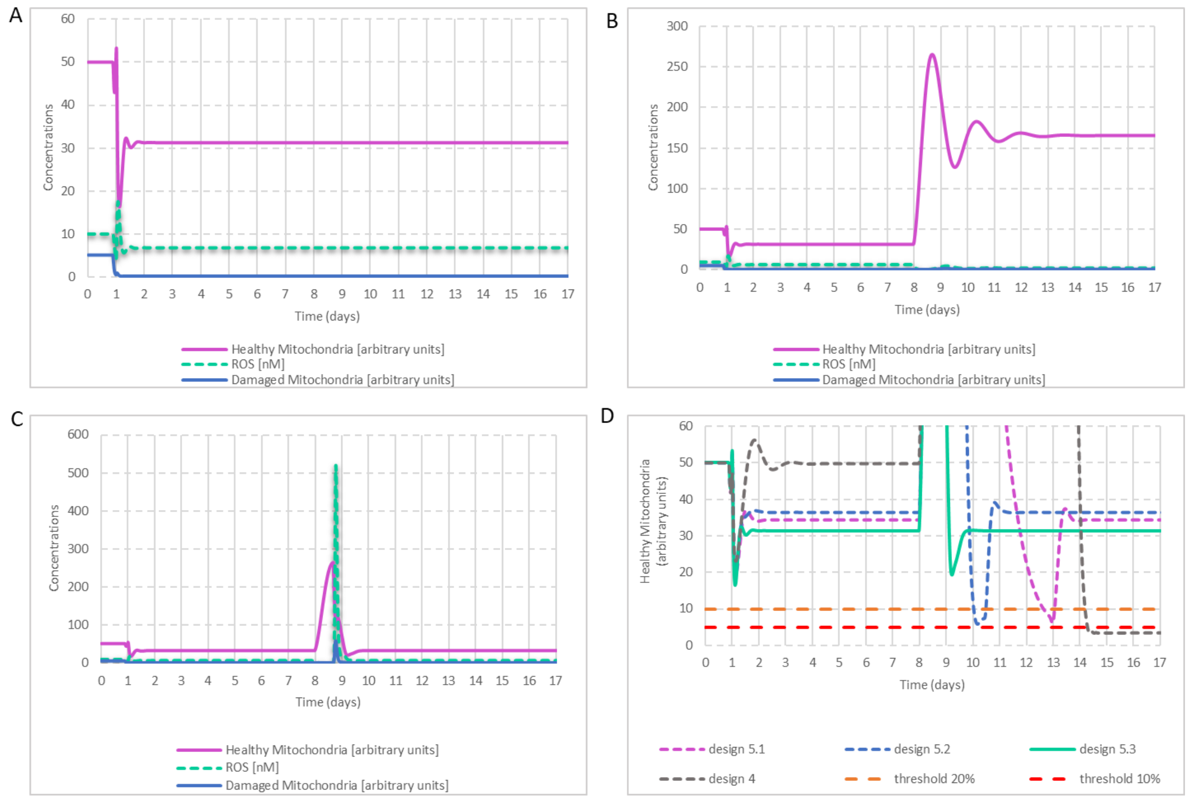

Supplementary Figure B5.3. Design 5.3 (Model B5.3.cps). Robustness vis-à-vis with respect to the second pulse of ROS obtained when the regulation of both NFκB and Nrf2 signalling by DJ1 was added to design 4 thereby obtaining design 5.3. Concentrations of ROS (in nM) and of healthy and damaged mitochondria (in arbitrary units) for design 5.3 (A-D) and design 4 (D). In A-D simulations model B5.3.cps was used. In D model B5.2.cps was compared with models B4.cps, B5.1.cps and B5.3.cps.

In A, B, C and D the ROS generation rate constant was increased 15 fold on day 1, but, 3h before the increase of ROS generation, the NFκB signalling was increased 15 fold (the same perturbation as on Figures SM.B4.2D, SM.B4.3, SM.B5.1 and SM.B5.2). In A, the system reached a new steady

state and no further perturbations have been made. In B, C and D the initial perturbations were followed by the decrease of ROS generation rate constant 15 fold on day 8. In C (bird-eye view) and D (high resolution view), when a system was in an oscillatory mode, the ROS generation rate constant was increased for the second time on day 8.7, at the time point when the concentration of healthy mitochondria was near its peak value.

The following conclusions were drawn from Supplementary Figure B5.3:

A: The system reaches a new steady state with the lower value of healthy mitochondria (31 a.u.).

B: Upon the decrease of the ROS generation rate constant the system exhibites several dumping oscillations and reaches a new steady state with the higher concentration of healthy mitochondria (165 a.u.).

C: When the ROS generation rate constant is increased for the second time, the concentration of healthy mitochondria quickly goes down to the steady state value of 35 a.u.

D: In models B4.cps, B5.1.cps and B5.2.cps the concentration of healthy mitochondria sweeps below a viability line (a threshold line of 10 a.u. shown as a dotted red line that dissects 20% of the initial concentration of healthy mitochondria). However, in model B5.3.cps (design 5.3) the concentration of healthy mitochondria does not sweep below a viability line.

We conclude that the regulation of both NFκB and Nrf2 signalling by ROS via DJ1 provides robustness vis-à-vis with respect to the second pulse of ROS.

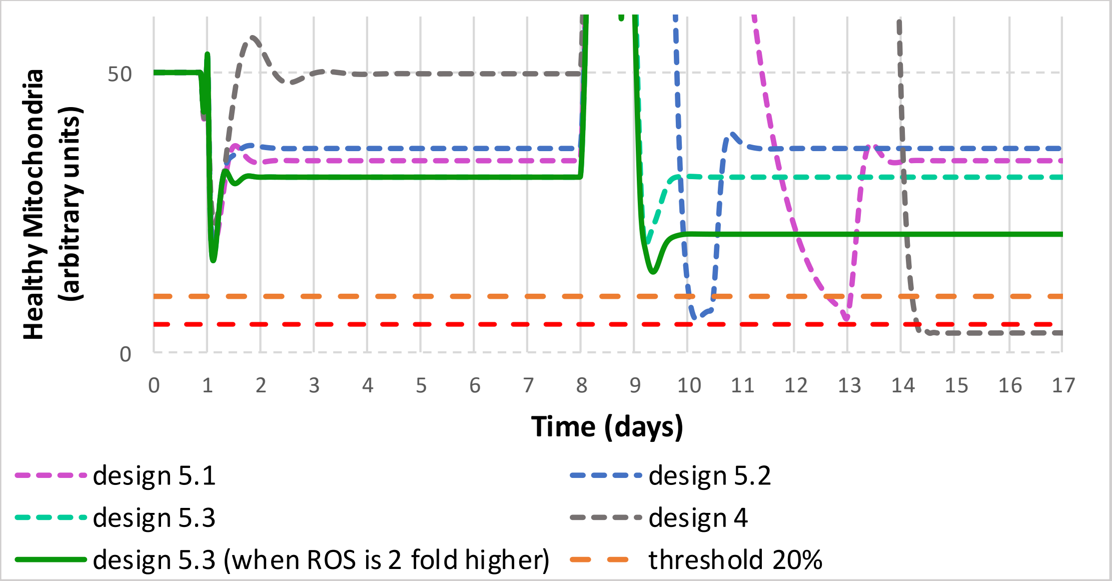

Supplementary Figure B5.4. Design 5.3 (Model B5.3.cps). Robustness vis-à-vis with respect to the higher second pulse of ROS. Concentrations of ROS (in nM) and of healthy and damaged mitochondria (in arbitrary units). Models B4, B5.1, B5.2 and B5.3.cps were compared.
The ROS generation rate constant was increased 15 fold on day 1, but, 3h before the increase of ROS generation, the NFκB signalling was increased 15 fold (the same perturbation as on Figures SM.B4.2D, SM.B4.3, SM.B5.1, SM.B5.2 and SM.B5.3). The initial perturbations were followed

(on day 8) by the decrease of ROS generation rate constant 15 fold that resulted in the increase of the concentration of healthy mitochondria. At the time point when the concentration of healthy mitochondria was near its peak value, in each model, the ROS generation rate constant was increased for the second time either 15 fold (Models B4-dotted grey line; B5.1-dotted purple line; B5.2-dotted green line; B5.3-solid light green line), or 30 fold (Model B.3-dark green line).

The following conclusions were drawn from Supplementary Figure B5.4:

In models B4 (design 4), B5.1 (design 5.1), and B5.2 (design 5.2) the concentration of healthy mitochondria sweeps below a viability line (a threshold line of 10 a.u. shown as a dotted red line that dissects 20% of the initial concentration of healthy mitochondria). However, in model B5.3 (design 5.3) the concentration of healthy mitochondria does not sweep below a viability line, even if the ROS generation rate constant is increased 30 instead of 15 fold.

We conclude that the regulation of both NFκB and Nrf2 signalling by ROS via DJ1 (Model 5.3, design 5.3) provides strong robustness vis-à-vis with respect to the second pulse of ROS, even for the higher ROS challenge.

A

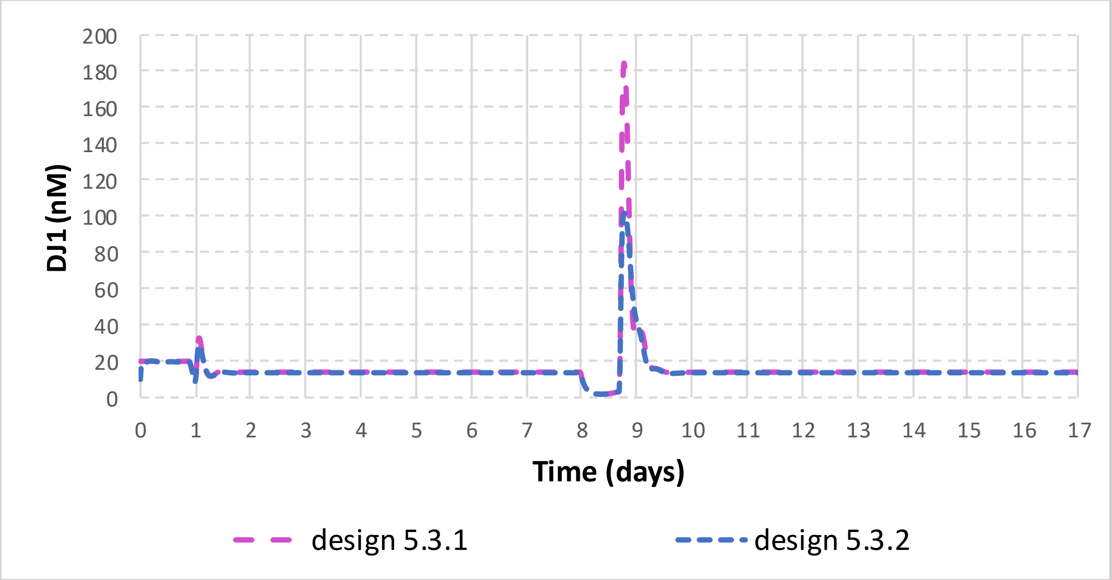

B

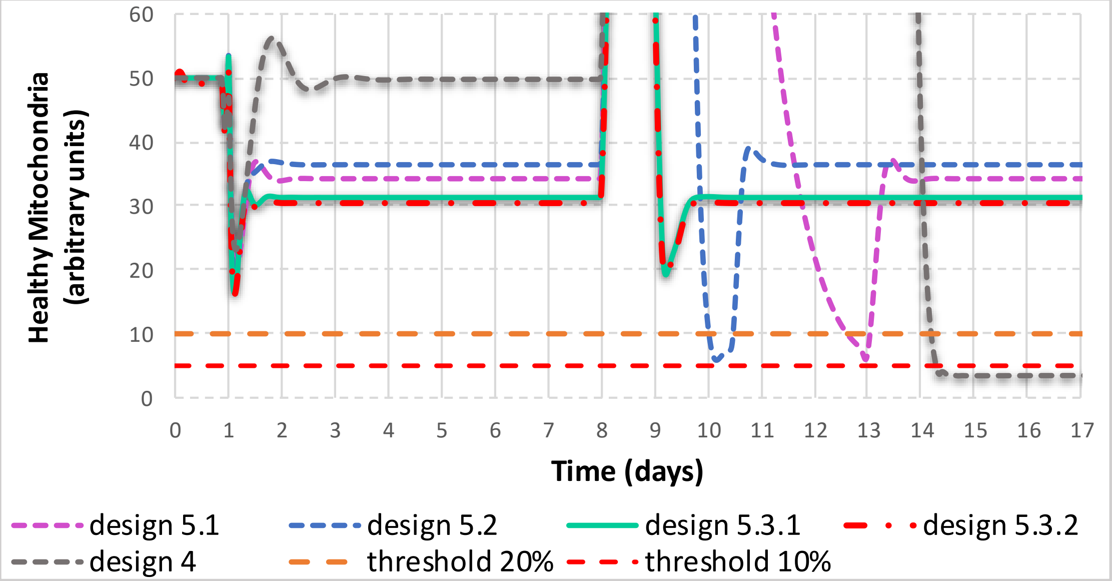

Supplementary Figure B5.5. Design 5.3 (intact models B5.3.1.cps and the model with reduced DJ1 activity B5.3.2.cps) comparing with design 4 (model B4.cps), design 5.1 (model B5.1.cps) and design 5.2 (model B5.2.cps). Concentrations of ROS (in nM) and of healthy and damaged mitochondria (in arbitrary units). In A the activity of DJ1 in the intact model (model B5.3.1) and in the model with reduced DJ1 activity (model B5.3.2.cps) is shown. In B, the concentration of healthy mitochondria for models B.4.cps, B5.1.cps, B5.2.cps, B5.3.1.cps and B5.3.2.cps (with reduced activity of DJ1) is shown. The ROS generation rate constant was increased 15 fold on day 1, but, 3h before the increase of ROS generation, the NFκB signalling was increased 15 fold (the same perturbation as on Figures SM.B4.2D, SM.B4.3, SM.B5.1, SM.B5.2, SM.B5.3 and SM.B5.4). The initial perturbations were followed (on day 8) by the decrease of ROS generation rate constant 15 fold that resulted in the increase of the concentration of healthy mitochondria. At the time point when the concentration of healthy mitochondria was near its peak value, in each model, the ROS generation rate constant was increased for the second time 15 fold.

The following conclusions were drawn from Supplementary Figure B5.5:

A: The response in DJ1 activation in model 5.3.1.cps is twice lower than in the model 5.3.2.cps.

B: In models B4 (design 4), B5.1 (design 5.1), and B5.2 (design 5.2) the concentration of healthy mitochondria sweeps below a viability line (a threshold line of 10 a.u. shown as a dotted red line that dissects 20% of the initial concentration of healthy mitochondria). However, in model B5.3 (design 5.3), for both intact DJ1 activity (model B5.3.1.cps) and for model with the reduced DJ1 activity (model B5.3.2) the concentration of healthy mitochondria does not sweep below a viability line.

We conclude that the regulation of both NFκB and Nrf2 signalling by ROS via DJ1 (design 5.3) provides strong robustness vis-à-vis with respect to the second pulse of ROS, even for lower DJ1 activity.

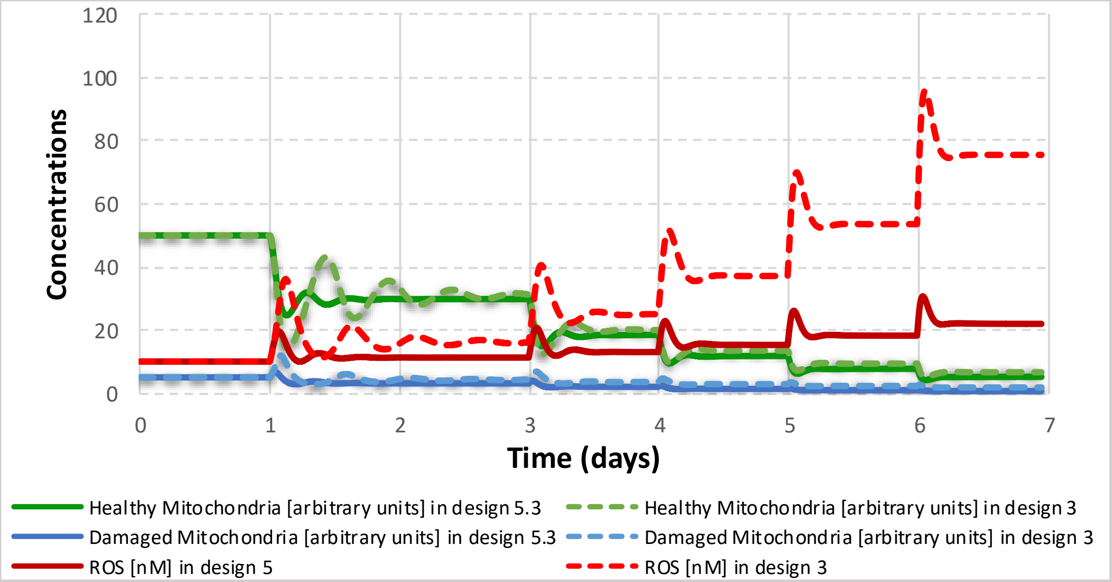

Supplementary Figure B5.6. Design 5.3 comparing with design 3 (model B3.cps, Supplementary Figure B3.1).

Strong homeostasis obtained in design 5 where both NFκB and Nrf2 loops were sensitive to DJ1. Concentrations of ROS (in nM) and healthy and damaged mitochondria (in arbitrary units) for design 3 and design 5. Simulations used a model B3.cps and B5.3.cps. ROS synthesis was doubled stepwise: day 1-ROS synthesis was doubled comparing to initial steady state (2 fold increase from initial value); day 3 – ROS synthesis was doubled comparing to day 1 (4 fold increase from initial value); day 4 – ROS synthesis was doubled comparing to day 3 (8 fold up from initial value); Day 5 – ROS synthesis was doubled comparing to day 4 (16 fold up from initial value); Day 6 – ROS synthesis was doubled comparing to day 5 (32 fold up from initial value).

The following conclusions were drawn from Figure SMB5.6:

In the response to each perturbation, concentrations of ROS and healthy mitochondria transiently reach a new steady state. Upon every doubling of ROS synthesis, ROS concentration is first increased twice, but then is decreased back to a value a bit higher than the initial one. Upon every perturbation, the concentration of healthy mitochondria first decrease around 2 fold, but then increase back to the value a bit lower than the steady state value before perturbation.
We conclude that the system actively counteracts the increase of ROS. The homoeostasis in design 5 (model B5.3.cps) is stronger than in design 4 (model B3.cps)

Model building and simulations:

Models B5.1, B5.2, B5.3.1, B5.3.2, and all simulations for generation of every figure have been assembled in the model archive file (SM.B5).

The general idea used in the modelling is described below:

Models B5.1, B5.2 and B5.3 were built by adding the two forms of DJ1 protein (DJ1active and DJ1inactive) to model B4.

DJ1 was activated (oxidized by ROS) in irreversible mass action reaction 21: v=kf*ROS(t)*DJ1inact(t)

DJ1 was inactivated in irreversible mass action reaction 22: v=kf*DJ1act(t).

In model B5.1, DJ1active inhibited the inactivation of NFκB signaling (re 17): v=S*(Vm/(Km+DJ1act(t))), Vm and Km were fit in such a way as to maintain the same steady state concentration of NFκB as in model B4.

In model B5.2, DJ1active inhibited the inactivation of Nrf2 (re14): v= S*Vm*Keap1act/(Km+ DJ1act(t)), the rate constants were adjusted in such a way as to maintain the same steady state concentrations of Nrf2act and Nrf2inactive as in model B4.

In model B5.3.1 (the complete design 5) DJ1active inhibited both inactivation of NFκB (re17) and inactivation of Nrf2 (re14).

First, concentrations of DJ1 active and inactive were fixed (DJ1 was insensitive to ROS) and model was checked to behave identically to model B4.

In model B5.3.2 derived from model B5.3.1 the capacity of DJ1 activation was reduced 2 fold. In order to obtain the reduced DJ1 activity, the total concentration of DJ1 was reduced two fold, but the rate of the reaction of DJ1 inactivation was also reduced 2 fold (to obtain the same initial steady state).

Computations were performed in ds. Perturbations were performed using “time event” function in COPASI. The “factor time” was accumulating in time. At the moment “factor time” reached a certain level, the events were triggered.

Section C. Design C. Model C.

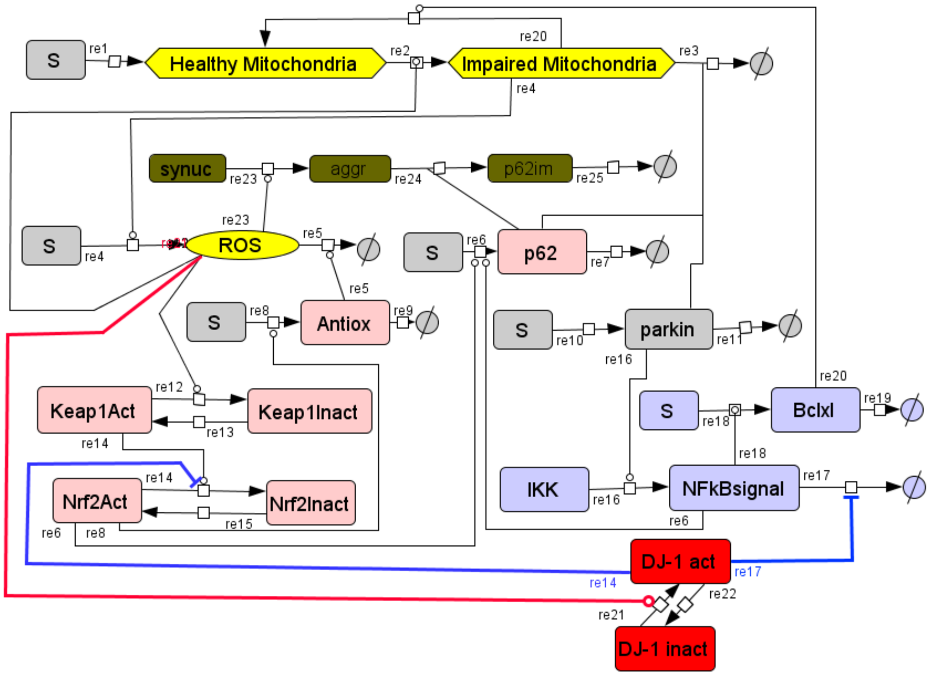

Supplementary Figure C.1. Design C (Model C). Network diagram of Model C including the α-synuclein module.

Model C was derived from Model B5 by incorporating a constant source of α-synuclein, ROS-dependent polymerisation of α-synuclein (reversible mass action reaction 23), sequestration of p62 by α-synuclein aggregates (reversible mass action reaction 24) and degradation of α-synuclein aggregates with p62 (irreversible mass action reaction 25).

A

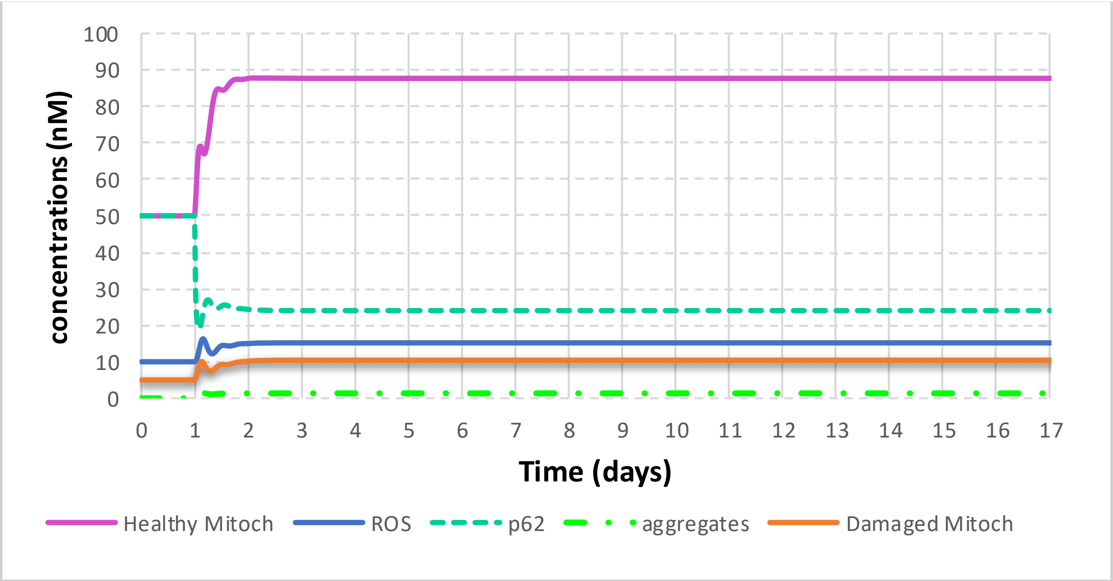

B

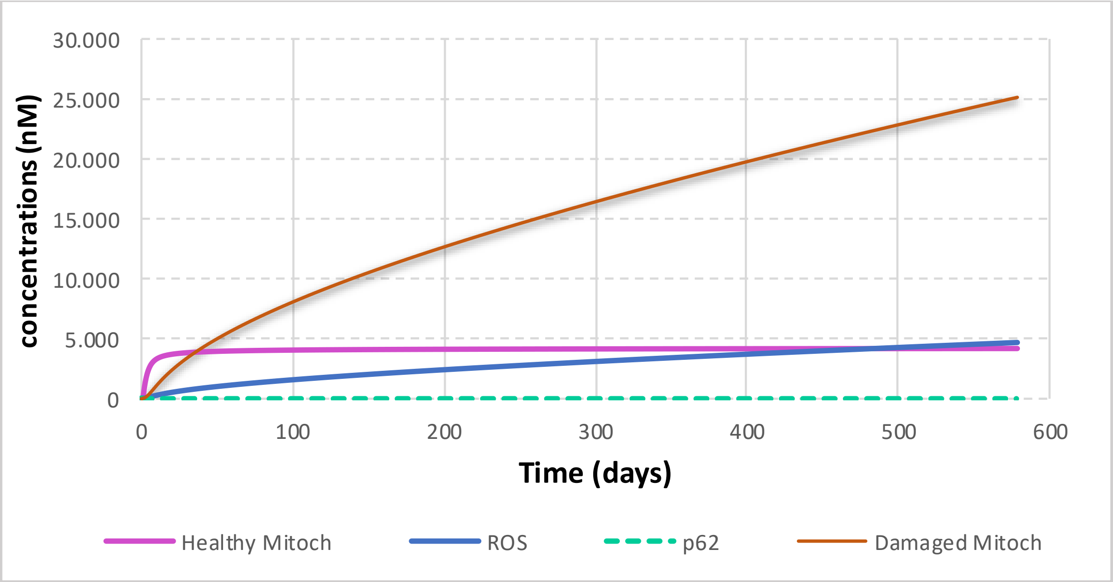

Supplementary Figure C.2. Design C (Model C). The double role of α-synuclein. Concentrations of ROS and p62 (in nM), of α-synuclein aggregates and of healthy and damaged mitochondria (in arbitrary units) for design C. First, α-synuclein was absent and system was at a steady state. On day 1, the concentration of α-synuclein was increased. Oxidation of α-synuclein by ROS caused the formation of α-synuclein aggregates. Then, α-synuclein aggregates sequestered p62. This process reduced mitophagy and helped to increase the concentration of healthy mitochondria. In A, the concentration of α-synuclein on day 1 was increased from 0 till 0.1 a.u. In B, the concentration of α-synuclein on day 1 was increased from 0 till 0.1 a.u.

The following conclusions were drawn from Supplementary Figure C.2:

A) The system reaches a new steady state with the concentration of healthy mitochondria approximately 80% higher than the initial one. The concentration of damaged mitochondria and ROS increases approximately 50%.
B) The concentration of damaged mitochondria constantly increases and the system explodes (as it is no longer able to reach a steady state).

We conclude that at low concentration α-synuclein helps to accumulate healthy mitochondria. However, high α-synuclein concentration results in accumulation of damaged mitochondria and ROS, and causes the catastrophe.

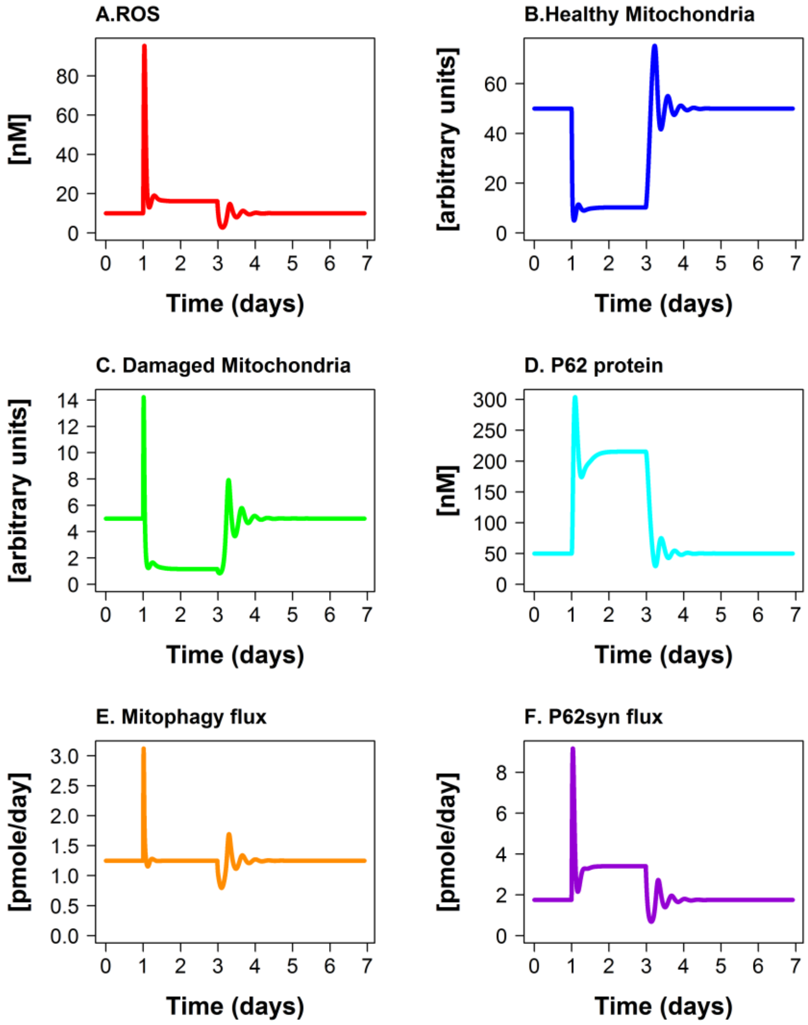

Supplementary Figure C.3. Design C (Model C). Response to increase of the ROS generation rate constant (10 fold) in model C without α-synuclein. Concentrations of ROS and p62 in nM, α-synuclein aggregates, healthy and damaged mitochondria in arbitrary units, fluxes in pmoles/day are shown for model C in response to the change in ROS generation rate constant. The ROS

generation rate constant was increased 10 fold on day 1 and decreased 10 fold back to the initial level on day 3.

The following conclusions were drawn from Supplementary Figure C.3:

A) When the ROS generation rate constant is increased on day 1, ROS concentration goes up. After a sharp peak, the ROS concentration decreases again and stays at a level around 60% higher than before perturbation. The observation that ROS concentration increases just 60% while ROS generation rate constant is increased 10 fold suggests a strong ROS homeostasis. When ROS generation is decreased 10 fold, ROS concentration returns to the initial value.
B) When the ROS generation rate constant is increased 10 fold on day 1, the concentration of healthy mitochondria drops 10 fold as well. However, after two damping oscillations, the concentration of healthy mitochondria increases again and stays at a level around 5 fold below the initial level. When ROS generation is decreased 10 fold, the concentration of healthy mitochondria returns to the initial value.
C) Following the perturbation on day 1, the concentration of damaged mitochondria decreases around 4 fold. After two damping oscillations, the concentration of damaged mitochondria increases and stays at value around 5 fold below the initial level. Upon the decrease of ROS generation, the concentration of damaged mitochondria returns to the initial value.
D) The concentration of p62 protein increases substantially (around 4 fold) after the increase of ROS generation on day 1 and returns to the initial level when ROS generation is decreased on day 3.
E) Following the increase of ROS generation, the mitophagy flux increases quickly. However, after short damping oscillations the mitophagy flux returns to the initial level.
F) Levels of p62 increase upon the increase of ROS generation. After short damping oscillations p62 synthesis reaches an elevated level. Upon the decrease of ROS generation, the flux of p62 synthesis returns to the initial steady state level.

We conclude that Model C without α-synuclein exhibits strong homeostasis against 10 fold increase of the ROS generation rate constant.

The following conclusions were drawn from Supplementary Figure C.3:

A) ROS concentration substantially increases and reaches a new steady state.
B) The concentration of healthy mitochondria substantially increases and reaches a new steady state.
C) The concentration of damaged mitochondria substantially increases and reaches a new steady state.
D) The concentration of p62 substantially decreases and reaches a new steady state.
E) The concentration of aggregates substantially increases and reaches a new steady state.
F) The concentration of sequestered p62 substantially increases and reaches a new steady state.
G) The Mitophagy flux quickly drops, but then, with the increased synthesis of p62, mitophagy returns to the initial level.
H) P62 synthesis flux substantially increases and reaches a new steady state.

We conclude that Model C, upon addition of a constant source of α-synuclein, reaches a new steady state with higher concentration of both healthy mitochondria and ROS.

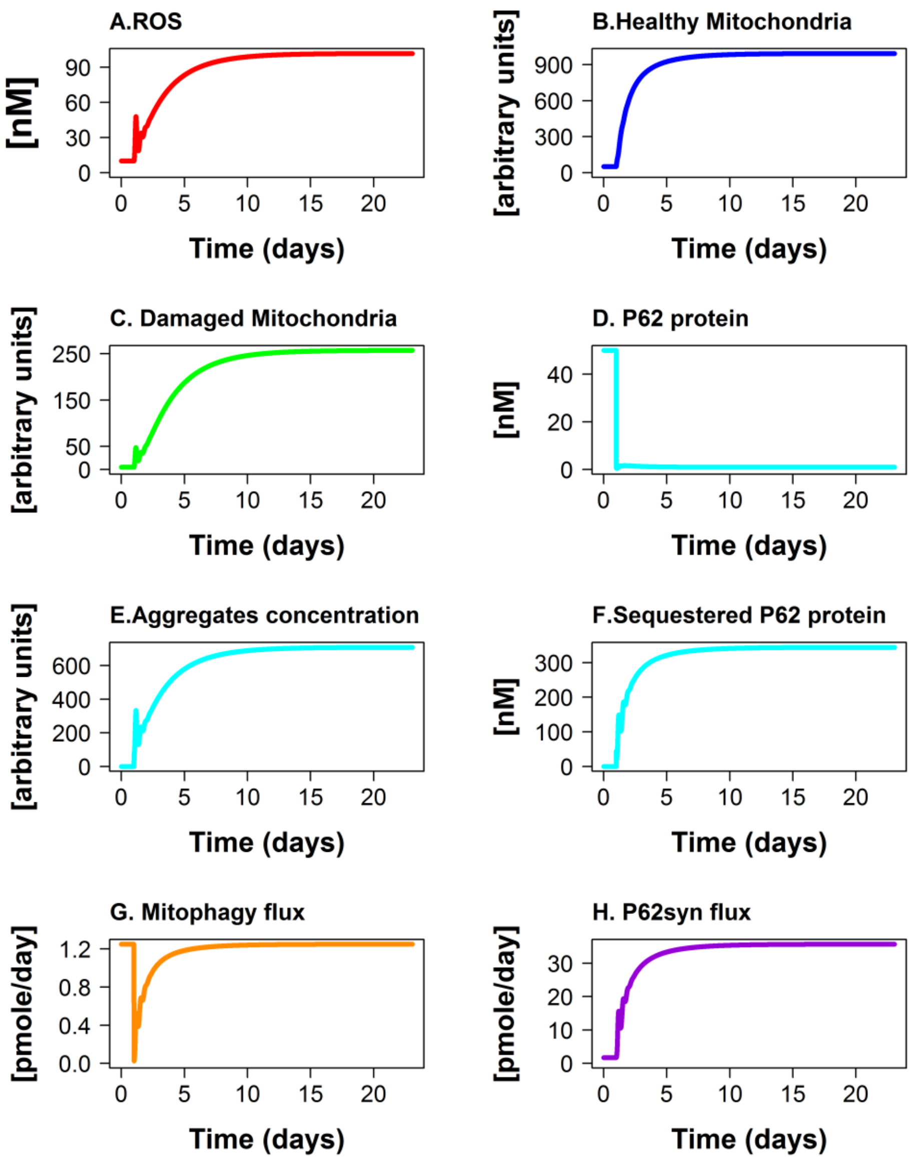

Supplementary Figure C.4. Design C (Model C). Response to addition of a constant source of α-synuclein. Concentrations of ROS (A), p62 (D) and sequestered p62 (F) in nM, healthy (B) and damaged (D) mitochondria and α-synuclein aggregates (E), in arbitrary units, and fluxes of mitophagy (G) and of p62 synthesis (H) in pmoles/day are shown for model C in response to addition of a constant source of α-synuclein on day 1.

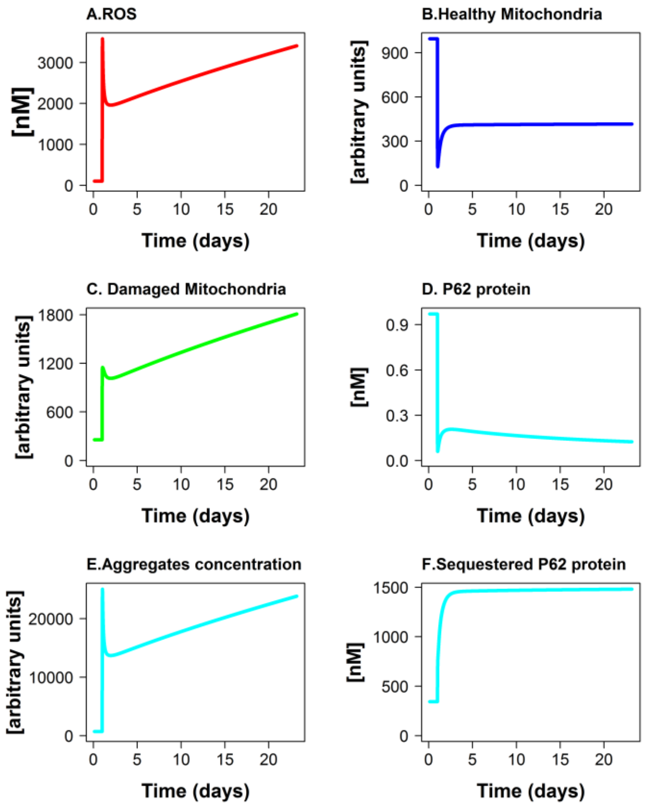

Supplementary Figure C.5. Design C (Model C). Response to the increase of ROS generation rate constant in the model with α-synuclein. Model C with the constant source of α-synuclein was in the steady state found in Supplementary Figure C.4. On day 1, the ROS generation rate constant was increased 10 fold (similarly to the perturbation described in Supplementary Figure C.3). The changes in the concentration of ROS (A), p62 (D) and sequestered p62 (F) in nM, healthy (B) and damaged (C) mitochondria and α-synuclein aggregates (E) in arbitrary units were plotted.

The following conclusions were drawn from Supplementary Figure C.5:

A) ROS concentration continuously increases and does not reach steady state. B) The concentration of healthy mitochondria first decreases around 8 fold, but then recovers 3 fold and reaches a new steady state on a value around 2 fold lower than before the increase of ROS generation. C) The concentration of damaged mitochondria continuously increases and does not reach steady state. D) p62 concentration drops 7 fold and reaches very low level (0.1 nM).
E) The concentration of aggregates increases and does not reach a steady state. F) The concentration of sequestered p62 increases more than 4 fold and reaches a new steady state.

We conclude that Model C with α-synuclein does not reach a steady state when the ROS generation rate constant is increased.

Section D. Comprehensive Model D: fitting to experimental data

		mRNA fold changes			
time, min	ROS	Antiox	P62	Bcl-xl	Nf-κB
0	0.53	1	1	1	1
60	13	1	1	1.1	1.08
120	40	1.1	1.1	0.95	1.05
240	75	1.2	1.6	0.83	1.12
360	90	1.21	1.2	1	1.05
480	90	1.25	2.2	0.85	1
1440	20	2.2	3.2	1.02	0.63

Supplementary Table D.T1. Response to menadione addition. Fold of change in the relative concentrations of ROS and mRNAs for p62, antioxidant response - NQO1 representative gene, Bclxl and NFκB after addition of menadione (100 μM) to HepG2 cells, starting from 1 at the steady state.

Model D fitting to experimental data

H_2O_2	time, min	0	60	120	180	240	300	360	1440	2880	4320	10080
	time, h	0	1	2	3	4	5	6	24	48	72	168
50 μM	Sample Continued treat. (% of CTR)	100	81	89	137	96	78	91	49	50	39	27
	Sample Pulse treat. (% of CTR)	100		128		119		119	50	45	43	21
	Sample Repeated treat. (% of CTR)	100		88	59	57	54	28				
150 μM	Sample Continued treat. (% of CTR)	100	32	62	60	74	61	83	17	37	21	9
	Sample Pulse treat. (% of CTR)	100		48		65		91	35	36	22	7
	Sample Repeated treat. (% of CTR)	100		19	20	12	10	3				
300 μM	Sample Continued treat. (% of CTR)	100	16	38	8	44	12	55	6	20	5	3
	Sample Pulse treat. (% of CTR)	100		28		52		65	32	31	16	3
	Sample Repeated treat. (% of CTR)	100		5	4	2	3	0				

Supplementary Table D.T2. Response to hydrogen peroxide addition: Changes in the relative concentration of ATP. ATP concentration (% of CTR) after either single or periodic

addition of H_2O_2 to PC12 cells. For single treatments, H_2O_2 (50 μM, 150 μM, or 300 μM) was added at 0h. Single treatments were either continued (peroxide was left in the medium and allowed to go through its spontaneous degradation), or pulse (peroxide was washed out after 30 min). For periodic treatments (to simulate chronic oxidative stress) H_2O_2 (50 μM, 150 μM, or 300 μM) was added to cells every 1h. Results of single continued treatments were very similar to the results of pulse treatments. Thus, we did not differentiate between pulse and continued treatments during model fitting and call them both "single treatment". Data are the mean ± SEM of three-four independent experiments in duplicate. $^*p \leq 0.05$, $^{**}p \leq 0.01$, vs. $^{***}p \leq 0.001$ versus their respective CTR (ANOVA and Dunnett's multiple comparisons test).

A

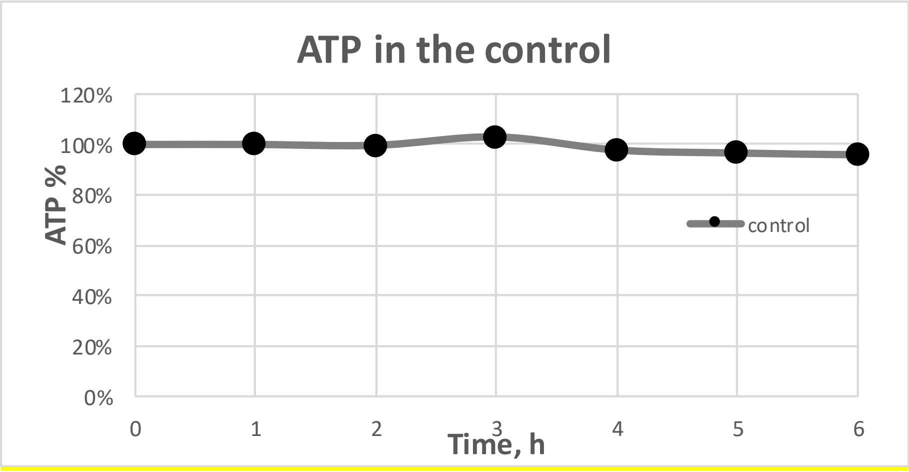

B

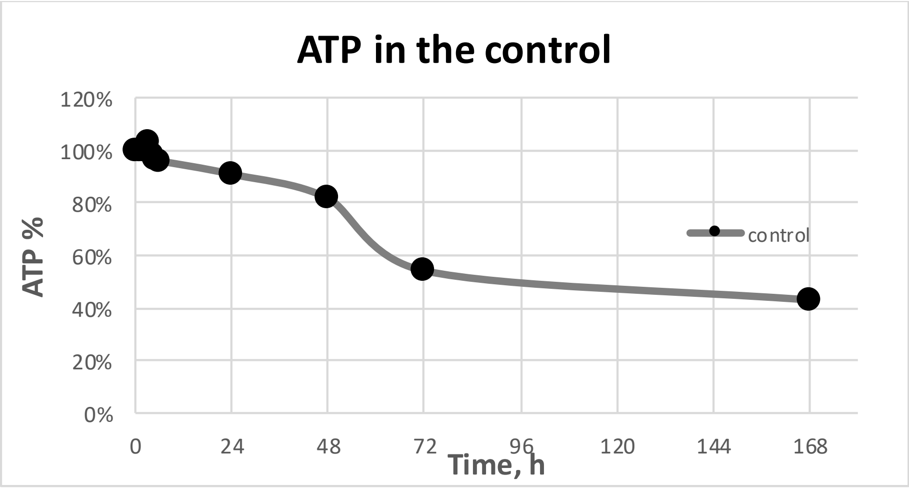

C

time, min	0	60	120	180	240	300	360	1440	2880	4320	10080
time, h	0	1	2	3	4	5	6	24	48	72	168
control ATP %	100	**100**	**100**	**103**	**98**	**97**	**96**	**91**	**82**	**54**	**43**

Supplementary Figure D.1. Changes in control samples in time. Changes in ATP concentration (in %) in the control plotted in the time scale of first 6 hours (A) and of 7 days (B). In C the data used are presented. Data are the mean ± SEM of four independent experiments in duplicate. $*p \leq 0.05$, $**p \leq 0.01$, vs. $***p \leq 0.001$ versus their respective CTR (ANOVA and Dunnett's multiple comparisons test).

The following conclusions were drawn from Supplementary Figure D.1:

A) ATP concentration in the control in the time scale of first 6 hours does not change.

B) ATP concentration in the control declines with a severe drop between day 2 and 3.

We conclude that the control is stable within the first 6 hours, thus we only used data of the first 6 hours.

Model D fitting to experimental data

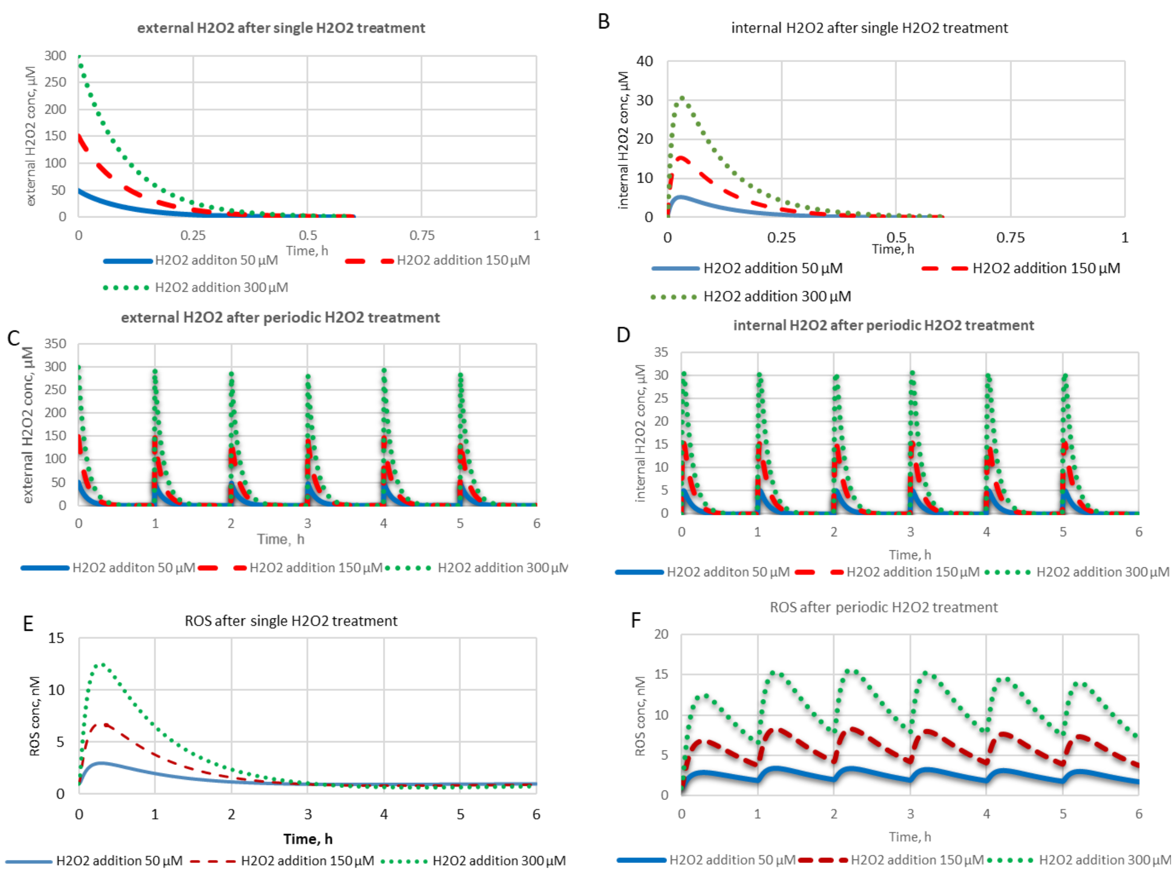

Supplementary Figure D.2. Time course of H_2O_2 (A-D) and ROS (E-F) in detailed model D. H_2O_2 (50 µM, 150 µM or 300 µM) was added either one time at the time 0h for each single treatment (A, B, E), or every 1h for periodic treatments (C, D, F). Intracellular (internal) and extracellular (external) concentrations of H_2O_2 (in µM) and ROS concentrations (in nM) were calculated as described in Material & Methods section. Data are the mean ± SEM of three-six independent samples. $*p \leq 0.05$, $**p \leq 0.01$, vs. $***p \leq 0.001$ versus their respective CTR (ANOVA and Dunnett's multiple comparisons test).

The following conclusions were drawn from Supplementary Figure D.2:

We conclude that the dynamics of ROS concentrations followed the dynamics of H_2O_2 addition with a certain delay.

Section E. Memory in comprehensive Model D

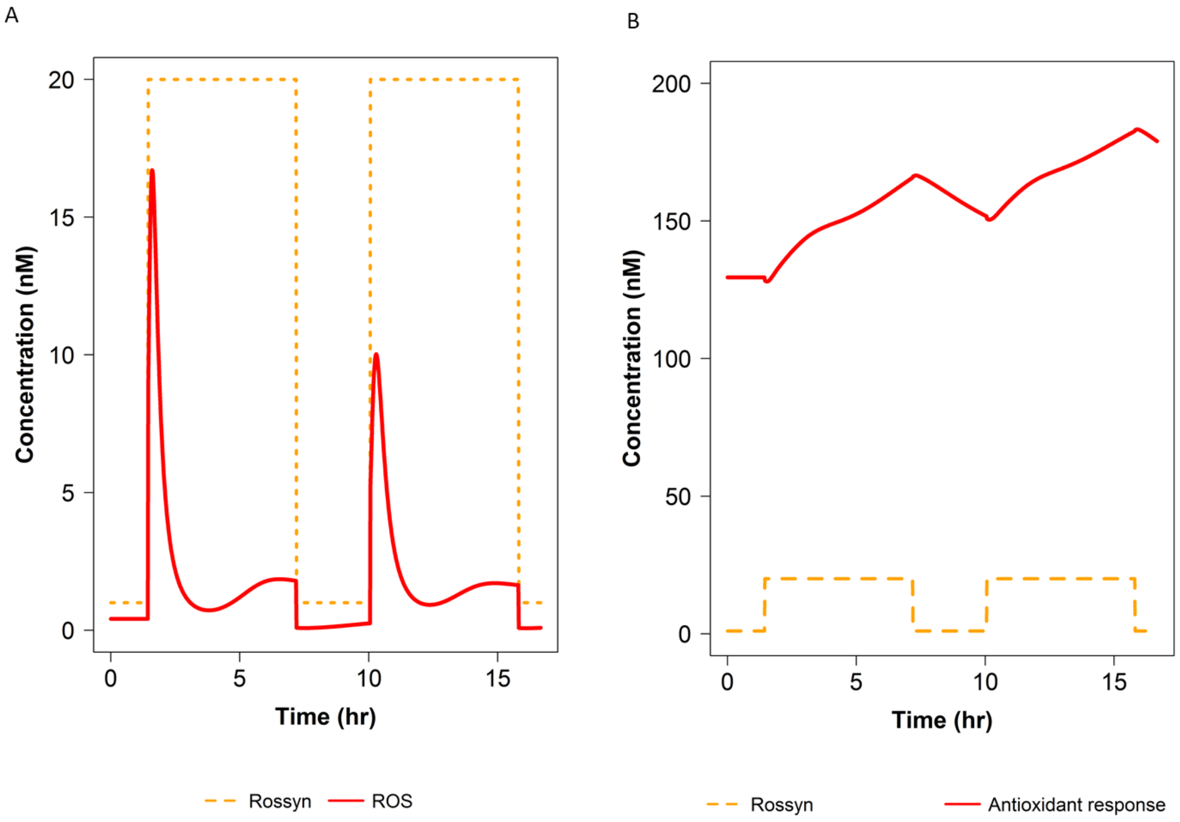

Supplementary Figure E.1. Preconditioning to oxidative stress in detailed model (Model D). The effect of the increased ROS generation rate constant on the concentration of ROS (A) and Antioxidant Response (B) was simulated in detailed model D. We started with the steady state. Then, two pulses of the increased ROS generation rate constant were applied: at 1.47h the ROS generation rate constant was increased 20 fold, at 7.2h decreased 20 fold, at 10h increased 20 fold again, and at 15.8h decreased 20 fold back (dashed orange line).

The following conclusions were drawn from Supplementary Figure E.1:

A) With every pulse of ROS generation, ROS concentration (red line) increases. However, the increase of ROS concentration was higher after the first pulse (37 fold, from 0.45 nM to 16.75 nM) than after the second pulse (22 fold, from 0.45 nM till 10 nM).

B) With every pulse of ROS generation, an antioxidant response (both antioxidant enzymes and antioxidants) changes (red line). After the first pulse of ROS generation, the antioxidant response was activated. At the time of the second pulse, the system has already an elevated level of antioxidant response.

We conclude that the preconditioning caused by the first stress stimuli may enable (train) the system to deal with the consequent stress.

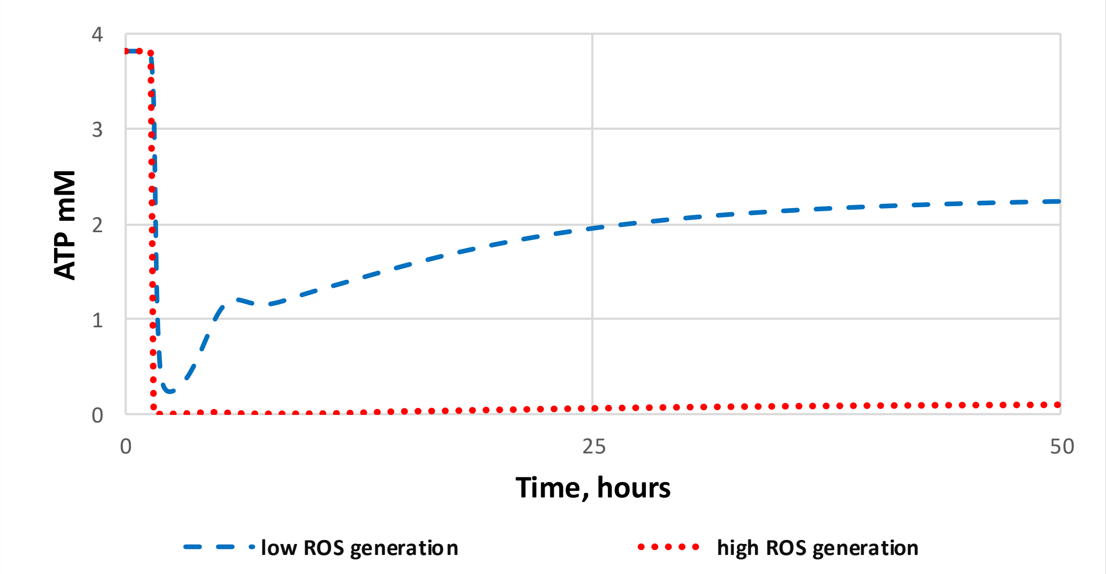

Supplementary Figure E.2. Bi-stability in the detailed model D. Changes in ATP concentration (in mM) was simulated. At the point of 20 year, the ROS generation rate constant was increased either to the smaller (e.g. 20 fold) (dashed blue line) or to the higher extend (e.g. 200 fold) (dotted red line).

The following conclusions were drawn from Supplementary Figure E.2:

At low increase of the ROS generation rate constant, the model exhibits homeostasis and ATP concentration quickly reaches a new steady state.

At higher increase of the ROS generation rate constant, ATP concentration quickly goes to 0.

We conclude that bi-stability is observed. The System is either stabilized or immediately collapsed. There is no gradual deterioration.

Section F. Re-examining 5 designs in the detailed model

All model and simulation results were assembled into the model archive file called 'SM.F'.

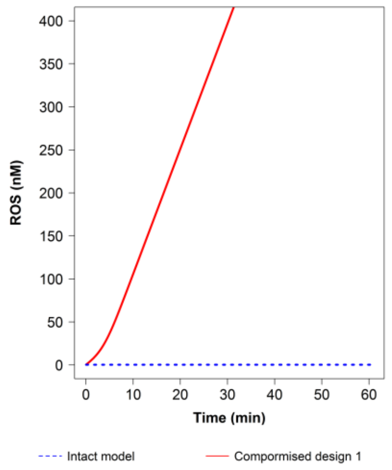

Supplementary Figure F.1. The mitophagy and the removal of ROS are required for achieving a steady state (design 1). The change in the concentration of ROS (in nM) was simulated in an intact detailed model D (dashed blue line) and in the model with compromised design 1, at which the reactions of ROS removal and mitophagy were deleted (solid red line)

The following conclusions were drawn from Supplementary Figure F.1:

The intact model maintains a steady state. In the model with compromised design 1, the concentration of ROS increases to infinity.

We conclude that mitophagy and the removal of ROS are required for achieving a steady state, thereby confirming that the design 1, identified first in a simplified model B1, works also in the detailed model D.

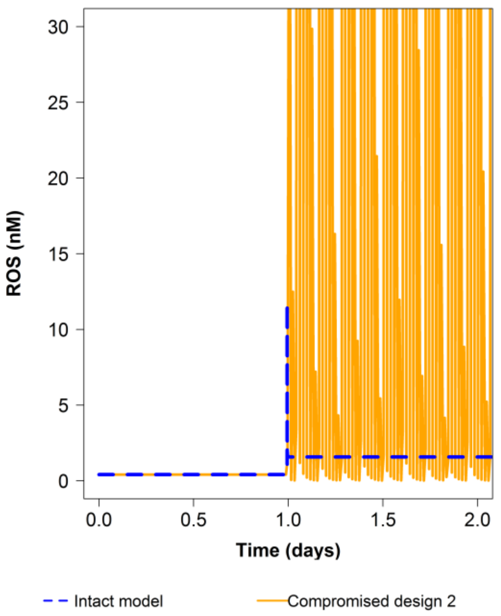

Supplementary Figure F.2. Structural robustness is achieved when the concentration of healthy mitochondria is variable (design 2). On day 1, the ROS generation rate constant was increased 50 fold. The change in the concentration of ROS (in nM) was simulated in an intact detailed model D (dashed blue line) and in the model with compromised design 2, at which the concentration of healthy mitochondria was constant (orange line).

The following conclusions were drawn from Supplementary Figure F.2:

In the intact model the concentration of ROS reaches a new steady state after a short transient peak. The model with compromised design 2 enters into an oscillatory mode.

We conclude that a variable concentration of healthy mitochondria is required to achieve structural robustness, thereby confirming that the design 2, identified first in a simplified model B2B, works also in the detailed model D.

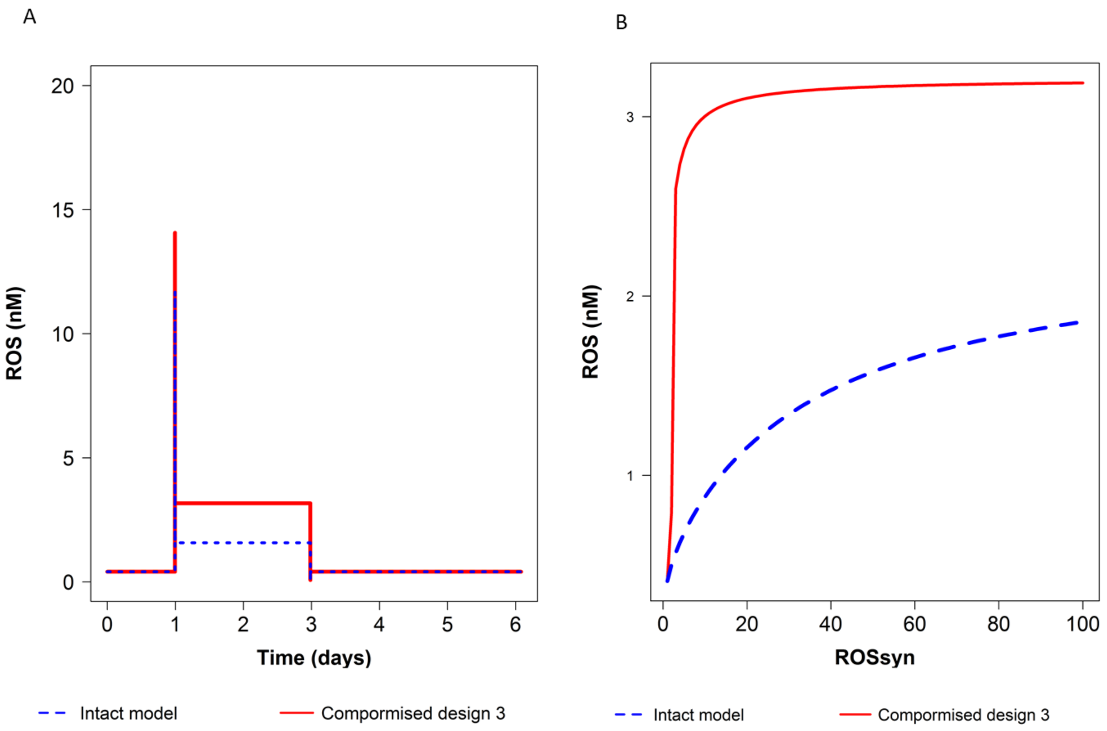

Supplementary Figure F.3. Keap1-Nrf2 signaling provides homeostasis (design 3). The change in the concentration of ROS (in nM) was simulated in an intact detailed model D (dashed blue line) and in the model with compromised design 3, at which the Keap1-Nrf2 regulation was turned off. In A, the ROS generation rate constant was increased 50 fold on day 1 and returned to the initial value on day 3. In B, the steady state concentration of ROS was plotted versus the ROS generation rate constant.

The following conclusions were drawn from Supplementary Figure F.3:

A) Changes in the ROS concentration in the intact model are less pronounced than in the model without Nrf2-Keap1 signaling.

B) For the same ROS generation rate constants, a steady state concentration of ROS is lower in the intact model comparing to the model where Nrf2-Keap1 signaling is turned off.

We conclude that Nrf2-Keap1 signaling contributes to the strength of ROS homeostasis, thereby confirming that the design 2, identified first in a simplified model B2B, works also in the detailed model D.

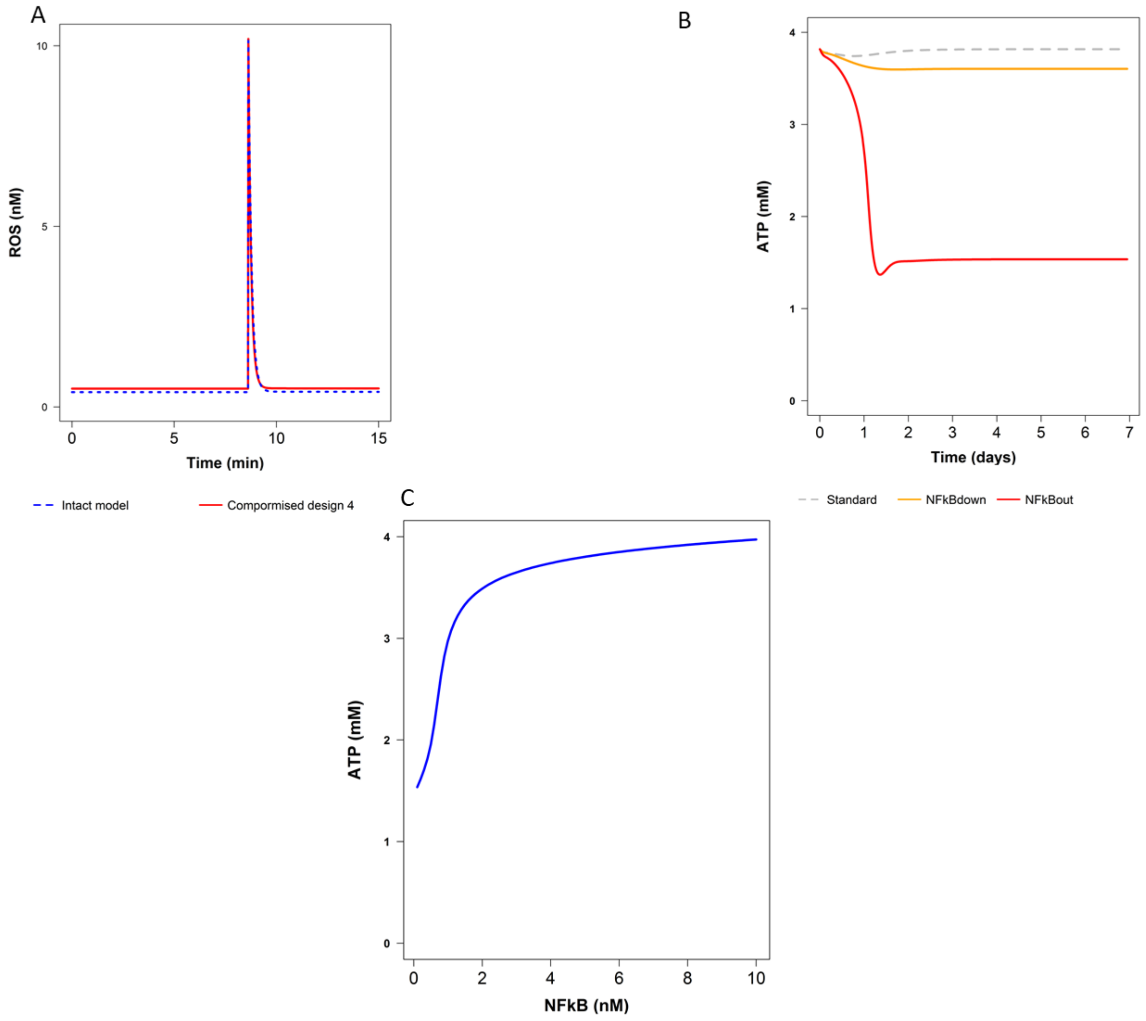

Supplementary Figure F.4. The robustness against the perturbations of the initial ROS concentration in the detailed model D (design 4). The changes in the concentration of ROS (in nM) and ATP (in mM) were simulated in the intact detailed model D and in the model with the reduced activity of NFkB signaling. In A, ROS (10 nM) was injected on day 8.6 in the model with (dotted blue line) and in the model without (red line) NFκB signaling. In B, NFκB signaling was knocked down (solid orange line) and knocked out (solid red line). In C, the steady state concentration of ATP was plotted versus the NFκB concentration (that is proportional to the activity of NFκB signaling. The model and the simulation results were assembled into the model archive file called ‘SM.F’.

The following conclusions were drawn from simulation of design Supplementary Figure F.4:

A) Both in the intact model, and in the model without NFκB signaling, the concentration of ROS increases temporary from 0.4 to 10 nM (25 fold) and quickly returns back to the initial steady state value.

B) The concentration of ATP decreases upon the decrease of NFκB signaling; less pronounced in the case of knockdown (solid orange line) and much more pronounced in the case of knockout (solid red line).

C) The higher is the NFκB signaling, the higher is the steady state concentration of ATP.

We conclude that in the detailed model we are unable to reproduce the design 4 in terms of the role of NFκB signaling in the robustness to the ejection of ROS. Nevertheless, NFκB signaling is important to achieve higher ATP concentration. The further role of NFκB signaling in detailed model is reported in Supplementary Information SM.G.

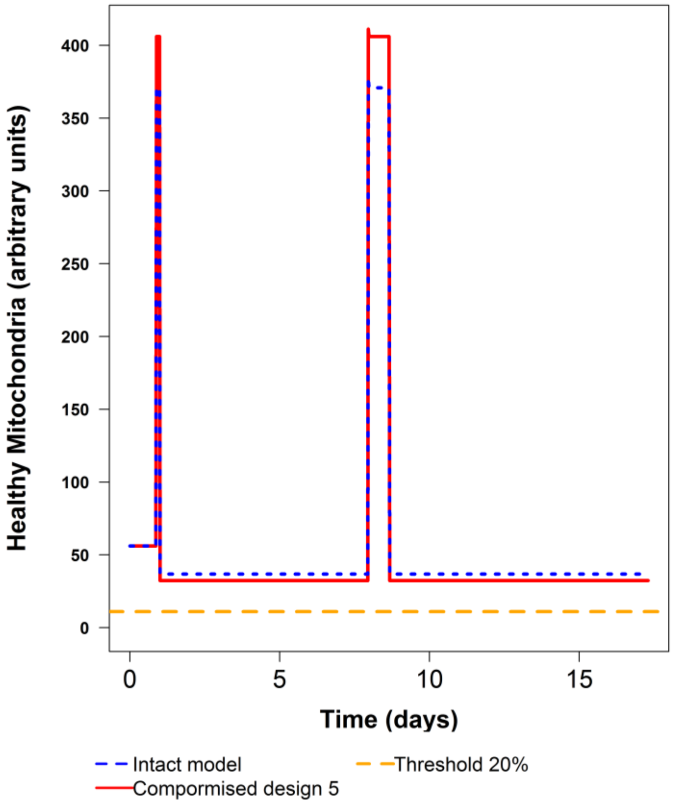

Supplementary Figure F.5. Stable homeostasis capable to deal with the second peak of ROS generation in detailed model D (design 5). The changes in the concentration of Healthy Mitochondria (in a.u.) were simulated in the intact detailed model D and in the model with the reduced activity of DJ-1 signaling. Initially, the model was in a steady state. At day 1, the ROS generation rate constant was increased 100 fold, but NFκB signaling was also activated 200 fold, just 3h before the increase of ROS generation. When models reached a new steady state, on day 8, the ROS generation rate constant was decreased 100 fold. When the model reached a new steady state again, on day 8.7, the ROS synthesis was increased 100 fold for the second time.

The following conclusions were drawn from simulation of design Supplementary Figure F.5:

Both the intact model and the model with reduced DJ-1 signaling are robust to the second peak of increased ROS generation. In both models the concentration of Healthy Mitochondria does not sweep below a viability line.

We conclude that in the detailed model, the design 5 is functioning in the sense that the system is robust to the second pulse of ROS. However, the role of DJ-1 in obtaining robustness is not clear, because other modules contributes to this robustness as well. The further role of DJ-1 signaling in detailed model is studied in Figure 5.

Section G. Aging with compromised NFkB signaling

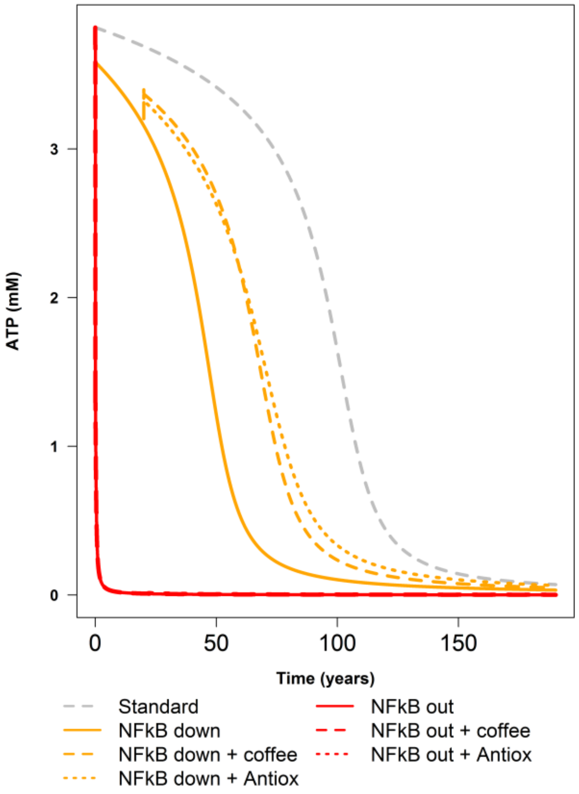

Supplementary Figure G.1. ROS induced aging in the detailed model D with compromised NFκB signaling. The change in the concentration of ATP (in mM) was simulated in the intact model (healthy aging), in the model where NFκB signaling was completely knocked out, and in the model where NFκB signaling was reduced 50% (knocked down). The effects of Nrf2-Keap1 activation (addition of coffee) or addition of antioxidants were simulated as well. The treatment by coffee started at 20 years; it was simulated by activation of Nrf2 nuclear import 1.5 fold. The

treatment by antioxidant started at 20 years as well and was simulated as the activation of antioxidants proteins synthesis 1.5 fold.

The following conclusions were drawn from Supplementary Figure G.1:

In the case of healthy aging (dotted grey line), ATP first gradually decreases during the first period of around 75 years. Then the decrease of ATP accelerates. ATP drops below 50% of initial level at around 100 year, and then quickly declines in a non-linear manner.

In the model without NFκB signaling, ATP (solid red line) drops immediately. Treatment with antioxidants (dotted red line) and with caffeine (dashed red line) does not help.

In the model with reduced NFκB signaling, ATP (solid orange line) decreases much earlier than in the standard situation. However, the treatment with antioxidants (orange dotted line) and with caffeine (orange dashed line) helps.

We conclude that the compromise of NFκB signaling accelerates ROS-induced aging. The help from the treatment with both coffee and antioxidants is very limited.

Section H. The effect of α-synuclein on ATP and ROS concentrations in the detailed model

All model and simulation results were assembled into the model archive file called 'SM.H'

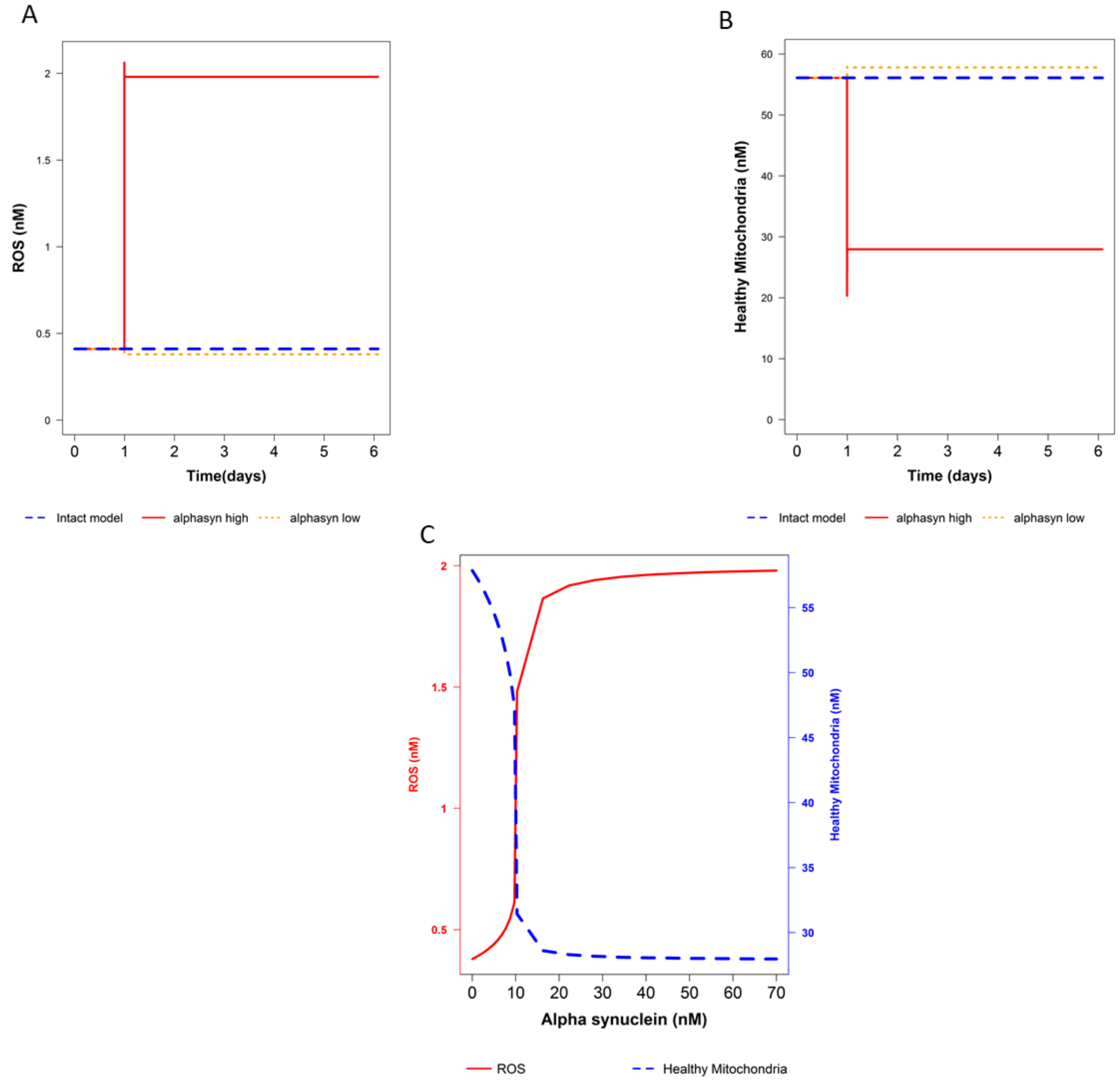

Supplementary Figure H.1. The effect of α-synuclein on ATP and ROS concentrations in detailed model. In A-B, the changes in the concentration of (A) ROS (in nM) and (B) Healthy Mitochondria (in a.u.) were simulated in the intact detailed model D (dashed blue line), in the model where the concentration of α-synuclein source was decreased on day 1 from 3 nM to 0.1 nM (dotted yellow line) and in the model where the concentration of α-synuclein source was increased on day 1 from 3 nM to 70 nM (red line). In C, the steady state concentration of ROS

(dashed blue line) and Healthy Mitochondria (red line) were plotted versus the concentration of α-synuclein source.

The following conclusions were drawn from Supplementary Figure H.1:

A) The increase of α-synuclein source results in the increase of ROS concentration.

B) The increase of α-synuclein source results in the decrease of the concentration of Healthy Mitochondria.

C) The higher is the concentration of α-synuclein source, the higher is the concentration of ROS and the lower (up to zero) is the concentration of healthy mitochondria.

We conclude that in the detailed model, α-synuclein causes the increase of ROS similarly to its action in the simplified model C (SM.C). However, in contrast to what was observed in the simplified model C, α-synuclein causes the decrease in the concentration of healthy mitochondria. The further role of α-synuclein is studied in Figure 5.

Section I. Aging-time-control coefficient

Definition

The aging-time-control coefficient is defined by:

$$C_i^{T_{0.5}} \stackrel{\text{def}}{=} \frac{dln(T_{0.5})}{dlnk_i} = \lim_{k_{i,1}\to k_{i,0}} \frac{T_{0.5,1}-T_{0.5,0}}{T_{0.5,0}} \cdot \frac{k_{i,0}}{k_{i,1}-k_{i,0}} = \lim_{k_{i,1}\to k_{i,0}} \frac{\tau_1-\tau_0}{\tau_0} \cdot \frac{k_{i,0}}{k_{i,1}-k_{i,0}} \cong$$

Second subscript ‘0’ refers to the standard state and second substrate ‘1’ to the perturbed state, the perturbation consisting of an change of rate constant k_i from its standard value $k_{i,0}$ to the perturbed value $k_{i,1}$. The perturbation causes the time at which ATP is half the initial unperturbed value to shift from $T_{0.5,0}$ to $T_{0.5,1}$. For brevity we define:

$$\tau_j \stackrel{\text{def}}{=} T_{0.5,j}$$

Here j is either 0 (i.e. no perturbation) or 1 (with perturbation). The control coefficient is virtually equal to the percentage increase in T_{50} (time where ATP is 50 % of its initial value;; this initial value differing between unperturbed and perturbed state, and therefore the $ATP_{50\%}$ differing between the two states) upon 1% activation of any molecular process i (here parameterized by k_i, the k_{-i} of the reverse process being altered in proportion to k_1).

Determination

In a numerical simulation one obtains a list of ATP values for a large number of time points, but rarely the ATP at any time point will precisely equal half the initial value. We may then take the time point just before the first time at which the ATP level is below 50%. For the perturbed system this will be a different time point however, and by not correcting for this shift, the numerical determination of the control coefficient will include an effect of the time dependence of ATP itself. We therefore use a method to estimate the time at which ATP is half its initial value (ATP= $ATP_{0.5,0}$) by linear interpolation between the time point just before ($T_{b,0}$) and the time point just after ($T_{a,0}$) ATP crosses the 50% level:

$$T_{0.5,0} \approx T_{b,0} + \frac{ATP_{b,0}-ATP_{0.5,0}}{ATP_{b,0}-ATP_{a,0}} \cdot \left(T_{a,0}-T_{b,0}\right)$$

The corresponding ATP levels are denoted by $ATP_{b,0}$ and $ATP_{a,0}$, respectively.

We do the same for the perturbed system:

$$T_{0.5,1} \approx T_{b,1} + \frac{ATP_{b,1}-ATP_{0.5,1}}{ATP_{b,1}-ATP_{a,1}} \cdot \left(T_{a,1}-T_{b,1}\right)$$

For the control coefficient this leads to:

$$1 + \frac{k_{i,1}-k_{i,0}}{k_{i,0}} \cdot C_i^{T_{0.5}} = \lim_{k_{i,1}\to k_{i,0}} \frac{T_{0.5,1}}{T_{0.5,0}} \cong (Eq. I.0)$$

It is unlikely that the control coefficients with respect to the time at which ATP has dropped to x % depend strongly on the value of x. Rather than to determine the control of this time, we therefore switch to the control of the time at which ATP has dropped to $ATP_{b,0}$, which is equal to $T_{b,0}$. For the perturbed system:

$$T_{b,0} \approx T_{b,1} + \frac{ATP_{b,1} - ATP_{b,0}}{ATP_{b,1} - ATP_{a,1}} \cdot \left(T_{a,1} - T_{b,1}\right)$$

Hence:

$$1 + \frac{k_{i,1} - k_{i,0}}{k_{i,0}} \cdot C_i^{T_{bo,0}} = \lim_{k_{i,1} \to k_{i,0}} \frac{T_{bo,1}}{T_{b0,0}} \cong \frac{T_{b,1} + \frac{ATP_{b,1} - ATP_{b,0}}{ATP_{b,1} - ATP_{a,1}} \cdot \left(T_{a,1} - T_{b,1}\right)}{T_{b,0} + \frac{ATP_{b,0} - ATP_{b,0}}{ATP_{b,0} - ATP_{a,0}} \cdot \left(T_{a,0} - T_{b,0}\right)}$$

$$= \frac{T_{b,1} + \frac{ATP_{b,1} - ATP_{b,0}}{ATP_{b,1} - ATP_{a,1}} \cdot \left(T_{a,1} - T_{b,1}\right)}{T_{b,0}}$$

And therefore:

$$C_i^{T_{0.5}} \cong C_i^{T_{bo,0}} \cong \left(T_{b,1} + \frac{ATP_{b,1} - ATP_{b,0}}{ATP_{b,1} - ATP_{a,1}} \cdot \left(T_{a,1} - T_{b,1}\right) - T_{b,0}\right) \cdot \frac{k_{i,0} \cdot T_{b,0}}{k_{i,1} - k_{i,0}} \quad (Eq\ I.1)$$

The points ‘b,1’ and ‘a,1’ in the time series of the perturbed system should be chosen as the points immediately before and after the time at which the perturbed system crosses the ATP level equal to the ATP ($ATP_{b,0}$) level in the unperturbed system immediately before the time ($T_{b,0}$) where the unperturbed system crosses the 50% level.

We also computed the control coefficients in this second manner by using Eq. I.0 and we found the results to differ only in the fourth digit after the decimal separator.

Time-control by simultaneous $\Delta k_i/k_i$ activation of all molecular processes was computed by the equation I.1, similarly as for individual time control coefficients, with the only difference that all processes were activated simultaneously.

The sum of all individual time-control coefficients upon $\Delta k_i/k_i$ activation of each molecular processes should be equal to -1 and should also equal the time-control by simultaneous $\Delta k_i/k_i$ activation of all molecular processes (Westerhoff, 2008). This was confirmed numerically.

Section J. Control of aging

Re № COPASI	Molecular process	Life-time control coefficient
7	p62 synthesis	1.7
19	p62 transcription	1.7
3	KEAP1 damage	1.5
35	Degradation of damaged mitochondria	1.5
39	Synthesis of apoptotic machinery	1.5
12	Synthesis of antioxidant proteins	1.5
10	Transcription of antioxidant genes	1.5
40	ROS removal	1.5
15	NRF2 synthesis	1.4
17	NRF2 nuclear import	1.4
33	Degradation of damaged KEAP1	1.4
34	Damaging of mitochondria by ROS	1.1
29	Cellular ATP consumption	1.1
14	Synthesis of mitochondria	-2.6
27	KEAP1 synthesis	-1.8
20	p62RNA-ase	-1.7
38	Degradation of apoptotic machinery	-1.5
11	Degradation of antioxidant mRNA	-1.5
41	ROS synthesis	-1.5
13	Degradation of antioxidant protein	-1.5
17b	Nuclear export of NRF2	-1.4
3b	Repair of KEAP1	-1.4
2	Binding of KEAP1 to NRF2	-1.2
8	Oxidative phosphorylation	-1.1
61	Generation of damaging factors	-1.0
16	Binding of cytoplasmic p62 to ubiquitinated VDAC1	0.0
18	Binding of nuclear NRF2 to Response Element activating p62 transcription	0.0
42	Conversion of IKK to NFκB	0.8
1	Ubiquitination of VDAC1	0.8
9	Binding of nuclear NRF2 to Response Element activating antioxidant response genes	0.0
46	Activation of DJ1 by ROS	0.6
26	Binding of PINK1 to PARK2	0.00
45	Bclxl degradation	0.03
22	Transcription of ETC uncoupling proteins	0.01
24	translation of ETC uncoupling proteins	0.01
21	Activation of gene coding uncoupling proteins	0.00

31	Degradation of cytoplasmic KEAP1	0.00
32	Degradation of cytoplasmic p62	-0.9
43	Degradation of NFκB	-0.9
1b	Deubiquitinating of VDAC1	-0.8
47	Deactivation of DJ1	-0.6
36	Aggregation of α-synuclein	-0.4
4	NRF2 ubiquitination by KEAP1	-0.2
6	Binding of KEAP1 to cytoplasmic p62	-0.05
28	Degradation of the KEAP1_p62 complex	-0.07
62	Recovery of mitochondria	-0.03
44	Synthesis of Bclxl	-0.03
23	Degradation of mRNA of ETC uncoupling proteins	-0.01
25	Degradation of ETC uncoupling proteins	-0.01
21b	Deactivation of the gene encoding uncoupling proteins	-0.01
37	Binding of p62 to α-synuclein aggregates	0.00
50	Degradation of p62 immobilised on α-synuclein aggregates	0.00
30	Degradation of cytoplasmic NRF2	0.00
5	Degradation of ubiquitinated cytoplasmic NRF2	0.00
48	Removal of Cytochrome c	0.00
49	Release of Cytochrome c from mitochondria	0.00
59	Cellular death	0.00
**Sum of all individual time-control coefficients**		**-1.03**
**Time-control by simultaneous activation of all molecular processes**		**-1.00**

Supplementary Table J. Control coefficients for the control of the time of aging (aging-time-control coefficients) by all the 57 molecular processes in the comprehensive model of aging. The aging-time-control coefficients were calculated by increasing the activity of each of the processes mentioned in the first column by $\frac{\triangle k1}{k1} = 0.1\%$ and computing the % change in time at which ATP was 50% of maximal (equation I.1). Positive and negative values mean that aging is delayed or accelerated by the corresponding molecular process, respectively, by activation of the process. The sum of all individual aging-time-control coefficients was equal to -1.0 within the numerical resolution of the computer program, and is also equal to the aging-time-control by all processes together which is obtained by simultaneous activation of all molecular processes.

Section K. Calibration of the comprehensive model.

After the comprehensive model was shown to house the robustness principles, we retuned its parameters so that it would fit particular concentrations.

Changes without effect other than factorial changes in concentrations

We first consider three cases where changes were made that did not change anything in the model except for the amounts of some substances by a certain constant factor.

ATP/O stoichiometry, e/ATP stoichiometry

Starting from the comprehensive model D (), we rename 'O2' in the model to O2_1/5th'. In the old model the P/O ratio was taken to equal 0.5 (the ATP per O2 ratio equal to 1). Now we replace the O2, which was 250000, by O2_1/5th, which should then amount to 50000. We rename "RE" in the model to RE_4/5th. Hence the RE i.e. 5000000 (2.5 mM NADH; 5 mM 'electrons') would become RE_4/5th = 4000000 (initial value). To ensure that model steady state values do not change we divided the rate constant of ATP synthesis (which was 8.10-14) by 0.16. The rate constant then became 5.10-13. The same for ROSsynCorrected i.e. the rate constant parameter was divided by 0.16.

Cytochrome c concentration

In the old model this was 132 nM, (in reality 6.6 uM), which is 6600/132=50 times too small. Its initial concentration is increased by a factor of 50 from 132.2437661 to 6612 nM. Cytochrome c is involved in three following reactions: re59 (cell death; Ncells→deg), re49 Cyt C released from damaged mitochondria, and Re48 (Cytochrome c degradation; Cyt C → deg). The rate constants of the former two reactions were decreased and increased, respectively, by a factor of 50. That of Cytochrome c degradation (Re48) was left the same.

Mitochondrial concentration

The concentration of mitochondria was 82.8 nM, but should be 2.1 nM. The initial condition concentrations of Mit_Damage and Mit_Healthy were both decreased by a factor of 40. Not to disturb the steady state for all the parameters except these two, we activated the reactions catalyzed by these two and also reduced the synthesis rate of only the Healthy Mitochondria in order to decrease their steady state concentrations, all by the factor 40: The rate constants of re8, re49, re41 were increased by a factor of 40.

We checked that with the above changes the time dependence of the model remained the same: we computed the time dependence of the two models, and divided these by each other and found ratios of 1 for all concentrations, except mitochondria (40 fold decrease) and cytochrome c (50 fold increase).

Changes without effect on steady state concentrations but with effects on the dynamics

We next considered cases where changes were made that did change the time dependencies of substances without affecting the steady state concentrations.

Mitochondrial turnover

After the above modifications, the rate of synthesis of mitochondria was still 0.76 nM/min. This led to a lifetime at steady state of 82.8/0.76 = 109 minutes = 1.8 h. The mitochondrial lifetime was indeed shown by the model D as 70.8 minutes for the Healthy Mitochondria and 33.8 minutes for the damaged mitochondria, the sum of which is 104.6 minutes. When calibrating the model, we decreased the rate constants of re14 (ATP dependent synthesis of healthy mitochondria), re34 (mitochondria being turned into impaired mitochondria by ROS), re35 (degradation of impaired mitochondria) and mitochondria recovery reactions all by the same factors (of 5, 8, and 250) in order to increase the half-life of mitochondria from approximately 2 h up to 2 weeks. We checked that this operation did not affect the steady state concentrations but

did slow down the mitochondrial response in Supplementary Figure K2. In Supplementary Figure K1 we see that the oscillations are maintained particularly for higher ROS supply. Supplementary Figure K2 shows that design continues to provided robustness against two subsequent ROS pulse.

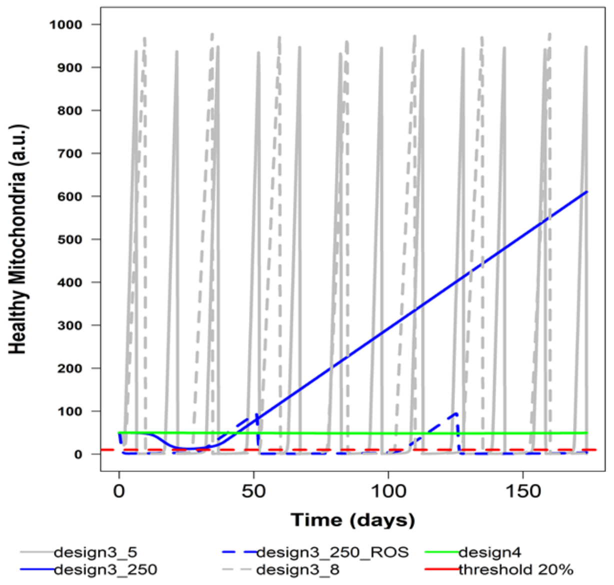

Supplementary Figure K1. Dynamic robustness in Design 4 (model B4) at various rates of mitochondrial turnover. The concentration of Healthy Mitochondria (in a. u.) is shown for Designs 3 (dashed and solid grey and blue line) and Design 4 (solid green line). The same models as for Fig. 2E were used, except that the turnover rates of the mitochondria were reduced (i.e. synthesis and degradation rate constants of the mitochondria were both reduced by factors of 5, 8, or 250) and the stoichiometries of the consumption of p62 and parkin per degraded mitochondrion were increased (by the same factor minus 1) so as to keep their consumption rates constant. Then the initial ROS concentration was perturbed (transient increase from 10 nM to 11 nM) at day 1. The dashed red line shows a hypothetical viability that corresponds to the threshold line dissecting 20% (10 a.u.) of the initial concentration of healthy mitochondria (50 a.u.). With both the design3_5 (solid grey line) and design3_8 (dashed grey line), which correspond to 5 and 8 fold decreased mitochondrial turnover an oscillation is induced. For the slowest mitochondrial turnaround times (approx. 2 weeks half-life) where turnover was reduced by 250 fold in design3_250 (blue solid line) there is no longer an oscillation, but rather a continuous increase in the level of healthy mitochondria. This is because the systems are running out of ROS. Increasing the rate of ROS synthesis by a factor of 10 revamped the oscillation at a reduced frequency.

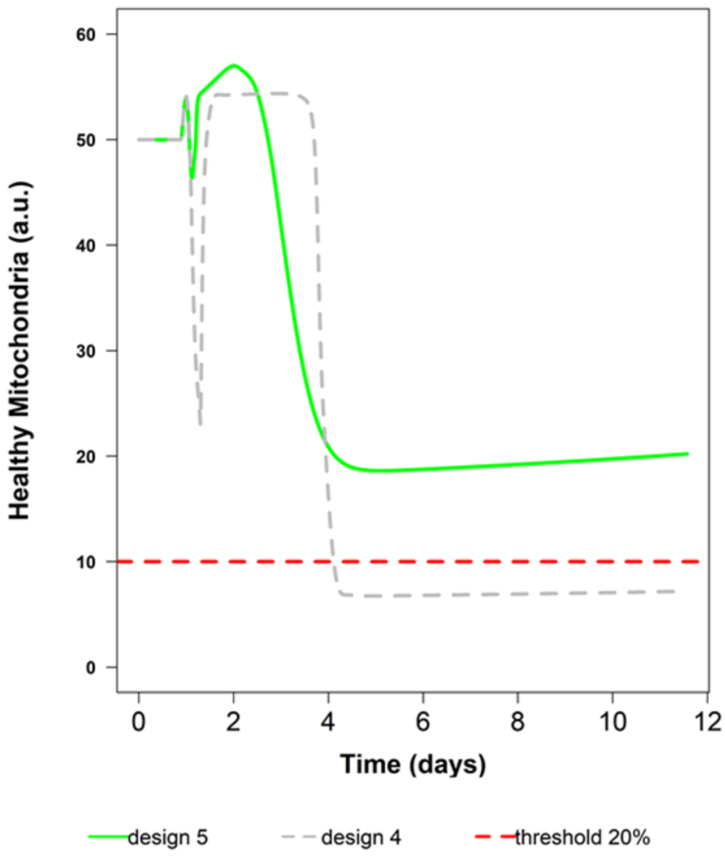

Supplementary Figure K2. Emergence of dynamic robustness vis-à-vis a second pulse of ROS in Design 5 (model B5) at a reduced mitochondrial turnover time. Computations as for Fig. 2F (main text) except that the mitochondrial; turnover was slowed down by a factor of 250 as in Fig. 2E' and the pulse strategy was somewhat different. The concentration of Healthy Mitochondria (in a. u.) is shown for Designs 4 (dashed grey line) and 5 (solid green line). The ROS generation rate constant was increased 15-fold on day 1, but, 3h before the increase of ROS generation, the NFκB signalling was increased 15-fold and the system reached a new steady state. After 5 hrs the ROS generation rate constant was decreased back to 15-fold, which allows the growth of healthy mitochondria. On the second day (at approx. 39 hr), the ROS generation rate constant was increased 15-fold for the second time.

Changes with effect on steady state concentrations and the dynamics

We next consider cases where changes were made that did change the time dependencies of substances and affect the steady state.

ATP/mitochondrial synthesis stoichiometry

In the old model only one ATP was used per unit mitochondria, which is about a million times slower than reality. We now assume that for the synthesis of one gram dryweight 57 mmol ATP is needed (approx. 5.7 ATP per amino acid; this number comes from the experimental Y_{ATP} reported by Stouthamer and Bettenhausen (1977) for *E. coli*), so for the synthesis of one mitochondrion 57 *0.022 *10-15= 1.3 fmol ATP=$1.3*10^{-15}*6.02*10^{23}$=7.7 10^8 molecules of ATP. In order to increase the stoichiometry of ATP consumption at the synthesis of 1 mitochondrion by this factor, we created an additional reaction, which we named *ATP dependent maintenance in* ATP consumption in mitochondrial synthesis (the traditional 'maintenance reaction' had been called 'cellular ATP consumption', re29). This new reaction had the same kinetics as the reaction of mitochondrial synthesis except that the mitochondrion was no longer mentioned as product. Its rate constant was taken to be 7.7 10^8 times the rate constant of the existing mitochondrial synthesis reaction (which consumed only 1 ATP per mitochondrion) and named it as k_ATP_recalibrated. Then we recalibrated the re29 (the maintenance reaction with rate constant 4 nmol/min) by changing the rate constant parameter for this reaction to 4 nmol/min minus k_ATP recalibrated.

Section L. Parameter values

The parameter values of the calibrated comprehensive model (D’) are given here:

Parameters	Type	Value	Units
**Compartment**			
cyt	fixed	1	l
nucleus	fixed	1	l
**Initial Species Values**			
KEAP1	Reactions	22.6161	nmol/(l)
KEAP1(ROS-modified)	Reactions	8.44849	nmol/(l)
VDAC1	Reactions	121.965	nmol/(l)
PARK2	Reactions	49.2276	nmol/(l)
S	Fixed	1	nmol/(l)
H2O	Fixed	5.55E+10	nmol/(l)
ATP	Fixed	3.82E+06	nmol/(l)
O2_1/5th	Fixed	50000	nmol/(l)
ADP	Fixed	2.73E+06	nmol/(l)
RE_4/5th	Fixed	4.00E+06	nmol/(l)
AntioxidantsProt	Reactions	129.524	nmol/(l)
NRF2cyt	Reactions	68.6233	nmol/(l)
PARK7Act	Reactions	54.6727	nmol/(l)
PARK7Inact(DJ-1)	Reactions	129.889	nmol/(l)
UncouplProtProt	Reactions	43.5368	nmol/(l)
PINKï¼‘	Reactions	40.8819	nmol/(l)
Mit-Healthy	Reactions	1.40255	nmol/(l)
Mit-Damaged	Reactions	0.668193	nmol/(l)
AP	Reactions	0.285607	nmol/(l)
aggr	Reactions	0.0102062	nmol/(l)
p62im	Reactions	1.22255	nmol/(l)
Alfa-synuclein	Fixed	3	nmol/(l)
p62cyt	Reactions	239.568	nmol/(l)
ROS	Reactions	0.410918	nmol/(l)
KEAP1:p62	Reactions	54.1809	nmol/(l)
PINK1:PARK2	Reactions	12.7871	nmol/(l)
Mtotal	Reactions	2.07074	nmol/(l)
VDAC1ub	Reactions	15.6081	nmol/(l)
VDAC:p62	Reactions	37.392	nmol/(l)
KEAP1:NRF2cyt	Reactions	0.200018	nmol/(l)
NRF2cytUB	Reactions	0.0999863	nmol/(l)
IKK	Fixed	1	nmol/(l)
NFkBsignal	Reactions	5.27336	nmol/(l)
Bclxl	Reactions	10.5467	nmol/(l)
CytC	Reactions	6612.19	nmol/(l)
H2O2	Fixed	0	nmol/(l)
H2O2_internal	Reactions	0	nmol/(l)
AO_mRNA_relative	Assignment	1	nmol/(l)
p62_mRNA_relative	Assignment	1	nmol/(l)

BCLXL_relative	Assignment	1	nmol/(l)
NFkB_relative	Assignment	1	nmol/(l)
ATP_relative	Assignment	1	nmol/(l)
ROS_relative	Assignment	1	nmol/(l)
deg	Fixed	0	nmol/(l)
Menadione	Reactions	0	nmol/(l)
Menadione_internal	Reactions	0	nmol/(l)
species	Fixed	1	nmol/(l)
Ncells	Reactions	100	nmol/(l)
ATP_measured	Assignment	1	nmol/(l)
Damage	Reactions	4.72E-19	nmol/(l)
AntioxidantsInactGene	Reactions	0.000991325	nmol/(l)
AntioxActGene	Reactions	0.000712675	nmol/(l)
AntioxidantsRNA	Reactions	0.0647886	nmol/(l)
p62mRNA	Reactions	0.0257356	nmol/(l)
p62actGene	Reactions	0.000514712	nmol/(l)
p62inactGene	Reactions	0.00118529	nmol/(l)
UncouplProtActGene	Reactions	0.000362806	nmol/(l)
UncouplProtRNA	Reactions	0.0226754	nmol/(l)
UncouplProtInactGene	Reactions	0.00132719	nmol/(l)
Snuc	Fixed	1	nmol/(l)
NRF2nuc	Reactions	68.6233	nmol/(l)
Aging	Reactions	0	nmol/(l)
**Initial Global Quantities**			
p62synt	Fixed	1	
KEAP1synt	Fixed	1	
Kf-Apoptosis	Assignment	0.0285607	
ROSsynt	Assignment	8.00E-14	
oscillations	Assignment	0.1	
Nrf2synt	Fixed	100	
Menadione_transport	Fixed	0.0007	
H2O2_transport	Fixed	0.18935	
celldeathCoefficient	Fixed	0	
CytCthreshold	Fixed	8250	
ROSsynCorrected	Assignment	8.00E-14	
ROSsyntCoeff	Assignment	1	
Factor_Time	Fixed	0	
coffee_addition_(fold_activation_of_Nrf2_synt)	Fixed	1	
Nrf2-Import	Fixed	1	
re8_Kf_modified	Assignment	5.00E-13	
re41_rosyN_modified	Assignment	5.00E-13	
re49_kf_modified	Assignment	25	
re59_k1_modified	Assignment	5.00E-08	
re8_kf_Mit_modified	Assignment	2.00E-11	
re14_k1_Mit_modified	Assignment	5.00E-09	
re49_kf_Mit_modified	Assignment	1000	
re41_rosyN_Mit_modfied	Assignment	2.00E-11	
Mit_turnovertime	Fixed	250	
re14_k1_Mittrurnover_modified	Assignment	2.00E-11	

re34_kf_Mittrurnover_modified	Assignment	4.00E-05	
Mitoch_recovery_kf_Mittrurnover_modified	Assignment	4.00E-07	
re35_kf__Mittrurnover_modified	Assignment	0.0004	
re14_ATP_recalibrated	Assignment	0.0154	
re29_ATP_recalibrated	Assignment	3.9846	
H2O2addition	Fixed	0	
Aging_rate_constant_re63	Assignment	1.50E-06	
aging_reduction_during_memoryPulse	Assignment	1	
ROSPulse	Assignment	1	
Aging_memory_pulse	Assignment	1	
Antiox_synthesis_rate_re12	Fixed	100000	
Antioxidant_treatment	Fixed	1	
Mitohormesis_factor	Fixed	1	
KEAP_deletion	Fixed	1	
KEAP_reverse	Fixed	0	
**Kinetic Parameters**			
re1_Ubiquitination_VDAC1		nmol/(l*min)	
kf		1	1/min
kb		100	1/min
re2change_KEAP1:NRF2cyt_Complex_formation		nmol/(l*min)	
Kinh		0.1	l/nmol
kf		0.5	l/(nmol*min)
kb		100	1/min
re3_KEAP1_Oxidation		nmol/(l*min)	
kf		1	l/(nmol*min)
kb		1	1/min
re4_KEAP1:NRF2cyt_dissociation		nmol/(l*min)	
k1		500	1/min
k2		0.01	l/(nmol*min)
re5_NRF2cytUB_Degradation		nmol/(l*min)	
k1		1000	1/min
re6_KEAP1:p62_Complex_formation		nmol/(l*min)	
k1		60	l/(nmol*min)
k2		6000	1/min
re7_p62cyt_Synthesis		nmol/(l*min)	
kf		20	l/(nmol*min)
re8_ATP_Production		nmol/(l*min)	
kf		2.00E-11	lÂ³/(nmolÂ³*min)
kUncouplProt		0.0001	l/nmol
re9_Activation_of_AntioxidantActGene		nmol/(l*min)	
k1		0.022	l/(nmol*min)
k2		2.1	1/min
re10_Transcription_antioxidants_genes		nmol/(l*min)	
kf		0.1	l/(nmol*min)
re11_AntioxidantRNA_degrdation		nmol/(min)	
k1		0.0011	l/min
re12_Antioxidant_Protein_Synthesis		nmol/(l*min)	
kf		100000	l/(nmol*min)
re13_Antioxidant_Protein_Degrdation		nmol/(l*min)	

k1		50	1/min
re14_ATP_dependent_synthesis_healthy_mitochondria		nmol/(l*min)	
k1		2.00E-11	l/(nmol*min)
re15_NRF2cyt_Synthesis		nmol/(l*min)	
k1		100	1/min
re16_VDAC:p62_complex_formation		nmol/(l*min)	
k1		6	l/(nmol*min)
k2		600	1/min
re17_reversible_NRF2_transport_reaction		nmol/(min)	
k1		1	l/min
k2		1	l/min
re18_p62actgene_activation		nmol/(l*min)	
kf		0.03	lÂ²/(nmolÂ²*min)
kb		25	1/min
re19_p62mRNA_Synthesis		nmol/(l*min)	
kf		0.1	l/(nmol*min)
re20_p62mRNA_Degradation		nmol/(min)	
k1		0.002	l/min
re46and47_PARK7Act_Synthesis		nmol/(l*min)	
kf		1	1/min
kb		1	1/min
re21_Activation_gene_coding_uncoupling_proteins		nmol/(l*min)	
kf		1	l/(nmol*min)
kb		200	1/min
re22_Transcription_ETC_uncoupling_proteins		nmol/(l*min)	
kf		50	l/(nmol*min)
re23_mRNA_ETC_uncoupling_proteins_Degradation		nmol/(min)	
k1		0.8	l/min
re24_ETC_uncoupling_proteins_translation		nmol/(l*min)	
kf		192	l/(nmol*min)
re25_Degradation_of_ETC_uncoupling_proteins		nmol/(l*min)	
k1		0.1	1/min
re26_PINK1:PARK2_Complex_formation		nmol/(l*min)	
kf		1	lÂ²/(nmolÂ²*min)
kb		10	1/min
Kinh		0.1	l/nmol
re27_KEAP1_synthesis		nmol/(l*min)	
k1		1	1/min
re28_KEAP1:p62_degradation		nmol/(l*min)	
kf		0.002	l/(nmol*min)
re29_Cellular_ATP_consumption		nmol/(l*min)	
k1		3.9846	1/min
re30_NRF2cyt_Degradation		nmol/(l*min)	
k1		0.0002	1/min
re31_KEAP1_Degradation		nmol/(l*min)	
k1		0.0001	1/min
re32_p62cyt_Degradation		nmol/(l*min)	
k1		0.001	1/min
re33_oxidizedKEAP1_Degradation		nmol/(l*min)	

k1		0.1	1/min
re34_Mitochondria_being_turnned_into_impaired_mitochondria_by_ROS		nmol/(l*min)	
kf		4.00E-05	l/(nmol*min)
re35_Degradation_Impaired_Mitochondria		nmol/(l*min)	
kf		0.0004	l/(nmol*min)
re36_agg_synthesis		nmol/(l*min)	
kf		0.1	l/(nmol*min)
kb		0.1	1/min
re37_p62im(aggr:p62cyt)_complex_formation		nmol/(l*min)	
k1		0.1	l/(nmol*min)
k2		0.1	1/min
re38_Apoptotic_machinery_Degradation		nmol/(l*min)	
k1		0.1	1/min
re39_Apoptotic_machinery_Synthesis		nmol/(l*min)	
k1		0.0285607	1/min
re40_Scavenging_ROS_ByAntioxidantsProt		nmol/(l*min)	
k1		0.05	l/(nmol*min)
re41_ROS_synthesis		nmol/(l*min)	
kf		2.00E-11	lÂ²/(nmolÂ²*min)
kb		0	1/min
KUncouplProt		0.0001	l/nmol
re42_Conversion_IKK_to_NFkB		nmol/(l*min)	
k1		0.005	1/min
re43_Degradation_of_NFkB_(-signal)		nmol/(l*min)	
kf		0.001	1/min
Kinh		0.001	l/nmol
re44BClXl_synthesis		nmol/(l*min)	
kf		1	l/(nmol*min)
re45_BclXl_degradation		nmol/(l*min)	
k1		0.5	1/min
re49_CytochromeC_Release		nmol/(l*min)	
kf		1000	l/(nmol*min)
Kinh		0.001	l/nmol
re48_CytochromeC_Degradation		nmol/(l*min)	
k1		0.1	1/min
re50_P62im(P62:Aggr)_Degradation		nmol/(l*min)	
k1		0.1	1/min
Mitoch_recovery		nmol/(l*min)	
kf		4.00E-07	l/(nmol*min)
H2O2_degradation_internal		nmol/(l*min)	
k1		1.18425	1/min
H2O2_transport		nmol/(l*min)	
k1		0.18935	1/min
k2		0.18935	1/min
Menadione_transport		nmol/(l*min)	
k1		0.0007	1/min
k2		0.0007	1/min
Menadione_internal_degradation		nmol/(l*min)	
k1		0.003	1/min

Menadione_induces_ROS		nmol/(l*min)	
kf		0.033	l/(nmol*min)
Menadione_external_dergadation		nmol/(l*min)	
k1		0.001	1/min
H2O2_degradation_external		nmol/(l*min)	
k1		0	1/min
re59_Cell_Death		nmol/(l*min)	
k1		5.00E-08	?
kCellDeath		0	?
Damage_accumulation		nmol/(l*min)	
kf		0.0538893	l/(nmol*min)
re14'_ATP_dependent_maintaice		nmol/(l*min)	
k1		0.0154	l/(nmol*min)
Damage_induces_ROS		nmol/(l*min)	
kf		0.0027819	l/(nmol*min)
Damage_removal		nmol/(l*min)	
k1		0.0159841	1/min
re63_Aging_reaction		nmol/(l*min)	
k		1.50E-06	lÂ³/(nmolÂ³*min)
power		4	1

Section M. ROS and aging

As shown in Fig. 5A of the main text, ATP decreases when aging occurs. In the same computation ROS increases around the same time, as shown here:

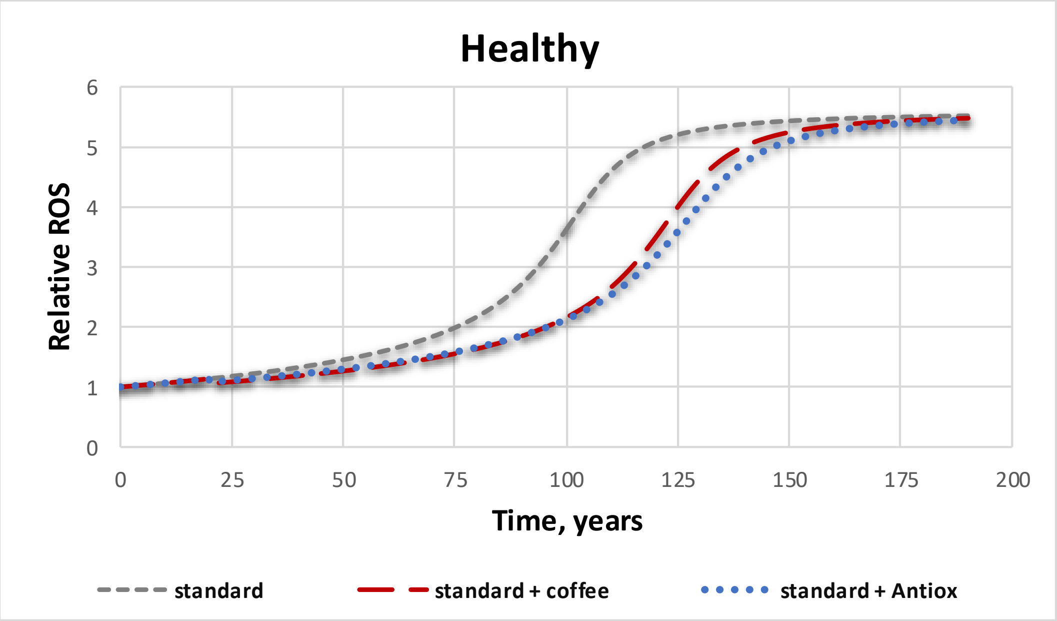

Supplementary Figure M. Simulated ROS(t) in a healthy cell without any treatment (dashed grey line), and when treated with antioxidants, starting at 20 years as well, and simulated as the activation of antioxidant proteins synthesis (1.2-fold, dotted blue line), or with caffeine, started at 20 years and simulated by activation of the Nrf2 nuclear import (1.2-fold, dashed red line).

Section N. Triple extra validation: mitohormesis, preconditioning and dual role of NRF2

As this paper was under review a reviewer asked whether the model could reproduce three important phenomena. We translated this into three concrete questions:

(i) *Can the model illustrate the concept of mitohormesis i.e. that moderately increased levels of ROS could extend life time?* We increased the activity of the ROS signal reception by the mitoptosis proteins by 50% (we increased the rate constant of reaction 39 by 50%) and found the lifetime to increase by 80% in the aging model. We here show the resulting figure:

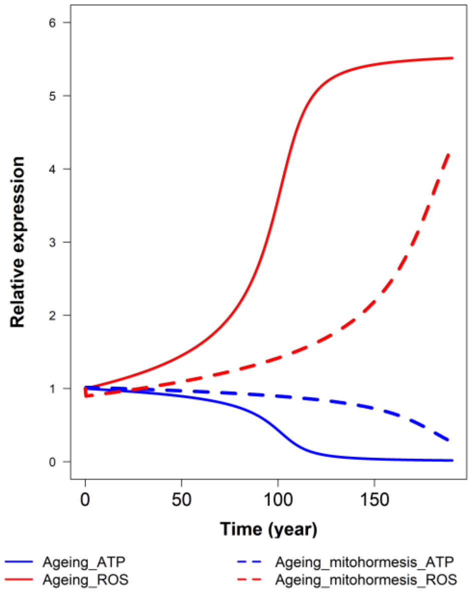

Supplementry Figure N1. Mitohormesis in aging model. The relative fold expression of ROS (blue) and ATP (red) in normal Ageing model (solid line) and increased mitohormesis activated model (dashed line). We increased the activity of the ROS signal reception by the mitoptosis proteins by 50% (we increased the rate constant for reaction 39 by 50%) and found the lifetime to increase by 80% in the aging model. All computations in calibrated comprehensive model.

(ii). Can the model show that ROS paradoxically protects against subsequent ROS insults? We have given two consecutive pulses of ROS to the model and measured the ATP, Healthy Mitochondria and ROS (aging model). With the second pulse, there was a smaller increase in ROS. There are fewer healthy mitochondria that can be pulsed into becoming impaired mitochondria with elevated ROS production. And also the ATP, which was already lower, was brought to a lower level because of a loss of healthy mitochondria. Here is the figure showing this:

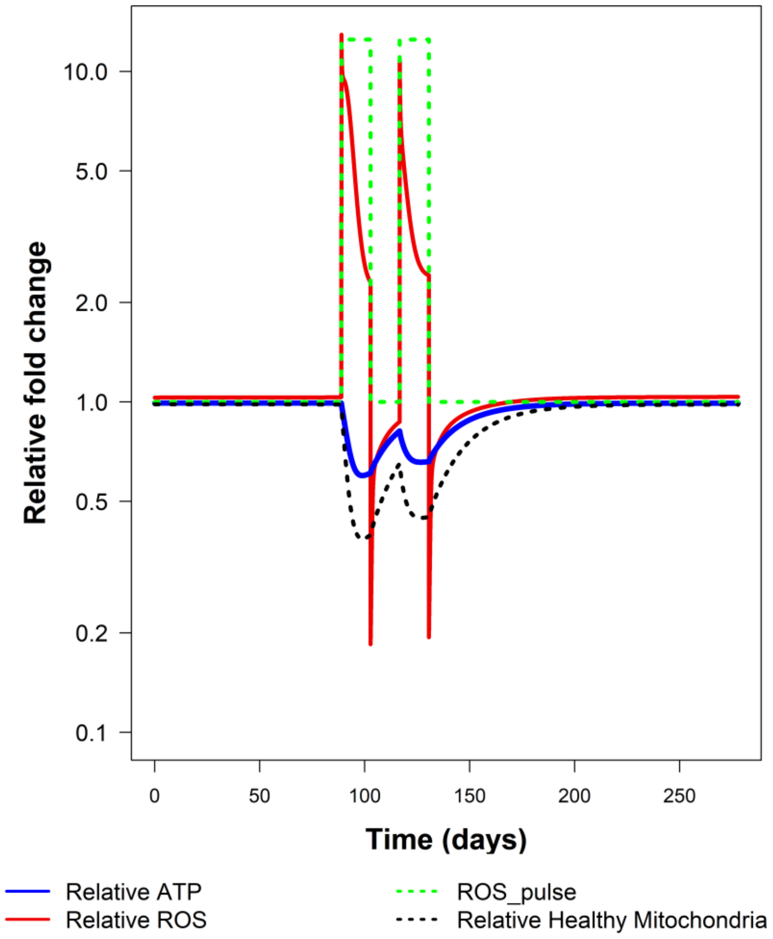

Supplementary Figure N2. Memory in the aging model. The relative change in ATP (solid blue line), ROS (solid red line) and Healthy mitochondria (black dashed line) with the addition of two consecutive pulse of ROS (green dashed line). With the second pulse, there is a smaller increase in ROS, because there are fewer healthy mitochondria that can be pulsed into becoming impaired mitochondria with elevated ROS production. And also the ATP, which was already lower, decreased less because of a loss of healthy mitochondria. All computations in calibrated comprehensive model.

iii. Can the model illustrate the dual role of NRF2 in the context of Keap-1? Keap-1 deficiency should lead to more active NRF2. The role of Nrf2 in our model is to stimulate the removal of damaged mitochondria through mitoptosis, thereby to reduce autocatalytic ROS production, to make cells resistant to apoptosis and thereby delay aging. This should be expected to make tumor cells more persistent, in agreement with the observation cited by the reviewer. As apoptosis is essential in early embryogenesis, NRF2 activation by Keap-1 loss of function should thereby be expected to be lethal embryonically and to stimulate tumor cell persistence. In order to confirm this we knocked-down the KEAP1 in the comprehensive

model. We observed a seven thousand increase in NRF2 fold change (enormous activation). Also, there was a decrease in damaged mitochondria and cytochrome c levels, which in our model also stands for decreased apoptosis. We here reproduce the figure:

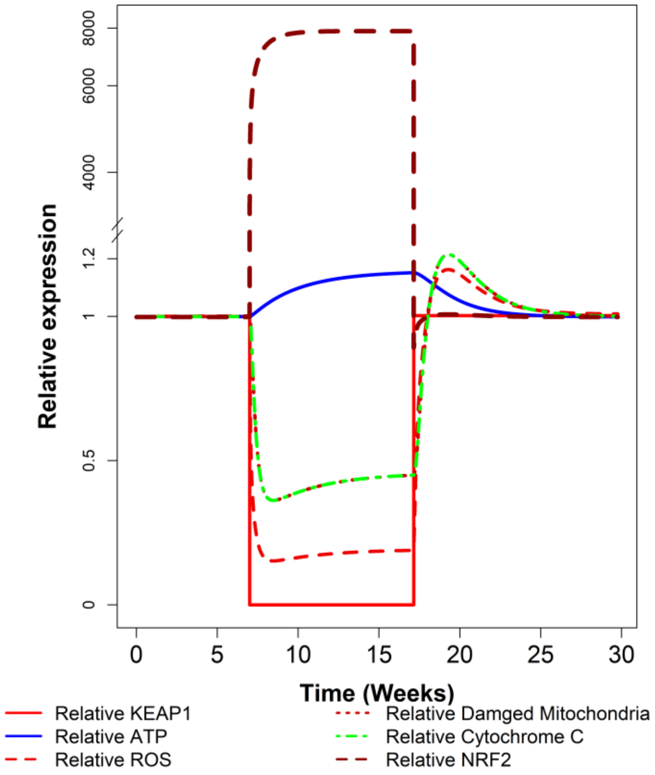

Supplementary Figure N3. KEAP1 deletion effect: We created an event in the aging model by knocking-down the KEAP1 (complete deletion of KEAP1 for a certain period of time). Red solid line shows there is no KEAP1 from 8th weeks to 17th weeks approximately. During this period there a strongly increased NRF2 level is applied (dashed dark red color). Also, there is a decrease in Damage mitochondria (dashed light red), ATP (solid blue line) and cytochrome c (dashed green line) levels. Computations in calibrated comprehensive model.

Section O. Sensitivity analysis

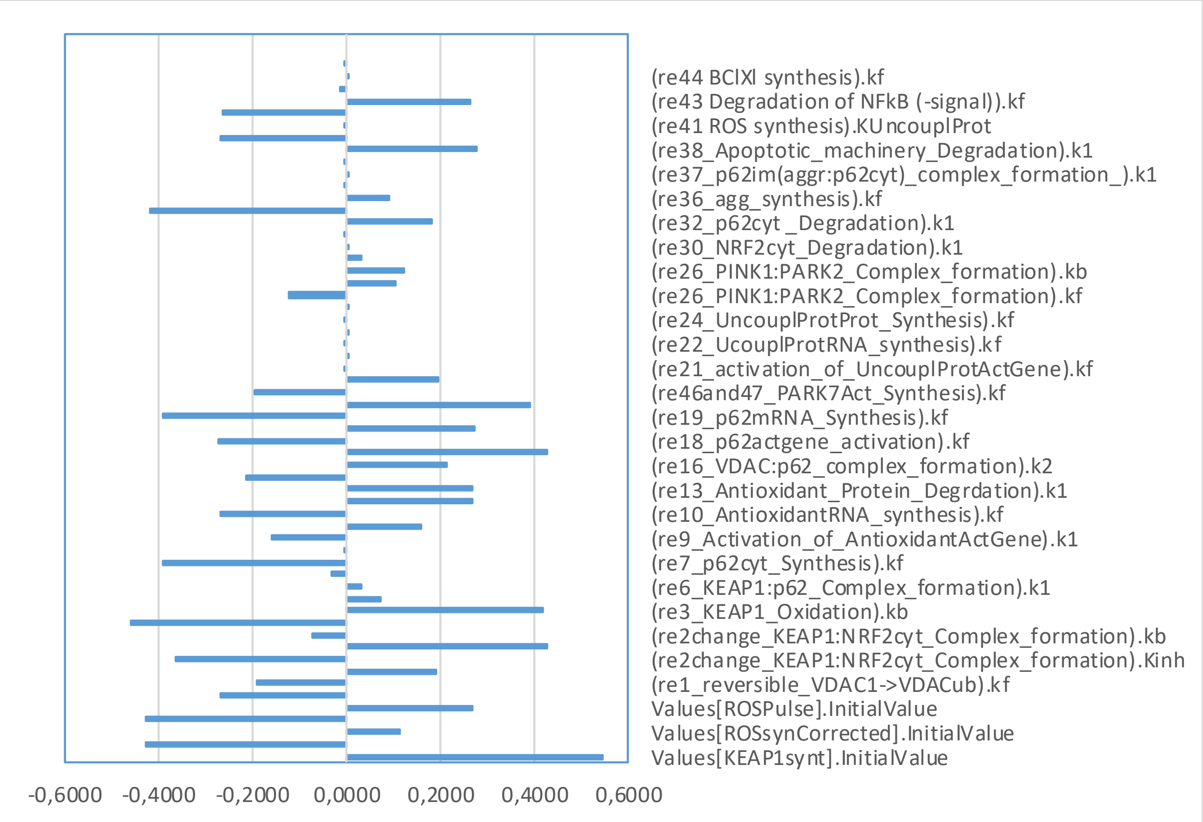

Supplementary Figure O. Sensitivity analysis of the dependence of steady state ROS levels on various parameters. Computed for the calibrated comprehensive model in the absence of aging.

Section P. On the roles of Parkin, p62 and mitochondria in the various designs, as dependent versus independent variables

In our COPASI versions of models, Parkin and p62 are present and in all designs, but in some they are dependent variables and in others they are considered fixed, i.e. independent variables (=parameters). In design 1 and in design 2 the concentrations of Parkin and p62 are fixed (at 50 nM). Accordingly, p62 and Parkin are shown as grey boxes in the corresponding network diagrams. In these designs the rate constant for mitophagy is fixed (but not the rate of mitophagy itself, which depends on the concentration of substrate). In design 3 we added the ROS-induced Nrf2-Keap1 loop that activates mitophagy via the regulation of the p62 level, which herewith becomes a dependent variable. Starting from design 3 (i.e. in designs 3, 4, and 5), the concentration of both p62 and Parking is variable (i.e. 'floating') (as shown by the pink boxes in the corresponding diagrams): consequently, the higher is the rate of mitophagy due to a higher concentration of p62, the higher is the rate of Parkin degradation in mitophagy, because the degradation of Parkin is coupled to the degradation of p62. However, because the *increased* consumption of Parkin in mitophagy is very small as compared to the degradation of Parkin in reaction 11, the concentration of Parkin stays almost constant.

Starting from design 4 (i.e. in designs 4 and 5), Parkin also plays a role in the regulation of NFkB signaling. Because the concentration of Parkin is almost constant, the regulation of NFkB by Parking is negligible, unless one introduces a mutation in Parkin. This we apply in the subchapter "the genetic perturbations of regulation modes and PD". Then, the reduction of Parkin concentration has a tremendous effect.

We believe that having p62 and Parkin present throughout the designs is not only biologically relevant, but also provides consistency when moving from design to design.

In design 2 we introduced the new design feature - synthesis of healthy mitochondria. Thus, starting from design 2 there is no unlimited pool of healthy mitochondria anymore. The unlimitedness of that pool in design 1 was modelled by fixing their concentration. In the subsequent designs, at higher rates of mitochondrial damage and mitophagy, mitochondria are lost and their concentration is reduced: mitochondria is now sensitive to other parts of the network.
